# The Late Triassic Ischigualasto Formation at Cerro Las Lajas (La Rioja, Argentina): fossil tetrapods, high-resolution chronostratigraphy, and faunal correlations

**DOI:** 10.1038/s41598-020-67854-1

**Published:** 2020-07-29

**Authors:** Julia B. Desojo, Lucas E. Fiorelli, Martín D. Ezcurra, Agustín G. Martinelli, Jahandar Ramezani, Átila. A. S. Da Rosa, M. Belén von Baczko, M. Jimena Trotteyn, Felipe C. Montefeltro, Miguel Ezpeleta, Max C. Langer

**Affiliations:** 1 Consejo Nacional de Investigaciones Científicas y Técnicas (CONICET), Godoy Cruz 2290, C1425FQB Ciudad Autónoma de Buenos Aires, Argentina; 2 División Paleontología Vertebrados, Facultad de Ciencias Naturales y Museo, Universidad Nacional de La Plata, Paseo del Bosque s/n, B1900FWA La Plata, Argentina; 3 Paleontología de Vertebrados, Centro Regional de Investigaciones Científicas y Transferencia Tecnológica de La Rioja (CRILAR). Gobierno de La Rioja, UNLaR, SEGEMAR, UNCa, CONICET., Entre Ríos y Mendoza s/n, CP5301 Anillaco, Provincia de La Rioja Argentina; 4 Sección Paleontología Vertebrados, Museo Argentino de Ciencias Naturales “Bernardino Rivadavia”, Av. Ángel Gallardo 470, C1405DJR, Ciudad Autónoma de Buenos Aires, Argentina; 5 Earth, Atmospheric and Planetary Sciences, Massachusetts Institute of Technology, Cambridge, MA 02139 USA; 6 Laboratório de Estratigrafia e Paleobiologia, Departamento de Geociências, Centro de Ciências Naturais e Exatas, Universidade Federal de Santa Maria, Santa Maria, RS 97.105-900 Brasil; 7 Departamento de Biología, Departamento de Geología, Instituto de Geología (CIGEOBIO), Universidad Nacional de San Juan, Av. Ignacio de la Rosa 590 (oeste), San Juan, J5402DCS Argentina; 8 Laboratório de Paleontologia e Evolução de Ilha Solteira, Universidade Estadual Paulista, 15385-000 Câmpus de Ilha Solteira, SP Brasil; 9 Centro de Investigaciones en Ciencias de la Tierra (CICTERRA), Universidad Nacional de Córdoba, Av. Vélez Sársfield 1611, Ciudad Universitaria, Córdoba, X5016GCA Argentina; 10 Departamento de Biologia, FFCLRP, Universidade de São Paulo, Av. Bandeirantes, 3900 Ribeirão Preto, SP Brasil

**Keywords:** Palaeontology, Geology, Palaeontology

## Abstract

Present knowledge of Late Triassic tetrapod evolution, including the rise of dinosaurs, relies heavily on the fossil-rich continental deposits of South America, their precise depositional histories and correlations. We report on an extended succession of the Ischigualasto Formation exposed in the Hoyada del Cerro Las Lajas (La Rioja, Argentina), where more than 100 tetrapod fossils were newly collected, augmented by historical finds such as the ornithosuchid *Venaticosuchus rusconii* and the putative ornithischian *Pisanosaurus mertii*. Detailed lithostratigraphy combined with high-precision U–Pb geochronology from three intercalated tuffs are used to construct a robust Bayesian age model for the formation, constraining its deposition between 230.2 ± 1.9 Ma and 221.4 ± 1.2 Ma, and its fossil-bearing interval to 229.20 + 0.11/− 0.15–226.85 + 1.45/− 2.01 Ma. The latter is divided into a lower *Hyperodapedon* and an upper *Teyumbaita* biozones, based on the ranges of the eponymous rhynchosaurs, allowing biostratigraphic correlations to elsewhere in the Ischigualasto-Villa Unión Basin, as well as to the Paraná Basin in Brazil. The temporally calibrated Ischigualasto biostratigraphy suggests the persistence of rhynchosaur-dominated faunas into the earliest Norian. Our ca. 229 Ma age assignment to *Pi. mertii* partially fills the ghost lineage between younger ornithischian records and the oldest known saurischians at ca. 233 Ma.

## Introduction

**Figure 1:**
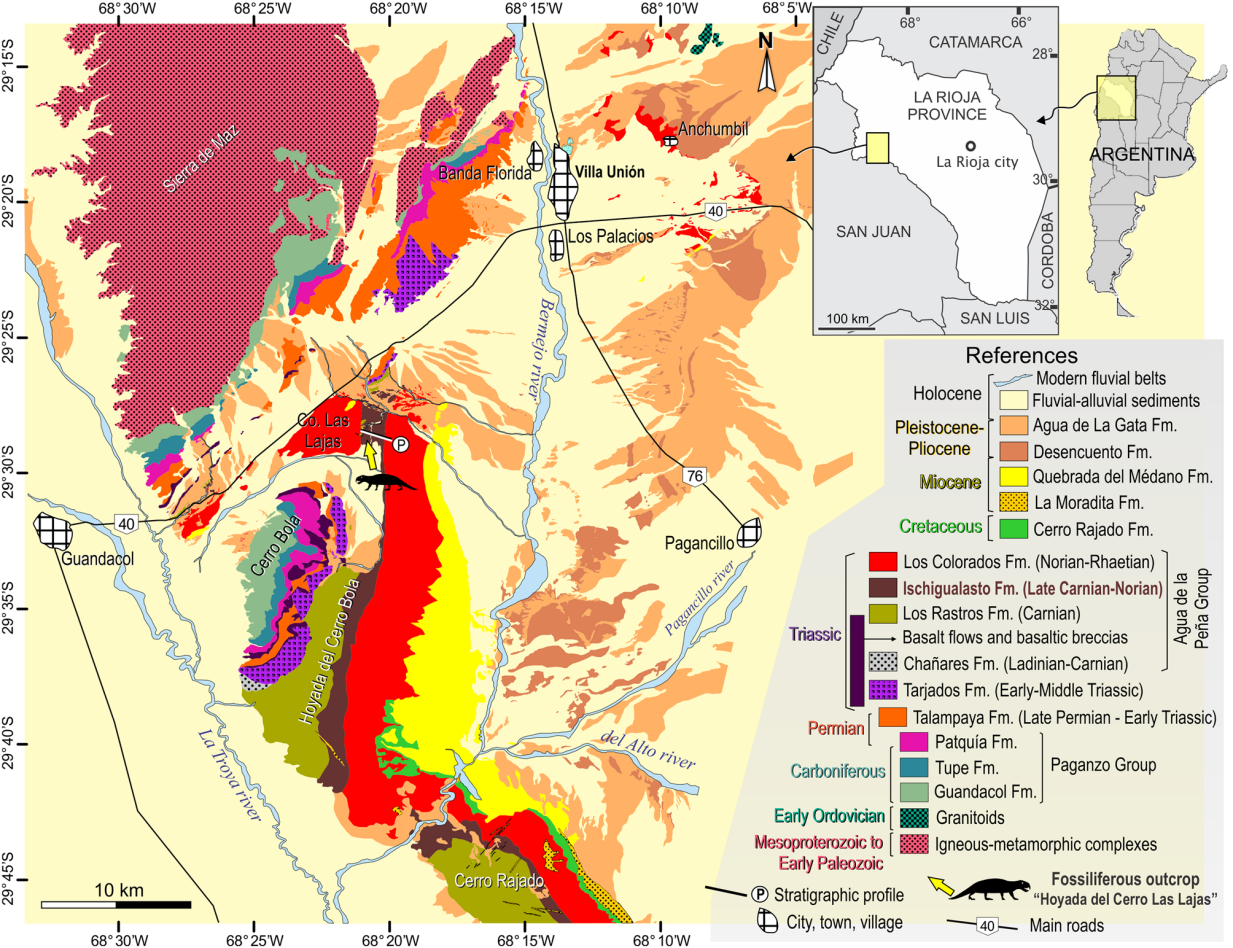
Geological map of the Bermejo Valley, La Rioja Province, northwestern Argentina. Yellow arrow points to the Hoyada del Cerro Las Lajas palaeosite. Map generated and designed by one of the authors (L.E.F.) using Corel Draw X5 software based on Google Earth imagery and our own observations and geological studies in western La Rioja. Abbreviations: Co, Cerro (hill).

With one of the richest land biotas recorded worldwide, the Ischigualasto Formation of north-western Argentina represents a unique “window” into Late Triassic biodiversity and evolution. This stratigraphic unit is well known from the Ischigualasto Provincial Park (IPP), San Juan Province, with a fossil record composed of plants, fishes, and most of the known tetrapod groups of the time, i.e., temnospondyls, rhynchosaurs, archosauriforms (including dinosaurs), dicynodonts, and cynodonts^1–3^. Radioisotopic dates of various vintages have given the Ischigualasto fauna a temporal context, elevating its global significance in understanding the Triassic land ecosystems, as well as the early evolution of dinosaurs^3^. Nevertheless, exposures of the Ischigualasto Formation outside the IPP have only been briefly explored, delivering only subordinate fossil records^3^. One exception is the site known as Hoyada del Cerro Las Lajas^4–6^ in La Rioja Province, where the northernmost known outcrops of the formation are exposed (Fig. 1; see also fig. 1 in Baczko et al*.*^7^). Explored by several expeditions starting in the early sixties (see Historical background and motivation in the Supplementary Information), the fossil record of the area appears meagre compared to that of the IPP and it has been described as “a poorly fossiliferous outcrop” (p. 20 in Martínez et al.^3^), but includes key specimens, such as the holotypes of the ornithosuchid *Venaticosuchus rusconii* and the probable ornithischian *Pisanosaurus mertii*.

Aiming to expand the fossil collections of the Hoyada del Cerro Las Lajas and to investigate the chronostratigraphic context of previous fossil collections, our team explored the Cerro Las Lajas area in the course of four expeditions from 2013 to 2019. Here, we report on more than 100 new tetrapod fossil specimens collected form the Ischigualasto Formation at Cerro Las Lajas. Detail stratigraphy of its over 1,000 m-thick succession, integrated with high-precision U–Pb zircon geochronology of three interlayered tuffs, provide a high-resolution chronostratigraphic framework for the Ischigualasto Formation in the the Hoyada del Cerro Las Lajas. In this context, we discuss palaeobiologic aspects of the Ischigualasto fauna and their implications for Late Triassic tetrapod evolution.

### Stratigraphy

**Figure 2:**
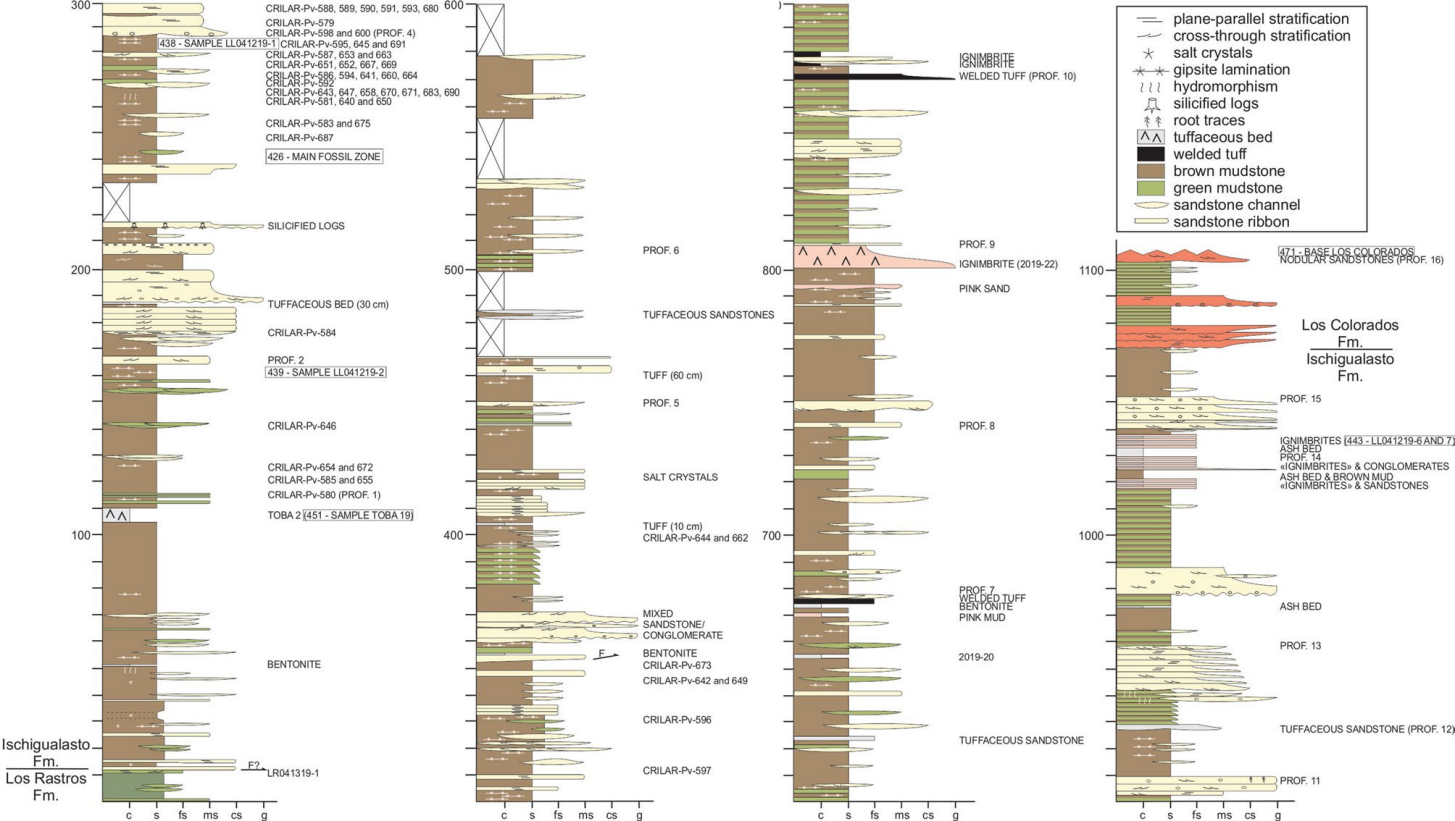
Detailed stratigraphic column of the Hoyada del Cerro Las Lajas, including positions of tetrapod fossils and dated tuff beds. Stratigraphy generated by some authors (A.A.S.D.R., J.R., M.E., and L.E.F.) using Corel Draw X5 software based on our own observations and geological studies in the Hoyada del Cerro Las Lajas.

**Figure 3:**
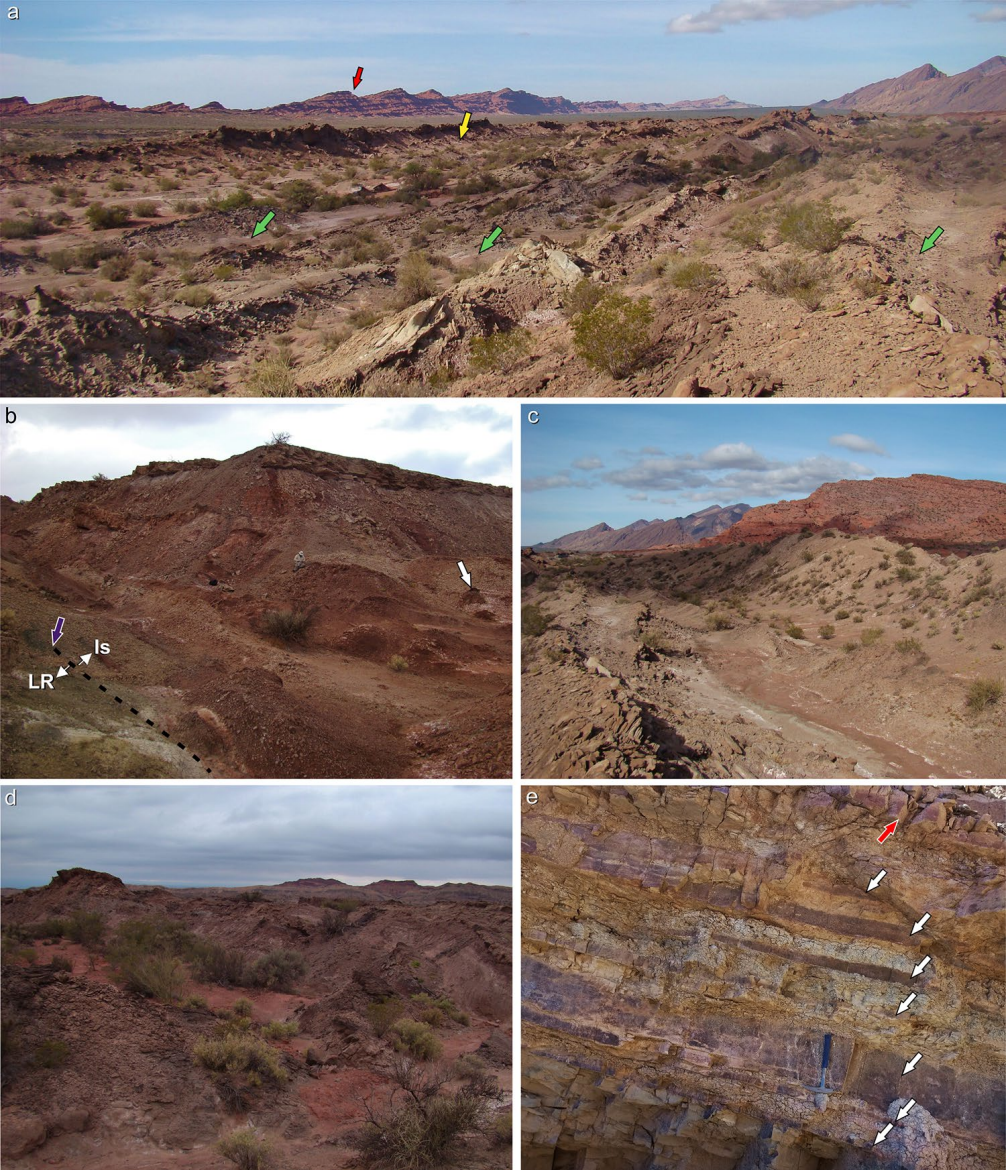
Ischigualasto Formation in the Hoyada del Cerro Las Lajas locality. (**a**) Panoramic view of Hoyada del Cerro Las Lajas, with the Los Colorados Formation (red arrow) in the background, and the *Teyumbaita* (yellow arrow) and *Hyperodapedon* (green arrows) main levels marked. (**b**) Contact between the Los Rastros Formation (olive-green) and the Ischigualasto Formation (reddish-brown) marked by dotted line and purple arrow. White arrow indicates a bentonite level at ca. 50 mab. (**c**) Strata typical of the *Hyperodapedon* biozone (ca. 150–200 mab) with the red Los Colorados Formation in the background. (**d**) Main level of *Teyumbaita* biozone, ca. 300 mab. (**e**) Interbedded tuffaceous mudstone and welded tuff (white arrows) at ca. 1,030 mab; red arrow indicates welded tuff Sample LL041219-6 (see Supplementary Fig. 3).

The section studied here is located in the “hoyada” (depression) positioned to the east of Cerro Las Lajas, southwest of the town of Villa Unión in the western La Rioja Province (Fig. 1). The lowlands of Cerro Las Lajas expose a 1,059 m-thick succession of predominantly greyish to tan siliciclastic rocks with an average dip of 30° east that belong to the Ischigualasto Formation. Floodplain siltstones and (mottled) mudstones, channel sandstones and conglomerates, and a variety of tuffs and tuffaceous sediments form the bulk of the formation. To the east, the top of the Ischigualsto succession is in transitional contact with the overlying red sandstones of the Los Colorados Formation (Figs. 2 and 3). The base of the succession to the west is juxtaposed against outcrops of the (younger) Los Colorados Formation via a steep, N–S trending fault. Discontinuous exposures of the underlying, greenish-grey Los Rastros Formation rocks occur along the fault zone, with complex stratigraphic relationships to the basal Ischigualasto strata at our measured section (see Supplementary Information). Elsewhere in the area and away from the faults, the conformable contact between the Los Rastros and Ischigualasto formations is well documented. These confirm the near-complete nature of the Ischigualasto succession exposed at the Hoyada del Cerro Las Lajas.

The succession of the Ischigualasto Formation exposed at Cerro Las Lajas is subdivided into three sections based on lithologic characteristics and alluvial depositional facies (Fig. 2) (see Supplementary Information). The lower section extending from the contact with the Los Rastros Formation—at 11 m above base (mab) of the profile in Fig. 2—to 310 mab was formed by a meandering fluvial system developed in a broad floodplain depositional setting. This section exposes an isolated, greyish-white, tuff marker bed (‘Toba-2’) up to 2 m-thick at 107 mab (i.e., 96 m above the base of the Ischigualasto Formation), which could provides a direct correlation point to the Ischigualasto Formation at the IPP (Herr Toba bentonite; see below). The majority of the fossils discovered in the Hoyada del Cerro Las Lajas occur above the tuff marker and are restricted to the lower section, with the largest concentration of fossils (main fossil zone) occurring between 240 and 300 mab (Figs. 2 and 3). The middle section (310–740 mab) marks an increase in accommodation together with fewer fossiliferous beds and more pedogenic features indicative of higher humidity conditions. The upper section (740–1,070 mab) is characterized by the transition from a meandering to a braided fluvial system concomitant with increased pyroclastic activity in the form of prominent welded tuff (ignimbrite) beds (Fig. 3e and Fig. S3d–e). The upper section is essentially currently fossil-free and extends to the base of the overlying Los Colorados Formation. The well-recognized and conformable contact between the two formations provides another correlation point between the Cerro Las Lajas succession and that of the IPP. See details of the stratigraphy of the Ischigualasto Formation at Cerro Las Lajas in the Supplemental Information file.

### Geochronology

Three samples of tuff (volcanic ash) collected from various stratigraphic levels of the Ischigualasto Formation (Fig. 2) at the Hoyada del Cerro Las Lajas (Sample Toba-2: 107 mab, Sample LL041219-2: 160 mab, and Sample LL041219-6: 1,035 mab) were successfully analysed by the U–Pb isotopic method using the chemical abrasion isotope dilution thermal ionization mass spectrometry (CA-ID-TIMS) technique at the Massachusetts Institute of Technology Isotope Laboratory. Details of sampled tuffs, analytical procedures, isotopic data, and age interpretations are given in the Supplementary Information. A Bayesian age-stratigraphic model has been employed to interpolate statistically robust ages for the stratigraphic levels of interest.

**Figure 4:**
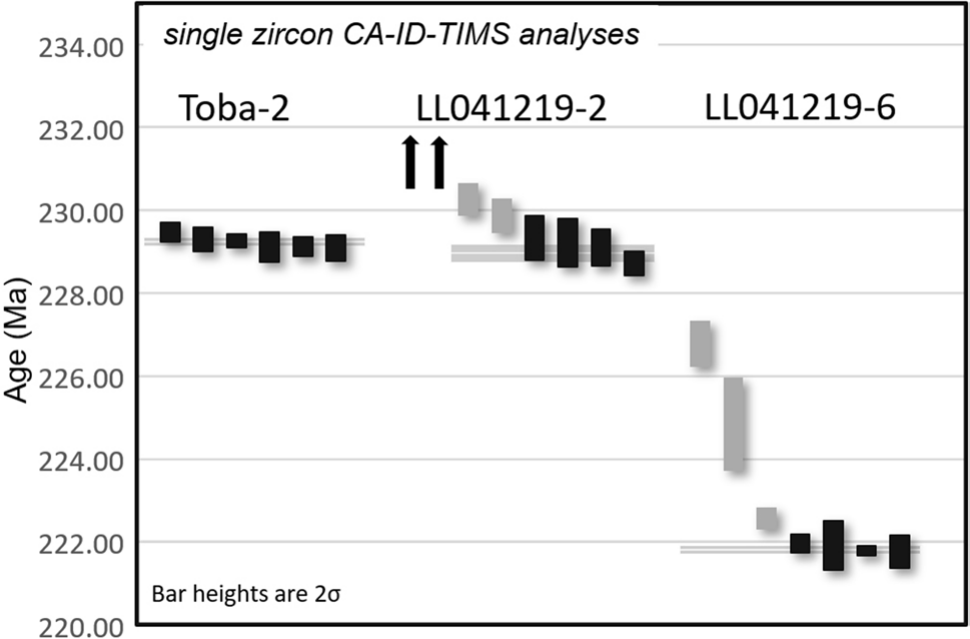
Date distribution plot of zircon CA-ID-TIMS U–Pb analyses from tuff samples of Cerro Las Lajas. Black bars are individual zircon analyses used in weighted mean age calculation. Horizontal shaded band represents the weighted mean ^206^Pb/^238^U date and its 95% confidence level internal uncertainty. Arrows point to older (detrital) analyses that fall outside the plot area. See Table 1 and Table S2 for complete U–Pb data and for details of calculated dates and their uncertianites.

Our new age model based on a set of high-precision U–Pb dates from the Hoyada del Cerro Las Lajas (Fig. 4; Table 1 and Table S2) provides a reliable chronostratigraphic framework for the Ischigualasto Formation and its Late Triassic vertebrate fauna. Accordingly, the base of the Ischigualasto Formation is constrained at 230.23 + 1.88/− 0.86 Ma. Caution must be made, however, in interpreting this age, as the limited outcrops of the underlying Los Rastros Formation at the measured section have a complex stratigraphy due to faulting (for further information, see above and the Supplementary Information). Our age model places the conformable Ischigualasto-Los Colorados formation boundary with a high degree of confidence at 221.36 + 0.44/− 1.31 Ma.

**Table 1:** Summary of calculated U-Pb ages and their uncertainties.

Sample	Formation	Height above base (m)	Latitude (S)	Longitude (W)	^206^Pb	date	Uncertainty (2σ)			MSWD	*n*	#
					^238^U		*X*	*Y*	*Z*			
LL041219-6	Ischigualasto	1035	29°28'43.29"	68°20'12.81"	221.82		0.10	0.12	0.27	0.63	4	7
LL041219-2	Ischigualasto	160	29°28'42.53"	68°20'57.64"	228.97		0.22	0.23	0.33	1.9	4	8
Toba-2	Ischigualasto	107	29°28'33.00"	68°21'1.04"	229.25		0.10	0.16	0.30	1.2	6	6

Notes: Latitude/Longitude relative to WGS84 datum. *X* internal (analytical) uncertainty in the absence of all external or systematic errors. *Y* incorporates the U-Pb tracer calibration error. *Z* includes *X* and *Y*, as well as the uranium decay constant errors. *MSWD* mean square of weighted deviates. *n* number of analyses included in the calculated weighted mean date, out of a total number of # analyses.

**Figure 5:**
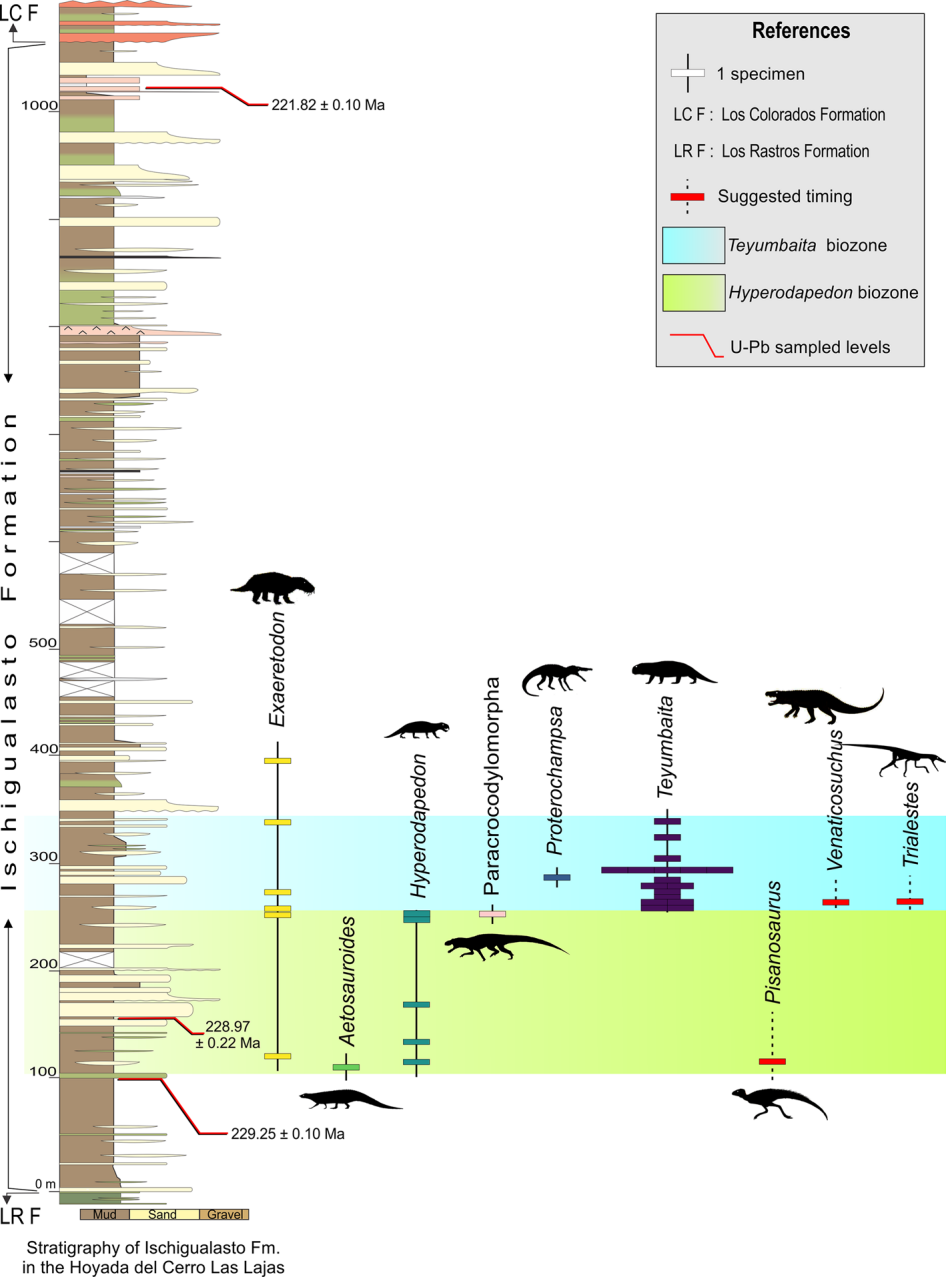
Stratigraphic column of the Ischigualasto Formation at the Hoyada del Cerro de Las Lajas with ranges of occurrence of its main taxa concentrated between 100 and 350 m above base. Figure generated by some authors (A.A.S.D.R., J.R., M.E., and L.E.F.) using Corel Draw X5 software based on our own geological studies in the region.

Vertebrate fossils at the Hoyada del Cerro Las Lajas have so far been limited to the lower part of the Ischigualasto Formation (lower and middle sections), from ~ 115 to 400 mab. The fossil record starts shortly above the dated ‘Toba-2’ tuff marker at 229.25 ± 0.10/0.16/0.30 Ma; a stratigraphic relationship that mimics that of the Herr Toba bentonite at IPP^3, 8^. The main fossil concentration, however, occurs between 240 and 300 mab (Figs. 2 and 5), which is constrained in time between 228.10 + 0.72/− 1.50 and 227.61 + 1.04/− 1.79 Ma, based on our Bayesian age model. As such, it roughly coincides with the extrapolated age of the Carnian-Norian stage boundary, which has been indirectly calibrated at ~ 227 Ma (updated)^9^ or ~ 228.4 Ma^10^.

### Fossil provenance

Consistent with observations in José Bonaparte field notes and photographs (see details in Supplementary Materials), the fossiliferous beds at the Hoyada del Cerro Las Lajas are mostly concentrated in the lower third of the studied succession, between 100 and 400 mab (Figs. 2 and 5). The lowermost recovered fossil corresponds to the aetosaur *Aetosauroides scagliai* (CRILAR-Pv 580) that occurs at 115 mab (Figs. 2 and 5). Further upsection, a specimen representing a new species of the rhynchosaur *Hyperodapedon* (CRILAR-Pv 585) was collected in brown mudstones, near a specimen of the cynodont *Exaeretodon* sp. (CRILAR-Pv 672) at about 120–125 mab. *Hyperodapedon sanjuanensis* also occurred as isolated finds at 140 (CRILAR-Pv 646) and 175 (CRILAR-Pv 584) mab.

The zone of main fossil concentration (240–300 mab) starts with indeterminate rhynchosaur records (CRILAR-Pv 675, 687) and *H. sanjuanensis* (CRILAR-Pv 583), with the uppermost record of that genus and species (CRILAR-Pv 650) occurring near 260 mab. This is slightly below the lowest record, at 265 mab, of *Teyumbaita* (CRILAR-Pv 592, 643) in the succession. The latter genus accounts for fourteen out of the twenty fossil specimens identified at the genus level in this 60 m interval, also occurring as high as 350 mab. The range of *Exaeretodon* spans across that main fossil zone, but with a lower abundance (three specimens). The only record of Paracrocodylomorpha (CRILAR-Pv 581) occurs at 260 mab, whereas that of *Proterochampsa barrionuevoi* (CRILAR-Pv 579) is at 290 mab (Fig. 5).

Few fossils have been collected from above 300 mab. *Teyumbaita* (CRILAR-Pv 597, 642) was found at 310 and 350 mab, along with *Exaeretodon* (CRILAR-Pv 649). This cynodont marks the stratigraphically highest fossil identified to the genus level (CRILAR-Pv 644) at 400 mab, but an indeterminate rhynchosaur tooth plate (CRILAR-Pv 662) was found at the same level. A complete list of specimens with their geographic provenance and stratigraphic positions are provided in the Supplementary Material Table S1.

Petrographic analyses of rock matrices from legacy fossil collections without exact location data have been used to infer their stratigraphic positions in the context of our new chronostratigraphic framework (see details in Supplementary Materials). Accordingly, *Pi. mertii* is speculated to have been recovered from between 110 and 180 mab in the lower section of the Ischigualasto Formation and below the main fossil zone. Similarly, the positions of *V. rusconii* and *Tri. romeri* are inferred to be ~ 270 mab, within the main fossil zone (Fig. 5).

### Systematic palaeontology

- Cynodontia Owen, 1861^11^.
  Eucynodontia Kemp, 1982^12^.
- Traversodontidae Huene, 1936^13^ sensu Kammerer et al., 2008^14^.
- *Exaeretodon* Cabrera, 1943^15^.
- *Exaeretodon* sp.

#### Material.

CRILAR-Pv 640, portion of right lower postcanine, portion of humeral distal condyle, and indeterminate bone fragments. CRILAR-Pv 644, badly preserved partial skull with two upper postcanine teeth. CRILAR-Pv 647, partial postcranium, including several presacral, sacral, and caudal vertebrae, ribs, and most of the fore- and hindlimbs, plus unprepared plaster jackets. CRILAR-Pv 649, partial skull in three parts bearing some badly preserved postcanine teeth, lacking most of the snout and braincase, posterior fragment of right dentary with two postcanine teeth, right postcanine tooth, fragment of left dentary with one postcanine tooth and part of a root, two partial incisors, two partial upper canines, and some indeterminate bone fragments. CRILAR-Pv 663, partial skull still in plaster jacket. CRILAR-Pv 672, fragment of bone with natural cast of quadrangular tooth mixed with rhynchosaur remains. Three other cynodont specimens (i.e., vertebral centra, long bones fragments) were collected during the field works reported here, but their incompleteness hampers a more detailed classification.

#### Description.

**Figure 6:**
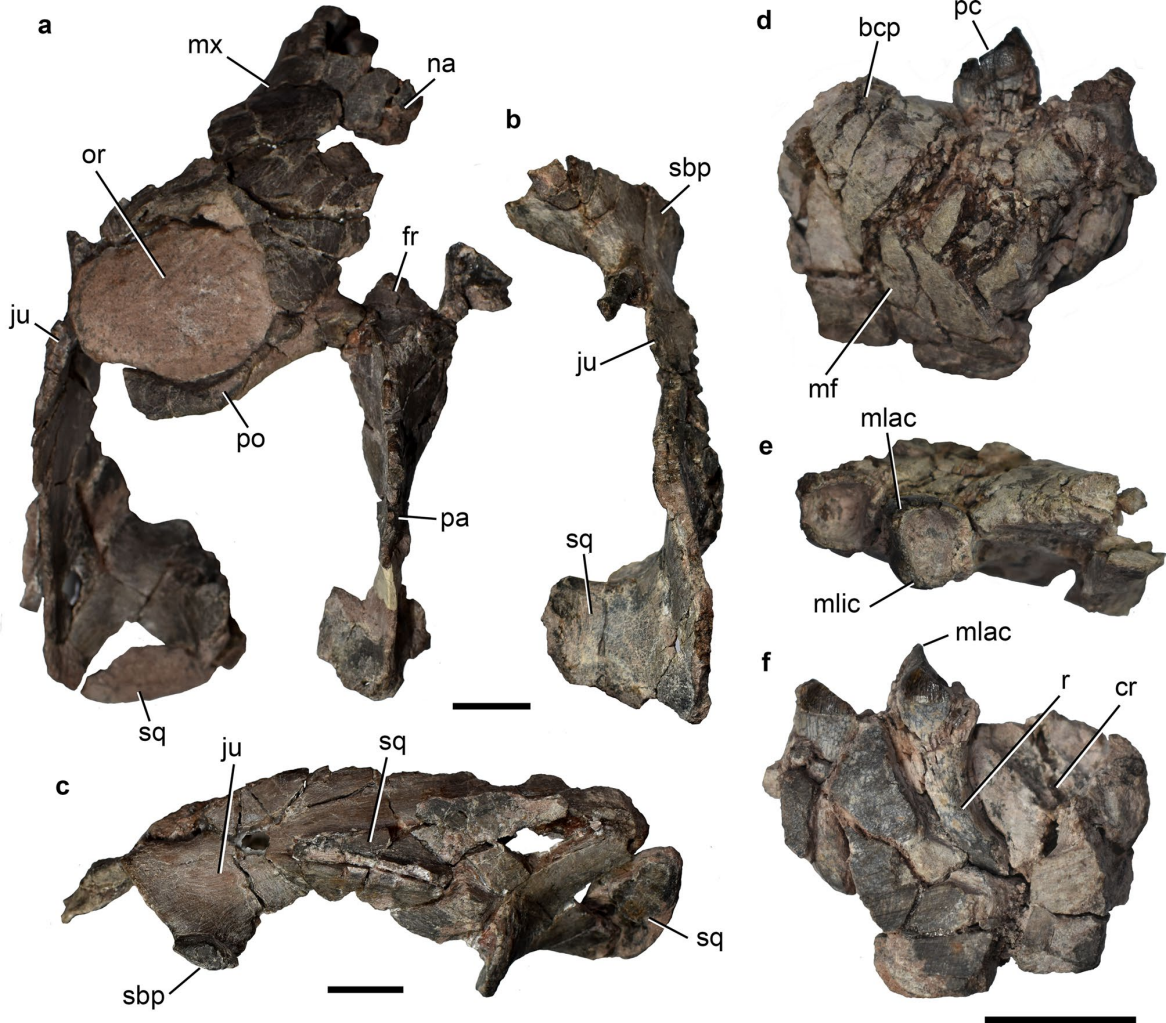
*Exaeretodon* sp. CRILAR-Pv 649, Ischigualasto Formation, Cerro Las Lajas, La Rioja Province. (**a**) Partial skull with (**b**) its isolated right zygomatic arch in dorsal view, and (**c**) detail of the left zygomatic arch in lateral view. Fragment of right dentary with two posterior postcanines in (**d**) lateral, (**e**) occlusal, and (**f**) medial views. Abbreviations: bcp, base of coronoid process; cr, crypt; fr, frontal; ju, jugal; mf, masseteric fossa; mlac, mesiolabial cusp; mlic, mesiolingual cusp; mx, maxilla; na, nasal; or, orbit; pa, parietal; pc, lower postcanine tooth; po, postorbital; sbp, suborbital process of jugal; sq, squamosal; r, root. Scale bars: 2 cm.

Of the specimens referred to *Exaeretodon*, the most complete cranial material belongs to CRILAR-Pv 647 (Fig. 6), but it is badly preserved. It corresponds to a small-sized skull, with an estimated skull length of ~ 20 cm. It is fragmented in three pieces, including: (1) part of the left portions of snout, orbit, the postorbital bar, the zygomatic arch and the parietal crest (Fig. 6a); (2) part of the right zygomatic arch, with the anteroventral edge of the orbit and the ventral base of the postorbital bar (Fig. 6b); and (3) part of the palate including portions of maxillae with poorly preserved posterior postcanines, palatines, and pterygoids. As in other specimens of *Exaeretodon* (e.g., MACN-Pv 18125; Bonaparte^16^), the zygomatic arch is deep with the anterior process of the squamosal anteriorly extended almost to the level of the postorbital bar and there is a well-developed descending process of the jugal below the orbit (Fig. 6c). The orbit (preserved only in the left side) is relatively large, in accordance with the small, sub-adult size of the skull, and it faces mostly dorsally due to taphonomic dorsoventral compression (Fig. 6a). The parietal crest is incomplete, but appears to have been well-developed. The palatal area is poorly preserved, with the primary palate exposed lateral to the last postcanine teeth as in other traversodontids^16,17^, due to the presence of an axially short secondary palate. The crown morphology of the upper postcanines is difficult to access, but it is typically divided in two (labial and lingual) lobes, with an occlusal basin, and an extensive “V” shaped shouldering.

The mandibular elements and lower dentition are also poorly preserved (Fig. 6d–f). A right dentary fragment preserves two postcanines and the crypt for a posterior one (possibly in process of eruption at the moment of death). That dentary preserves part of its straight ventral edge, the anterior end of the masseteric fossa, and the base of the coronoid process (Fig. 6d). The masseteric fossa seems to be well-developed and reaches the level of the last functional postcanine. The base of the coronoid process is robust and laterally covers the crypt in the area where postcanines are added to the tooth row during ontogeny (Fig. 6f). Another dentary fragment only holds a left postcanine and part of the root of the posterior one.

The lower postcanine teeth of CRILAR-Pv 647 have the typical shape observed in *Exaeretodon* (e.g., MACN-Pv 18125)^18,19^. They are subquadrangular in occlusal view, with a deep central occlusal basin surrounded by four cusps placed at each corner (Fig. 6e). The mesiolabial cusp is the largest and both mesial cusps (mesiolabial and mesiolingual) are in a higher position relative to the distal ones, forming a mesial cutting edge slightly apicodistally inclined (Fig. 6e,f). The crown is relatively low and surrounded by a thick layer of enamel, which does not cover the occlusal surface. The limit between crown and root is not marked by a neck, but by the edge of the enamel layer. The root of each tooth is conical and tapers toward the apex. The apex is distally curved (Fig. 6f) as typical of *Exaeretodon* (UFRGS-PV 1096-T)^20^, as the result of the tooth replacement.

There are two partially preserved canines of small size in CRILAR-Pv 647, which are interpreted as lower elements. Their crown is sub-conical, slightly labio-lingually flattened, and well curved distally. The incisors are represented by three crown fragments of small size. One is attached to the palate fragment and the other two are isolated. They have the labial surface strongly convex and the lingual one flat to slightly convex, with the crown slightly curved lingually.

#### Comments.

The specimens of *Exaeretodon* from the Hoyada del Cerro Las Lajas are fragmentary and poorly preserved in comparison to those discovered at the Hoyada de Ischigualasto, in San Juan Province^2, 3,16^. Only CRILAR-Pv 647, which is still under preparation (and will be described elsewhere), includes a well preserved postcranium, but apparently lacks cranial elements. Moreover, cynodonts are not as abundant as archosauromorphs (mainly rhynchosaurs) in the area and are taxonomically restricted so far to the traversodontid *Exaeretodon*.

*Exaeretodon* was first described^25^ based on specimens collected in the Hoyada de Ischigualasto. After several taxonomical revisions^21,22^, *Ex. argentinus* is regarded as the only valid species known from the Ischigualasto Formation. The genus is also recognized in the *Hyperodapedon* Assemblage-Zone of the Candelária Sequence, Santa Maria Formation, Brazil, represented by *Ex. riograndensis*^17,19^, and in the lower Maleri Formation of India, represented by *Ex. statisticae*^23^.

Presently, the Hoyada del Cerro Las Lajas specimens can be clearly assigned to the genus *Exaeretodon*. Nonetheless, the lack of complete skulls precludes the evaluation of features that may distinguish between *Ex. argentinus* and *Ex. riograndensis*, such as the prootic crests and the postcanine variation along ontogeny. Also, amongst the specimens traditionally referred to *Exaeretodon* from the Hoyada de Ischigualasto, two different morphotypes were recently briefly communicated^24^, one of which seems to be closely related to the recently described traversodontid *Siriusgnathus niemeyerorum* from the Candelária Sequence of Rio Grande do Sul, Brazil^25^. The specimens collected in La Rioja do not have the combination of features observed in *Sir. niemeyerorum* (CAPPA/UFSM 0032), e.g., a very reduced suborbital process of the jugal^25^. Consequently, the traversodontid material from the Hoyada del Cerro Las Lajas fits better with the genus *Exaeretodon* and only further material will allow elucidating their taxonomy at a specific level.

- Rhynchosauria Osborn, 1903^26^ sensu Ezcurra^27^.
- Hyperodapedontinae Lydekker, 1885^28^ sensu Langer & Schultz^29^.
- *Hyperodapedon* Huxley, 1859^30^.
- *Hyperodapedon sanjuanensis* Sill, 1970^31^.

#### Material.

**Figure 7:**
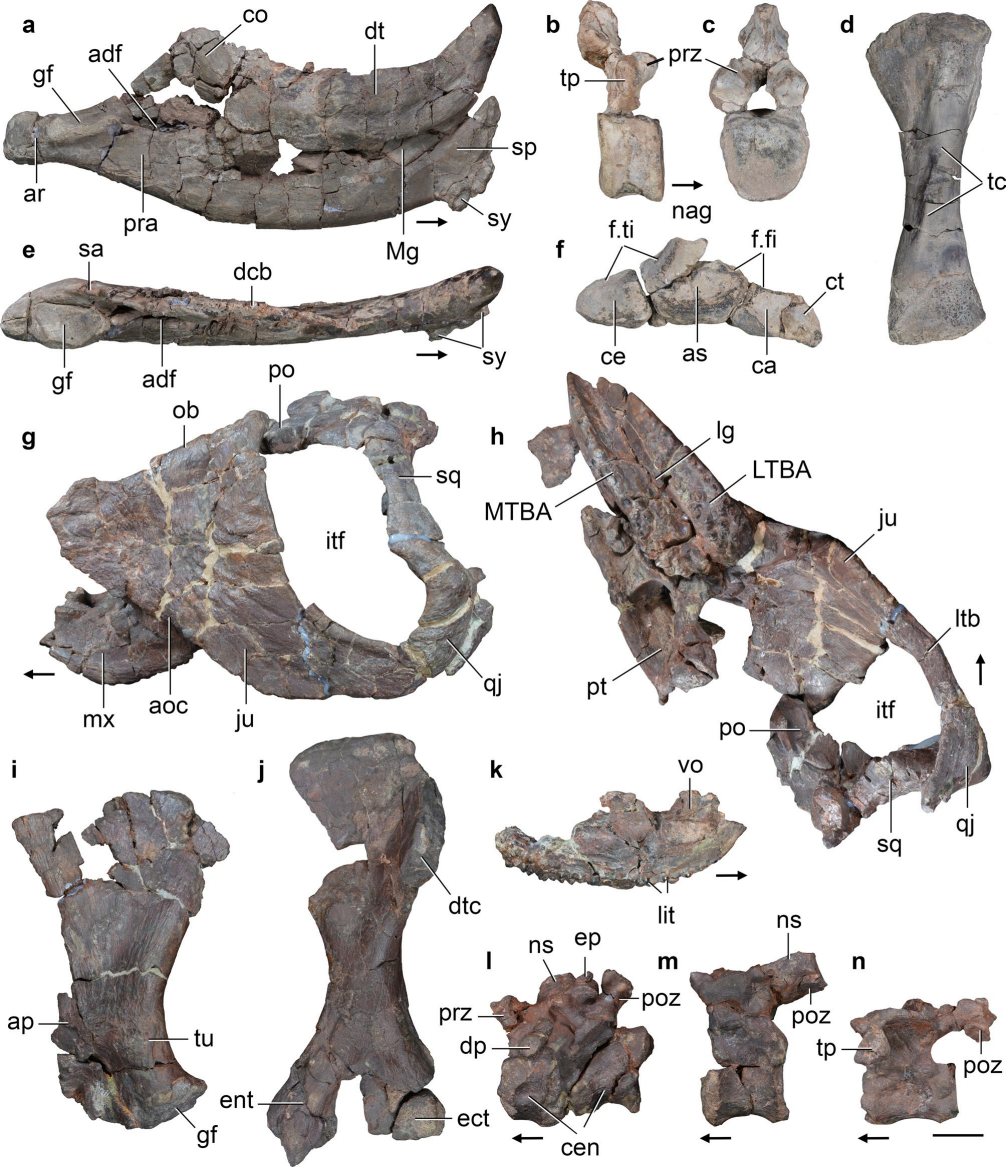
*Hyperodapedon* spp. Selected bones of specimens (**a**, **d**, **e**) CRILAR-Pv 583, (**b**, **c**, **f**) CRILAR-Pv 584, and (**g**–**n**) CRILAR-Pv 585 referred to (**a**–**f**) *H. sanjuanensis*, and (**g**–**n**) *Hyperodapedon* sp. nov. collected in the outcrops of the Ischigualasto Formation immediately east to Cerro Las Lajas. (**a**, **e**) Left hemimandible, (**b**, **c**) anterior dorsal vertebra, (**d**) left tibia, (**f**) proximal tarsals, (**g**, **h**) partial skull, (**i**) left scapula, (**j**) left humerus, (**k**) left maxilla, (**l**) two articulated anterior postaxial cervical vertebrae, (**m**) posterior cervical vertebra, and (**n**) anterior-middle dorsal vertebra in (**a**, **k**) medial, (**b**) right lateral, (**c**, **d**, **f**, **j**) anterior, (**e**) dorsal, (**g**, **l**–**n**) left lateral, (**h**) ventral, and (**i**) lateral views. Arrows indicate anterior direction. Abbreviations: adf, adductor fossa; aoc, anguli oris crest; ap, acromial process; ar, articular; as, astragalus; ca, calcaneum; ce, centrale; cen, centra; co, coronoid; ct, calcaneal tuber; dcb, dentary cutting blade; dp, diapophysis; dt, dentary; dtc, deltopectoral crest; ect, ectepicondyle; ent, entepicondyle; ep, epipophysis; f.fi, facet for fibula; f.ti, facet for tibia; gf, glenoid fossa; itf, infratemporal fenestra; ju, jugal; lg, longitudinal groove; ltb, lower temporal bar; LTBA, lateral tooth bearing area; Mg, Meckelian groove; mx, maxilla; MTBA, medial tooth bearing area; nag, non-articular gap; ns, neural spine; ob, border of orbit; po, postorbital; poz, poszygapophysis; pra, prearticular; prz, prezygapophysis; pt, pterygoid; qj, quadratojugal; sa, surangular; sp, splenial; sq, squamosal; sy, symphysis; tc, tibial crest; tp, transverse process; tu, tuberosity. Scale bar: 2 cm.

CRILAR-Pv 583, fairly complete left hemimandible, left tibia, probable distal tarsal, and probable left metacarpal (Fig. 7a, d, e). CRILAR-Pv 584, left maxilla without anterior portion, right dentary with damaged posterior end, left articular and partial prearticular, an anterior dorsal vertebra, two dorsal centra, a fragment of probable caudal centrum, a right and a left postzygapophysis, articulated left centrale, astragalus, and calcaneum, two distal tarsals, proximal end of right metatarsals II and III, five non-ungual phalanges, an ungual phalanx lacking its distal end, and indeterminate bone fragments (Fig. 7b, c, f). CRILAR-Pv 646, partial left dentary, missing its posterior edge and anterior half, and left tibia. CRILAR-Pv 650, right dentary and partially prepared partial cranium.

#### Description.

The occlusal surface of the maxillary tooth plate is subdivided by a single, medially displaced longitudinal groove, as occurs in *H. mariensis* (MCN 1867-PV), *H. huxleyi*^32^, the morphotype 1 of *H. tikiensis*^33^, *Supradapedon stockleyi*^34^, *Isalorhynchus genovefae*^35^, unnamed hyperodapedontines from Zimbabwe^36^ and Nova Scotia^37^, and other specimens of *H. sanjuanensis*^38^. By contrast, the single longitudinal groove is approximately centred on the tooth plate in *H. gordoni*^39^ and an indeterminate hyperodapedontine from the Ischigualasto Formation^40^, whereas there are two longitudinal grooves in *H. huenei*^29^ and *Teyumbaita sulcognathus*^41^. The lateral tooth bearing area (LTBA) has five longitudinal tooth rows and the crowns in the two medialmost rows (L1 and L2) are worn to the root, resembling the condition of most hyperodapedontines (e.g. *H. mariensis*, UFRGS-PV 0149T, 0408T; *H. huxleyi*^32^). By contrast, only one or two longitudinal tooth rows are present in the LTBA of North American hyperodapedontines (Wyoming form^42^; Nova Scotia form^37^), *Te. sulcognathus*^41^, and *I. genovefae*^35^. The number of rows in the medial tooth bearing area (MTBA) and presence of lingual teeth cannot be determined in CRILAR-Pv 584 because of damage.

The dentary forms more than half of the hemimandible, has a tapering anterior end, and does not form part of the mandibular symphysis, which are character-states retained by all hyperodapedontines^43–45^ (Fig. 7a, e). The dentary has a single, transversely thin cutting blade with one row of mesiodistally compressed teeth. There is no lingual teeth and no medially bulged area in the dentaries of CRILAR-Pv 583, 584, 646, 650, as typical of *H. sanjuanensis*^38^ and also reported for an unnamed hyperodapedontine from Nova Scotia^37^. By contrast, all other hyperodapedontines have lingual teeth on the dentary^34,38^. A well-developed coronoid prominence is formed by the dentary, surangular, and coronoid bones. A deep and lateroventrally opened posterior surangular foramen is located at level with the glenoid fossa. The retroarticular process is short and its dorsal surface is damaged in CRILAR-Pv 583.

The anterior dorsal vertebra possesses a spool-shaped, taller than long centrum that lacks a ventral keel (Fig. 7b, c). The neural spine is restricted to the posterior two-thirds of the neural arch and does not extend between the bases of the prezygapophyses. The tibia has transversely expanded proximal and distal ends (Fig. 7d). The shaft possesses a well-developed, proximolaterally to distomedially oriented tibial crest on its anterior surface, as occurs in other rhynchosaurids^43,46,47^. The distal articular surface of the bone is transversely convex and slants proximolaterally. The proximal row of tarsals is composed of a centrale, astragalus, and calcaneum, as occurs in other rhynchosaurs^48^ (Fig. 7f). The centrale is not fused to the astragalus and its proximal surface extensively contributes to the tibial facet. The proximal surface of the astragalus has tibial and fibular facets separated from one another by a non-articulating gap. The posterior surface lacks a posterior groove and the autapomorphic transverse boss present in *Te. sulcognathus*^46^. The medial half of the proximal surface of the calcaneum is occupied by the fibular facet and the lateral half of the bone is developed as a laterally projected calcaneal tuber. The calcaneal tuber is anteroposteriorly narrower than proximodistally tall and the latter axis is rotated approximately 45° from the proximodistal plane of the proximal tarsus.

#### Comments.

These specimens can be referred to Hyperdapedontinae because of the presence of the following synapomorphies^34^: mandible dorsoventral depth > 0.25 times its total length (CRILAR-Pv 583); dentary with mesiodistally compressed teeth (all specimens); posteriormost dentary teeth on the posterior half of the lower jaw (CRILAR-Pv 583); and astragalus with centrale facet greater than the tibial facet (CRILAR-Pv 584, although this condition is unknown in the immediate sister-taxa to Hyperodapedontinae). In addition, CRILAR-Pv 584 can be included in the *Hyperodapedon* clade^44^ because of the presence of a maxillary tooth plate with more than two tooth rows in the LTBA and four or more tooth rows on its anterior half. Within Hyperodapedontinae, these specimens can be referred to *H. sanjuanensis* because the absence of lingual teeth in the dentary has been considered an autapomorphy of this species^38^. Nevertheless, the unnamed Nova Scotia hyperodapedontine apparently also lacks lingual dentary teeth^37^ and this feature may be an apomorphy of a more inclusive clade of hyperodapedontines (i.e. *H. sanjuanensis* + North American forms^34^). We preferred here to maintain this character-state as diagnostic of *H. sanjuanensis* until more information of the Nova Scotia hyperodapedontine is published. In any case, the maxillary tooth plate of CRILAR-Pv 584 differs from those of the Nova Scotia hyperodapedontine in the presence of a higher number of tooth rows in the LTBA and, at least for this specimen, such combination of character-states still supports its referral to *H. sanjuanensis*.

- *Hyperodapedon* sp. nov.

#### Material.

CRILAR-Pv 585, articulated partial left side of cranium missing lacrimal, prefrontal, anterior region of palate and almost entirely the skull table; fragment of right maxilla; partial braincase; at least 14 postaxial cervical and anterior-middle dorsal vertebrae; several ribs and gastralia; both scapulae; left humerus; distal end of right humerus; and multiple indeterminate bone fragments (Fig. 7g–n).

#### Description.

The overall morphology of the skull of CRILAR-Pv 585 resembles that of other hyperodapedontine rhynchosaurs in the presence of a ventral border of the orbit positioned dorsal to the mid-height of the infratemporal fenestra, a massive and anterodorsally-to-posteroventrally sloping jugal, and a closed lower temporal bar (e.g. *I. genovefae*^45^; *H. mariensis*, MCN 1867-PV; *H. huenei*^29^; *H. sanjuanensis*, MACN-Pv 18185; *Te. sulcognathus*^41^) (Fig. 7g,h). The infratemporal fenestra is kidney-shaped, with a notched posterior border. This outline is a result of the strongly concave anterior margin of the ascending process of the quadratojugal, as occurs in *H. huenei*^29^ and *H. huxleyi*^32^. By contrast, this margin is approximately straight in the holotype of *H. sanjuanensis* (MACN-Pv 18185), *Te. sulcognathus*^41^, and *I. genovefae*^45^, and convex in *H. gordoni*^39^.

The occlusal surface of the maxillary tooth plate of CRILAR-Pv 585 is divided into equally broad LTBA and MTBA by a longitudinal groove (Fig. 7h), as also occurs in *H. gordoni*^39^ and an indeterminate hyperodapedontine from the Ischigualasto Formation^40^. By contrast, the maxillary tooth plate has a broader LTBA in all other hyperodapedontines with a single groove^34^. The maxillary tooth plate of CRILAR-Pv 585 also differs from those of *Te. sulcognathus*, *H. huenei*, and the morphotype 2 of *H. tikiensis*, which possess two longitudinal grooves that define a third, central tooth bearing area^29, 33,41^. CRILAR-Pv 585 has four longitudinal tooth rows at the posterior end of the LTBA and three rows in the MTBA. In addition, there is a row of six lingual teeth, well-spaced from one another and located on the medial surface of the maxilla, dorsally to the MTBA (Fig. 7k), resembling the condition in *H. huenei*^29^ and a Zimbabwean hyperodapedontine^36^. However, CRILAR-Pv 585 differs from these two forms in the presence of lingual tooth crowns that are mainly oriented ventrally rather than perpendicular to the occlusal surface, and from all rhynchosaurs in the presence of lingual teeth restricted to the anterior half of the tooth plate.

The jugal forms the ventral border of the orbit and bears an anterodorsally-to-posteroventrally oriented anguli oris crest that overhangs laterally the maxilla (Fig. 7g). The lateral surface of the jugal is coarsely ornamented by low ridges and bulges on its main body and striations adjacent to the orbital edge. No secondary anguli oris crest is present on the main body of the jugal, contrasting with *Te. sulcognathus*^41^ and *I. genovefae*^45^. The lateral surface of the posterior process of the jugal of CRILAR-Pv 585 lacks the deep and posterodorsally well-rimmed depression located on the ventral half of the base of this process in *H. huxleyi* (ISIR 01), *H. huenei* (UFRGS-PV 0132T), and referred specimens of *H. mariensis* (UFRGS-PV 0149T). The posterior process of the jugal forms the entire ventral border of the infratemporal fenestra in CRILAR-Pv 585, as occurs in *H. huenei*^29^ and *Te. sulcognathus*^41^. By contrast, the anterior process of the quadratojugal contributes to the ventral border of the opening in *I. genovefae*^45^, the holotype of *H. sanjuanensis* (MACN-Pv 18185), *H. mariensis* (UFRGS-PV 0149T), *H. gordoni*^39^, and *H. huxelyi* (ISIR 01).

The palatine of CRILAR-Pv 585 contacts the ectopterygoid posterolaterally and, as a result, excludes the maxilla from the border of the infraorbital foramen. The pterygoid possesses a cup-shaped, dorsomedially projected process that received the basipterygoid process of the parabasisphenoid. This facet indicates the presence of a basal articulation two times dorsoventrally taller than transversely broad, as occurs in *Te. sulcognathus*^41^ and other species of *Hyperodapedon*^45^.

The basioccipital possesses a long occipital neck and basal tubera broadly separated from one another. The exoccipital contacted its counterpart on the floor of the endocranial cavity, as occurs in several other hyperodapedontines (e.g. *H. huenei*, UFRGS-PV 0132T; *H. mariensis*, UFRGS-PV 0149T; *H. sanjuanensis*, MACN-Pv 18185), but contrasting with the absence of such contact in *Te. sulcognathus*^41^. The occipital surface of the base of the paroccipital process possesses a ventrally well-defined depression on its dorsal half, resembling a condition previously reported as autapomorphic of *Te. sulcognathus*^41^.

The postaxial cervical vertebrae have a spool-shaped centrum that lack a ventral keel and possess a shallow depression on its dorsolateral surface (Fig. 7l). By contrast, the anterior-middle cervical vertebrae of *Te. sulcognathus* have a median ventral keel^46^. There is a tall, crest-shaped (i.e. conical) epipophysis on the dorsal surface of the postzygapophysis, perhaps absent only in the posteriormost cervical vertebrae (Fig. 7m). The neural spine is restricted to the posterior half of the neural arch. The centra of the anterior and middle dorsal vertebrae are generally longer and slightly more transversely compressed than the cervical centra (Fig. 7n). The anterior-middle dorsal neural arches possess comma-shaped transverse processes in cross-section and lack laminae. The postzygapophyses lack an epipophysis.

The scapula is anteroposteriorly expanded at both the proximal and distal ends (Fig. 7i). The very base of the acromial process is thick, ridge-like and distinctly laterally raised, resembling the condition in most hyperodapedontines (e.g., *H. sanjuanensis*, MACN-Pv 18185; *H. huxleyi*, ISIR 01; *H. tikiensis*^33^). By contrast, this process is sub-circular and blunt in *Te. sulcognathus*^46^. The scapular blade has distinctly divergent anterior and posterior margins, as occurs in most hyperodapedontines (e.g., *H. huxleyi*^32^; *H. mariensis*, MCN 1867-PV), but the scapular blade possesses a tab-like, poorly developed posterior expansion in *Te. sulcognathus* (UFRGS-PV 0232T). The proximal and distal ends of the humerus are distinctly transversely expanded and their main axes rotated approximately 40° from one another (Fig. 7j). The deltopectoral crest is mainly anteriorly oriented. The distal end has a very deep, subtriangular, and concave anterior fossa and a shallower and more proximally extended posterior fossa. The lateral surface of the distal end possesses a deep longitudinal ligament groove (= ectepicondylar groove) that is anteriorly delimited by a supinator ridge, resembling the condition in other hyperodapedontines (e.g., *H. tikiensis*^33^; *H. huxleyi*^32^; *H. gordoni*^39^; *H. sanjuanensis*, MACN-Pv 18185; *Te. sulcognathus*, UFRGS-PV 0232 T).

**Comments.** CRILAR-Pv 585 is identified as a hyperodapedontine rhynchosaur because of the presence of the following synapomorphies of the clade^34^: jugal without an elevated orbital rim; fully closed lower temporal bar; anguli oris crest extended onto the anterior process of the jugal, but not the maxilla; maxilla well laterally overlapped by the jugal; maxillary tooth plate with cushion-shaped LTBA; and maxillary teeth with conical and ‘pyramidal’ crowns. In addition, CRILAR-Pv 585 shares with other members of the *Hyperodapedon* clade the following synapomorphies^34^: maxillary tooth plate with more than two tooth rows in the MTBA; maxillary tooth plate with four or more tooth rows of occlusal teeth on its anterior half; parabasisphenoid with a basipterygoid process wider than long (inferred from the shape of the basal articulation on the pterygoid); and postaxial cervical postzygapophyses with crest-shaped epipophysis. Among hyperodapedontines, CRILAR-Pv 585 differs from other taxa in the presence of an autapomorphic row of ventrally oriented lingual teeth restricted to the anterior half of the maxillary tooth plate. This new species will be formally named and described in detail in a future contribution.

- *Hyperodapedon* sp.

#### Material.

CRILAR-Pv 582, isolated right partial maxilla, fragment of right occlusal blade of right dentary (originally in occlusion with the maxilla), and an indeterminate partial bone.

#### Description and comments.

The maxilla of CRILAR-Pv 582 is represented by the middle portion of the tooth plate. The occlusal surface of the bone is subdivided by a single, medially displaced longitudinal groove, as occurs in *H. sanjuanensis*, *H. mariensis*, the holotype of *H. tikiensis*, *H. huxleyi*, *Su. stockleyi* and *I. genovefae*^32,34,38,49^. By contrast, the longitudinal groove is centred on the tooth plate in *H. gordoni*, the above *Hyperodapedon* sp. nov., and an indeterminate hyperodapedontine from the Ischigualasto Formation^40^. The longitudinal groove narrows anteriorly, resembling the condition in some other hyperodapedontines (e.g. UFRGS-PV 0149T, 0408T). The longitudinal groove bows slightly laterally and is very deep, with a V-shaped cross-section. The LTBA possesses four longitudinal tooth rows and the MTBA has three rows. The presence of more lateral longitudinal tooth rows than medial ones is consistent with the condition in *H. sanjuanensis* (MACN-Pv 18185, MCP-PV 1693), *H. mariensis* (UFRGS-PV 0149T, 0408T), *H. huxleyi* (ISIR 01), and the holotype of *H. tikiensis*^33^. By contrast, the MTBA has more longitudinal tooth rows than the lateral one in *H. gordoni*^39^, *Su. stockleyi* (SAM-PK-11705), and the unnamed hyperodapedontine from Nova Scotia^37^. The preserved L1 and M1 tooth crowns are strongly worn on the walls of the longitudinal groove, exposing the root in coronal section. The teeth of both tooth-bearing areas are relatively small and closely packed, as occurs in most hyperodapedontines with the exception of *I. genovefae*^35,45^. The preserved tooth crowns of both tooth-bearing areas have a circular cross-section, but it is not possible to determine the presence of pyramidal teeth because the posterior region of the tooth plate is not preserved. The preserved portion of the medial surface of the bone lacks lingual teeth, but it not possible to determine if they were present more posteriorly or anteriorly in the tooth plate. The fragment of dentary of CRILAR-Pv 582 possesses a V-shaped cross-section as a result of the presence of a transversely thin and sharp occlusal cutting blade. It is not possible to observe teeth in the dentary fragment.

The presence of a single longitudinal groove and more than two tooth rows in the MTBA of the maxillary tooth plate of CRILAR-Pv 582 allows referring this specimen to the *Hyperodapedon* clade^34^. Its maxilla differs from those of hyperodapedontines with a centrally located single longitudinal groove (i.e. *H. gordoni*, an hyperodapedontine from the Ischigualasto Formation, and the above *Hyperodapedon* sp. nov.), more tooth rows in the MTBA than in the LTBA (*Su. stockleyi*), less than two tooth rows in the LTBA (*I. genovefae*, the unnamed hyperodapedontines from Nova Scotia and Wyoming) or with two longitudinal grooves (*H. huenei* and *Te. sulcognathus*). Instead, the morphology of CRILAR-Pv 582 is congruent with that of *H. sanjuanensis*, *H. mariensis*, *H. huxleyi*, the holotype of *H. tikiensis*, and the unnamed Zimbabwean hyperodapedontine.

- *Teyumbaita* Montefeltro, Langer & Schultz, 2010^41^.
- *Teyumbaita* sp. nov.

#### Material.

**Figure 8:**
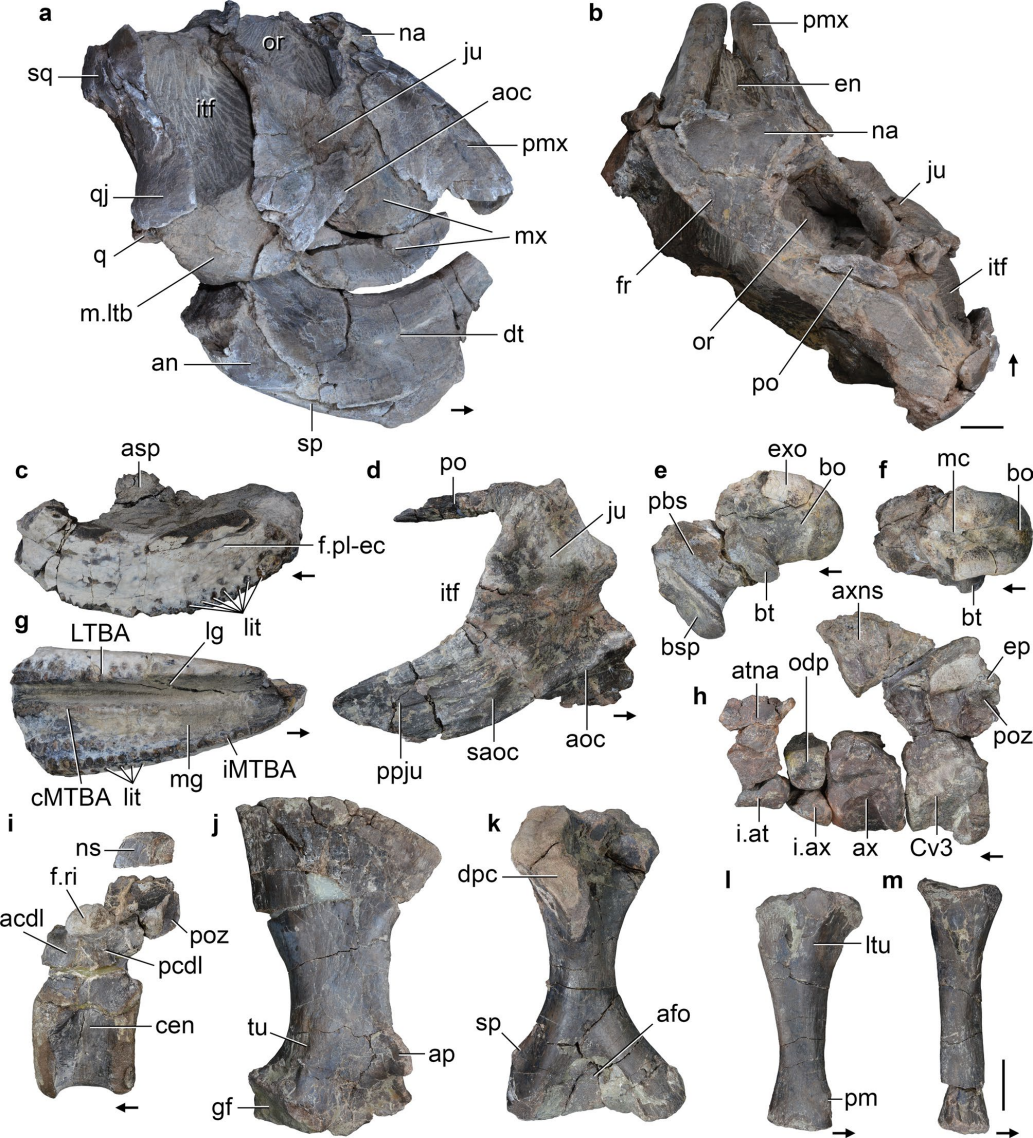
*Teyumbaita* sp. nov. Selected bones of specimens (**a**, **b**) CRILAR-Pv 595, (**c**, **e**, **g**–**i**) CRILAR-Pv 587, (**d**, **j**–**m**) CRILAR-Pv 651, and (**h**) CRILAR-Pv 586 collected in the outcrops of the Ischigualasto Formation immediately east to Cerro Las Lajas. (**a**, **b**) Skull, (**c**, **g**) right maxilla, (**d**) articulated right jugal and postorbital; (**e**, **f**) articulated exoccipitals, basioccipital and parabasisphenoid, (**h**) articulated atlas, axis and third cervical vertebra, (**i**) posterior dorsal vertebra, (**j**) right scapula, (**k**) right humerus, (**l**) right ulna, and (**m**) right radius in (**a**) right lateral, (**b**, **f**) dorsal, (**c**) medial, (**d**, **j**, **l**, **m**) lateral, (**e**, **h**, **i**) left lateral, and (**k**) anterior views. Arrows indicate anterior direction. Abbreviations: acdl, anterior centrodiapophyseal lamina; afo, anterior fossa; aoc, anguli oris crest; ap, acromial process; asp, ascending process; atna, atlantal neural arch; ax, axis; axns, axial neural spine; bo, basioccipital; bsp, basipterygoid process; bt, basal tuber; cen, centrum; cMTBA, central tooth bearing area; Cv3, third cervical vertebra; dpc, deltopectoral crest; ep, epipophysis; exo, exoccipital; f.ri, facet for rib; f.pl-ec, facet for palatine-ectopterygoid; gf, glenoid fossa; i.at, intercentrum of atlas; i.ax, intercentrum of axis; iMTBA, inner medial tooth bearing area; itf, infratemporal fenestra; ju, jugal; lg, lateral groove; lit, lingual teeth; LTBA, lateral tooth bearing area; ltu, lateral tuber; mc, median contact between exoccipitals; mg, medial groove; ns, neural spine; odp, odontoid process; pbs, parabasisphenoid; pcdl, posterior centrodiapophyseal lamina; pm, process for muscle attachment; po, postorbital; poz, postzygapophysis; ppju, posterior process of jugal; saoc, secondary anguli oris crest; sp, supinator process; tu, tuberosity. Scale bar: 2 cm.

CRILAR-Pv 586 (Fig. 8), partial cranium lacking most of the skull roof and right side, partial left hemimandible, a median segment of the right hemimandible, atlas, axis and third cervical vertebra, four middle-posterior cervical vertebrae, a fragment of humeral shaft, a probable metacarpal lacking the distal end, eight non-ungual phalanges, and indeterminate bone fragments (Fig. 8h). CRILAR-Pv 587, partial left premaxilla, right maxilla, left nasal, basicranium, right atlantal neural arch, five postaxial cervical vertebrae, a posterior dorsal vertebra, right scapula, coracoid, clavicle, humerus, ulna and femur, proximal and distal ends of fibula, a right metacarpal probably from digit II, two non-ungual and two ungual phalanges, and several ribs (Fig. 8c,e–g,i). CRILAR-Pv 588, partial left maxilla lacking its lateral edge and anterior tip, fragment of the medial dentary crest and indeterminate bone fragments. CRILAR-Pv 595, partial skull with almost complete right side and missing the left orbital and temporal regions, braincase and post-dentary bones with exception of the angulars (Fig. 8a, b). CRILAR-Pv 642, partial left maxilla lacking most of the ascending process, anterior tip, and occlusal surface of the LTBA, and—still in the field—partial postcranium. CRILAR-Pv 643, middle third of right maxilla, six postaxial centra, ventral end of clavicle, proximal and distal ends of tibia and indeterminate bone fragments of bones of a very small-sized individual. CRILAR-Pv 645, articulated right quadrate, squamosal and paroccipital process, probable partial left postfrontal, partial right parietal and pterygoid, partial parabasisphenoid, posterior two-thirds of left dentary, articulated posterior half of right surangular, prearticular and fragment of articular, six postaxial cervical vertebrae, two articulated probable anterior dorsal vertebrae, two dorsal or anterior caudal vertebrae, right scapula and humerus, partial scapular blade of another right scapula, multiple fragments of ribs and gastralia and several indeterminate bone fragments. This specimen consists of at least two individuals found in association with CRILAR-Pv 595. CRILAR-Pv 651, partial left premaxilla and dentary, posterolateral corner of right maxilla, partial right jugal, postorbital, quadrate, pterygoid and ectopterygoid, axial centrum, at least three postaxial centra, right scapula, humerus, ulna and radius, fragment of left scapula, distal third of metacarpal or metatarsal, three non-ungual phalanges, several rib fragments, and several probable cranial and postcranial indeterminate bone fragments (Fig. 8d,j–m).

#### Description.

The skull of *Teyumbaita* sp. nov. is broader than long, with a jugal representing the main component of its lateral surface, a fully closed lower temporal bar, and a dorsolaterally facing orbit (Fig. 8a,b), as occurs in other hyperodapedontines^41,44,45,50^. The nasals form a straight posterodorsal border in the single external naris, contrasting with the diagnostic notched border at the median line present in *Te. sulcognathus*^41^. The lateral surface of the main body of the jugal is deeply concave and lacks a secondary anguli oris crest (CRILAR-Pv 595, 651), contrasting with *I. genovefae*^45^ and the holotype and a referred specimen of *Te. sulcognathus*^41^. Nevertheless, the base of the posterior process of one specimen (CRILAR-Pv 651) has a thick, rugose ridge that does not extend further anteriorly on the bone (Fig. 8d: saoc). A very similar structure was interpreted as a posteriorly restricted secondary anguli oris crest in another referred specimen of *Te. sulcognathus*^41^. The presence of this latter ridge and the absence of a lateral depression at the base of the posterior process differs from the condition present in species of *Hyperodapedon* (e.g. *H. sanjuanensis*, MACN-Pv 18185; *H. huenei*^29^; *H. huxleyi*, ISIR 01; *H. gordoni*^39^; *H. mariensis*, UFRGS-PV 0149T). The anterior margin of the ascending process of the quadratojugal is slightly convex in lateral view, as occurs in *Te. sulcognathus*^41^, but contrasting with the concave margin of *H. huenei*^29^ and *Hyperodapedon* sp. nov. (CRILAR-Pv 585).

The maxillary tooth plate of the currently largest specimen of *Teyumbaita* sp. nov. has a transverse width of 47.6 mm (CRILAR-Pv 587), which is ca. 84% smaller than the largest specimen of *Te. sulcognathus* (transverse width = 56.7 mm, UFRGS-PV 290T). The maxillary tooth plate possesses two longitudinal grooves that define three tooth bearing areas (Fig. 8g), as occurs in *Te. sulcognathus*^41^, *H. huenei*^29^, the morphotype 2 of *H. tikiensis*^33^, and several non-hyperodapedontine rhynchosaurids^43,51^. The lateral groove is considered homologous to the single longitudinal sulcus of most hyperodapedontines (Chatterjee^52^ and subsequent authors). As a result, the MTBA is subdivided into a central medial tooth bearing area (cMTBA) and an inner medial tooth bearing area (iMTBA). The entire MTBA is broader than the LTBA, as is the case in *Te. sulcognathus* and *H. huenei*^29,41^, but it contrasts with the distinctly broader LTBA present in both morphotypes of *H. tikiensis*^33^. Both longitudinal grooves converge anteriorly at the anterior third of the maxilla of *Teyumbaita* sp. nov., resembling the condition in *Te. sulcognathus* and the morphotype 2 of *H. tikiensis*^33,41^, but the medial groove is restricted to the posterior third of the tooth plate in *H. huenei*^29^.

The number of tooth rows in the LTBA, cMTBA, and iMTBA show minor variation among preserved specimens. The LTBA and cMTBA have two longitudinal tooth rows posteriorly, and the iMTBA has two (CRILAR-Pv 587, 642, 643) or three to four (CRILAR-Pv 588) tooth rows on the posterior half of the tooth plate. The arrangement of these occlusal tooth rows resembles that of *Te. sulcognathus*^41^. In addition, there is a row of well-spaced lingual teeth immediately medial to the cushion-shaped iMTBA (Fig. 8c,g). This row of lingual teeth extends from the posterior end up to the posterior third (CRILAR-Pv 588, 642) or approximately the mid-length (CRILAR-Pv 587, 595) of the tooth plate and is composed of up to eleven teeth (CRILAR-Pv 587). By contrast, *Te. sulcognathus* possesses a single lingual tooth positioned on the posteromedial corner of the bone^41^. The lingual teeth of *Teyumbaita* sp. nov. are ventrally oriented, resembling the condition in *Hyperodaperdon* sp. nov. (CRILAR-Pv 585), but contrasting with *H. huenei*^29^ and the Zimbabwean hyperodapedontine^36^.

In the braincase, the ventral ends of the exoccipitals contact their counterparts on the floor of the endocranial cavity (Fig. 8e,f), as occurs in most other hyperodapedontines (see above), but contrasting with the absence of such contact in *Te. sulcognathus*^41^. The parabasisphenoid has an oblique, posterodorsally to anteroventrally oriented, main axis in lateral view. The basipterygoid processes are dorsoventrally taller than transversely broad, as in other hyperodapedontines with the exception of *I. genovefae*^45^.

The dentary has a lateral cutting blade and a lower and transversely thicker medial cutting blade, as occurs in *Te. sulcognathus* and non-hyperodapedontine rhynchosaurids^41^. Multiple lingual teeth are located on the top of the medial blade, immediately medial to it, and on a medially bulged border, being disposed in a crowded pattern, resembling the condition in *Te. sulcognathus*^41^, but differing from the well-spaced lingual teeth of *H. huenei*^29^ and several other hyperodapedontines (e.g. *H. mariensis*, MCN 1867-PV).

The morphology of the atlas (Fig. 8h) closely resembles that of *Te. sulcognathus*^46^ and other hyperodapedontines (e.g. *H. huxleyi*^32^). The dorsal margin of the neural spine of the axis possesses a strongly convex central portion in lateral view that becomes concave at the level of the postzygapophyses, resembling the condition in *H. gordoni*^39^. By contrast, the posterior portion of the dorsal margin of the axial neural spine of *Te. sulcognathus*^46^ (UFRGS-PV 0232T, 0298T) is convex in lateral view. The postaxial cervical vertebrae have a relatively short centrum with a thick, ventral keel. The postzygapophyses have a stout, crest-like epipophysis that vary in the series from short structures that do not extend posteriorly beyond the postzygapophyseal facet to substantially longer epipophyses, as occurs in *Te. sulcognathus*^46^. The best preserved dorsal vertebra has a spool-shaped centrum without a ventral keel (Fig. 8i). The neural arch possesses short and thick anterior and posterior centrodiapophyseal and postzygodiapophyseal laminae. There is no epipophysis, nor a hyposphene or hypantrum.

The scapula has a broad and fan-shaped blade, more anteriorly than posteriorly expanded (Fig. 8j). By contrast, the posterior margin of the scapular blade is nearly straight in *Te. sulcognathus*^46^. The acromial process is well-raised from the rest of the bone and mainly laterally projected, resembling the condition in some other hyperodapedontines (e.g. *H. huxleyi*, ISIR 01), whereas in *Te. sulcognathus* this process is shorter and blunt^46^. The humeral entepicondyle lacks the autapomorphic well-developed longitudinal groove of *Te. sulcognathus*^46^. The ectepicondyle has a tall supinator ridge and shallow ligament groove, which are mainly proximodistally oriented (Fig. 8k), as is the case in *Te. sulcognathus*^46^, *H. gordoni* (Benton 1983), and *Hyperodapedon* sp. nov. (CRILAR-Pv 585). By contrast, these ridge and groove are oblique, posteroproximally to anterodistally oriented, in *H. sanjuanensis* (MACN-Pv 18185) and *H. huxleyi*^32^. The ulna lacks an olecranon process and has a subtriangular lateral tuber in proximal view (Fig. 8l). The femur has a well-developed internal trochanter and a tibial condyle with a posteromedially oriented apex, as in some other hyperodapedontines (e.g. *Te. sulcognathus* and *I. genovefae*^45,46^).

#### Comments.

The new species of *Teyumbaita* from the Ischigualasto Formation is identified as a hyperodapedontine rhynchosaur because it bears the following synapomorphies of the clade^34^: orbit mostly dorsally oriented (CRILAR-Pv 595); orbit without an elevated rim along the jugal, postorbital, frontal, prefrontal and lacrimal (CRILAR-Pv 595, 651); fully closed lower temporal bar (CRILAR-Pv 586, 595, 651); anguli oris crest extended onto the anterior process of the jugal, but not the maxilla (CRILAR-Pv 595); maxilla well laterally overlapped by jugal (CRILAR-Pv 586, 595, 651); squamosal ventral process broader than over 50% of its dorsoventral length (CRILAR-Pv 595); squamosal ventral process overlapping the quadratojugal ascending process (CRILAR-Pv 595); dentary teeth conical and mesiodistally compressed (CRILAR-Pv 586, 595, 645); and posteriormost dentary teeth on the posterior half of the lower jaw (CRILAR-Pv 595). Besides, *Teyumbaita* sp. nov. has the following synapomorphies of the *Hyperodapedon* clade^34^: postorbital ventral process expanding dorsally to orbital height midpoint (CRILAR-Pv 595, 651); postorbital ventral process fits dorsal to the jugal (CRILAR-Pv 595, 651); maxillary tooth plate with more than two tooth rows in the MTBA (CRILAR-Pv 587, 588, 595, 642, 643); maxillary tooth plate with four or more tooth rows of occlusal teeth on its anterior half (CRILAR-Pv 587, 588, 595, 642); parabasisphenoid with a basipterygoid process wider than long (CRILAR-Pv 586, 587, 651); and postaxial cervical postzygapophyses with crest-shaped epipophysis (CRILAR-Pv 586, 587, 651). Among hyperodapedontines, *Teyumbaita* sp. nov. shares the following shynapomorphies with *Te. sulcognathus* and *H. huenei*^34^: jugal lateral surface with a secondary anguli oris crest dorsal to the primary anguli oris crest (CRILAR-Pv 651); frontal without groove on the dorsal surface (CRILAR-Pv 595); maxillary tooth plate with medial longitudinal groove (CRILAR-Pv 586–588, 595, 642, 643); maxillary area lateral to main groove narrower than the medial area (CRILAR-Pv 586–588, 642); medialmost row of occlusal teeth at posterior region of maxilla medially displaced and crowns without strictly occlusal direction (CRILAR-Pv 587, 588, 595, 642); and dentary medial surface at posterior portion forming a bulged area projecting medially from the remaining area of the dentary (CRILAR-Pv 586, 595, 645).

Within the *Te. sulcognathus*  + *H. huenei* clade, the new species from Ischigualasto shares with *Te. sulcognathus*, but not with *H. huenei*, the presence of a medial longitudinal groove extending beyond the mid-length of the maxillary tooth plate (CRILAR-Pv 587, 588, 595, 642) and dentary lingual teeth disposed in a crowded pattern (CRILAR-Pv 586, 588, 595, 645). The above discussed specimens from Cerro Las Lajas represent a new species of *Teyumbaita* because they differ from *Te. sulcognathus* in the presence of a straight posterodorsal border of the external naris (CRILAR-Pv 595), a row of lingual teeth on the maxilla (CRILAR-Pv 587, 588, 595, 642, 643), exoccipital contacting its counterpart on the floor of the endocranial cavity (CRILAR-Pv 586, 587), axis with a posterodorsally concave neural spine in lateral view (CRILAR-Pv 586), acromial process of the scapula thick and with an anterolateral apex (CRILAR-Pv 587, 651), well posteriorly expanded distal end of the scapular blade (CRILAR-Pv 587, 645, 651), and medial surface of the humeral entepicondyle without a longitudinal groove (CRILAR-Pv 587, 651).

- *Teyumbaita* sp.

#### Material.

CRILAR-Pv 589, posterior third of left maxilla. CRILAR-Pv 590, posterior end of left maxilla. CRILAR-Pv 591, posterior end of left maxilla. CRILAR-Pv 592, anterior end of maxilla. CRILAR-Pv 593, anterior third of right maxilla and a bone fragment. CRILAR-Pv 594, fragments of premaxilla and dentary, and indeterminate bone fragments. CRILAR-Pv 596, left premaxilla, fragment of right premaxilla, partial left dentary, three non-ungual phalanges, and multiple indeterminate fragments of cranial and postcranial bones. CRILAR-Pv 597, partial left premaxilla and dentary, both partial maxillae and bone fragments. CRILAR-Pv 598, partial skull, including maxillae and dentaries, atlantal intercentrum, cervical and dorsal vertebrae, scapula, coracoid and forelimb, and indeterminate bone fragments (most of the specimen is still unprepared).

#### Description and comments.

Most of the specimens (CRILAR-Pv 589–593, 597, 598) here referred to *Teyumbaita* sp. are represented by partial, generally isolated, maxillae with two longitudinal grooves that extend anteriorly beyond the posterior third of the tooth plate and, thus, can be referred to this genus^41^. However, the presence of the row of lingual teeth that is diagnostic of *Teyumbaita* sp. nov. cannot be determined in these specimens because of damage or lack of preservation. Similarly, other specimens (CRILAR-Pv 594, 596) that do not preserve a maxilla are referred to the genus *Teyumbaita* because of the presence of lateral and medial cutting blades in the dentary and more than two longitudinal rows of dentary lingual teeth that are disposed in a crowded pattern^41^. By contrast, the dentary lingual teeth of *H. huenei* are less numerous and well-spaced from one another^29^. The morphology of these specimens is consistent with those of both *T. sucolgnathus* and *Teyumbaita* sp. nov., but there is no character-state that allow determining them at an alpha-taxonomy level.

- Archosauriformes Gauthier et al., 1988^53^ sensu Gauthier et al.^53^.
- Proterochampsidae Sill, 1967^54^ sensu Trotteyn^55^.
- *Proterochampsa* Reig, 1959^56^.
- *Proterochampsa barrionuevoi* Reig, 1959^56^.

#### Material.

CRILAR-Pv 579, a fairly complete right maxilla articulated to the anterior tip of jugal, a partial left maxilla, an anterior end of left dentary, an anterior-middle cervical centrum, a posterior cervical or anterior dorsal centrum, a dorsal centrum, a sacral centrum, fragments of at least two other vertebral centra, a posterior cervical neural arch, proximal portions of two cervical ribs, a partial femoral shaft, and indeterminate postcranial bones (Fig. 10).

#### Description.

CRILAR-Pv 579 has an excellent preservation, showing fine details of the bone, such as the neurovascular foramina on the inner surface of the maxilla. The external ornamentation consists of nodular process on the maxilla and small pits and ridges of variable size on the dentary. In contrast, the maxillae of *Chanaresuchus bonapartei*, *Gualosuchus reigi*, *Tropidosuchus romeri*, and *Pseudochampsa ischigualastensis* are not ornamented^57^.

**Figure 9:**
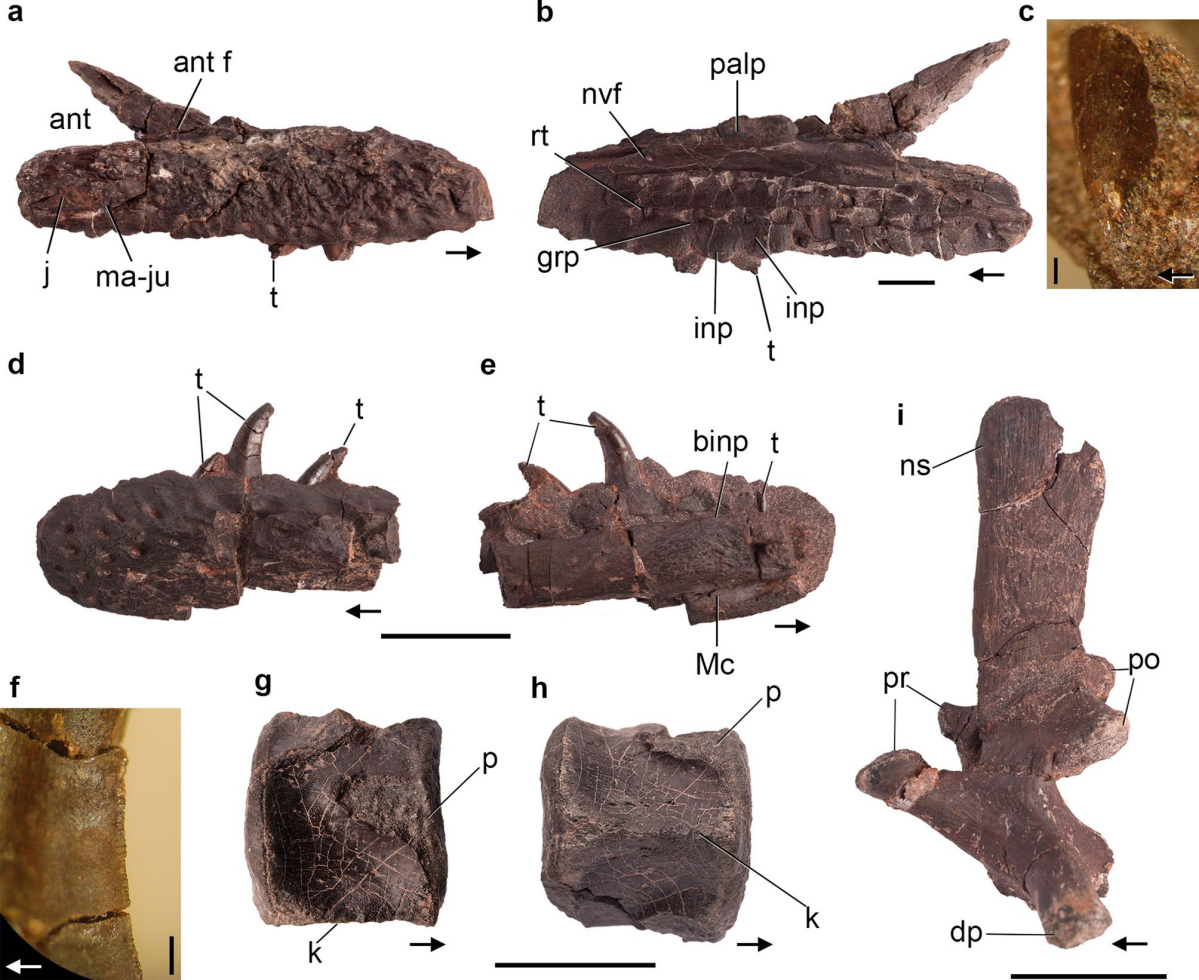
*Proterochampsa barrionuevoi* CRILAR-Pv 579. (**a**–**c**) Right maxilla and close-up of distal serrations, (**d**–**f**) left dentary and close-up of distal denticles, (**g**, **h**) anterior-middle cervical centrum, and (**i**) cervical neural arch in (**a**, **d**) lateral, (**b**, **e**) medial, (**c**, **f**) labial, (**g, i**) left lateral, and (**h**) ventral views. Arrows indicate anterior direction. Abbreviations: ant, antorbital fenestra; ant f, antorbital fossa; binp, base of interdental plate; dp, diapophysis; grp, groove of resorption pits; inp, interdental plates; j, jugal; k, ventral keel; ma-ju, maxilla-jugal suture; Mc, Meckelian canal; ns, neural spine; p, parapophysis; palp, palatal process; po, postzygapophyses; pr, prezygapophyses; rt, replacement tooth; t, tooth. Scale bars: (**a**, **b**, **d**, **e**, **g**–**i**) 2 cm, and (**c**, **f**) 1 mm.

The right maxilla of CRILAR-Pv 579 is fairly complete, with a damaged anterior end, and preserved in articulation with the anterior process of the jugal. The anterior process of the maxilla extends a long way anteriorly to the antorbital fenestra, has a subhorizontal dorsal slope, and is strongly ornamented on its external surface (Fig. 9a). The premaxillary contact, at the anterior margin of the process, is not preserved. The ascending process forms the anterodorsal margin of the antorbital fenestra. The transition between the anterior and ascending processes is gradual, forming an obtuse angle, similar to the condition of *Ch. bonapartei* and *Ps*. *ischigualastensis*. As in *Proterochampsa nodosa* and *Pro*. *barrionuevoi*, the antorbital fossa excavates only the ascending process of the maxilla, where it forms a broad, anteriorly rounded depression. The palatal process is dorsoventrally low, forming the lateral and anterior borders of the internal nares (Fig. 9b). The ventral surface of this process bears some, possibly neurovascular, small foramina, as well as some striated areas for probably soft tissue attachment. Its dorsal surface is smooth, and slightly convex. The posterior process of the maxilla is laterally overlapped and excluded from most of the external ventral border of the antorbital fenestra by the jugal (Fig. 9a). In contrast, the maxilla forms most of the ventral border of the antorbital fenestra in other proterochampsids. The antorbital fenestra is mainly dorsally oriented, unlike the laterally facing fenestra of *Tro. romeri*, *Cerritosaurus binsfeldi* and rhadinosuchines. Ten tooth positions are preserved in the maxilla, but the total number would have been slightly higher, resembling the tooth counts of other specimens of *Proterochampsa* (e.g., at least 11 tooth positions in PVSJ 77^58^). The preserved maxillary tooth crowns are relatively short and distally recurved (Fig. 9a,b). They have denticles on the apical three-quarters of the distal margin of the crown, whereas the mesial margin is not serrated. The denticles have a quadrangular edge and the interdenticular spaces do not extend onto the central region of the crown as blood grooves. There are 5 denticles per mm and they are apically oriented (Fig. 9c), contrasting with the orthogonal orientation of the maxillary denticles of other proterochampsids and the widespread condition in other archosauriforms. The apex of an erupting, mesial replacement tooth is visible between the bases of two interdental plates. The medial wall of the alveoli is formed by pentagonal interdental plates that do not contact one another (Fig. 9b). This exposes a wide groove extending along the bases of the interdental plates, with a replacement pit for each tooth^27,59^ (Fig. 9b).

The preserved anterior tip of jugal possesses an ornamented external surface similar to that on the maxilla (Fig. 9a). The bone reaches anteriorly the level of the anterior border of the antorbital fenestra, as occurs in other specimens of *Pro. barrionuevoi*^58^.

The anterior end of dentary (Fig. 9d,e) lacks most of its ventral region. In medial view, the preserved ventromedial region includes a small part of the roof of the Meckelian canal, showing that the latter was ventrally placed. In lateral view, the dentary surface is strongly ornamented by several pits and small ridges, unlike the smooth surface of that bone present in *Ps. ischigualastensis*, *Tro. romeri*, and *Ch. bonapartei*. The preserved portion of dentary bears five alveoli, which are oval and anteroposteriorly longer than broad, and three teeth in situ with different degrees of eruption. The dentary tooth crowns have a serrated distal carina, whereas the mesial one lacks serrations, as occurs in the maxillary teeth. There are 6 denticles per mm in the dentary teeth. Their morphology is similar to those of the maxillary teeth, but they are orthogonal to the main axis of the crown (Fig. 9f).

Four vertebral centra were recovered, the best preserved of which belongs to an anterior or mid-cervical vertebra because its ventral surface bears a well developed longitudinal keel and the parapophyses are located slightly above the mid-height and adjacent to the anterior margin of the centrum (Fig. 9g, h). The ventral keel extends along the entire length of the centrum, as also seen in cervical vertebrae of *Ps. ischigualastensis* and *Ch. bonapartei*. The centrum is approximately as tall as long in lateral view (Fig. 9g), differing from the longer than tall cervical vertebrae of *Ps. ischigualastensis*. The anterior and posterior articular facets are shallowly concave and suboval, being lateromedially broader than tall. The base of the parapophysis is sub-oval, longer than tall.

The lateral surface of all the centra lacks a lateral fossa. The disarticulated cervical neural arch (Fig. 9i) lacks laminae and the neural spine is very tall, slightly anteroposteriorly expanded at its distal end, and vertical in lateral view. The distal end of the neural spine has longitudinal striations and lacks a spine table, as is the case in other proterochampsids^58^. The spine has a rounded anterodorsal corner, whereas the posterodorsal one is broken. The right prezygapophysis is complete, with a transversely broad and dorsomedially facing articular facet. The postzygapophyseal articular surfaces are ventrolaterally facing. The preserved right diapophysis is entirely located on the neural arch and at level with the roof of the neural canal. The diapophysis is long and slightly posteriorly oriented in dorsal view.

A small section of long-bone diaphysis is preserved. It has a smooth surface, an oval cross section, and may correspond to a femur because of the presence of a large, drop-shaped nutrient foramen.

#### Comments.

Proterochampsidae is a clade of archosauriform reptiles known exclusively from the Late Triassic of Argentina and Brazil. *Proterochampsa* includes two species: *Pro*. *barrionuevoi* from the Ischigualasto Formation, Argentina^54,56,60^, and *Pro. nodosa* from the *Hyperodapedon* Assemblage-Zone, Santa Maria Formation, Brazil^61^. Previous to this report, *Pro*. *barrionuevoi* was known only from the upper La Peña, Cancha de Bochas, and lower Valle de la Luna members of the Ischigualasto Formation in the Ischigualasto Provincial Park, San Juan Province^3^.

CRILAR-Pv 579 is assigned to *Proterochampsa* based on the following synapomorphies of the genus^58^: dermal sculpturing consisting of nodular protuberances and prominent ridges with smaller periodic nodular growths along their length, antorbital fossa restricted to an elongate depression on the maxilla anteriorly to the antorbital fenestra; dorsoventrally flattened skull with dorsally facing antorbital fenestrae. The specimen from the Hoyada del Cerro Las Lajas can be referred to *Pro*. *barrionuevoi* because it possesses a low, but coarse ornamentation on the external surface of the maxilla and jugal, whereas *Pro*. *nodosa* has fewer and larger protuberances on the dermal bones^58^.

- Archosauria Cope, 1869^62^ sensu Gauthier & Padian^63^.
- Pseudosuchia Zittel, 1887−1890^64^ sensu Gauthier & Padian^63^.
- Suchia Krebs, 1974^65^.
- Paracrocodylomorpha Parrish, 1993^66^.

#### Material.

**Figure 10:**
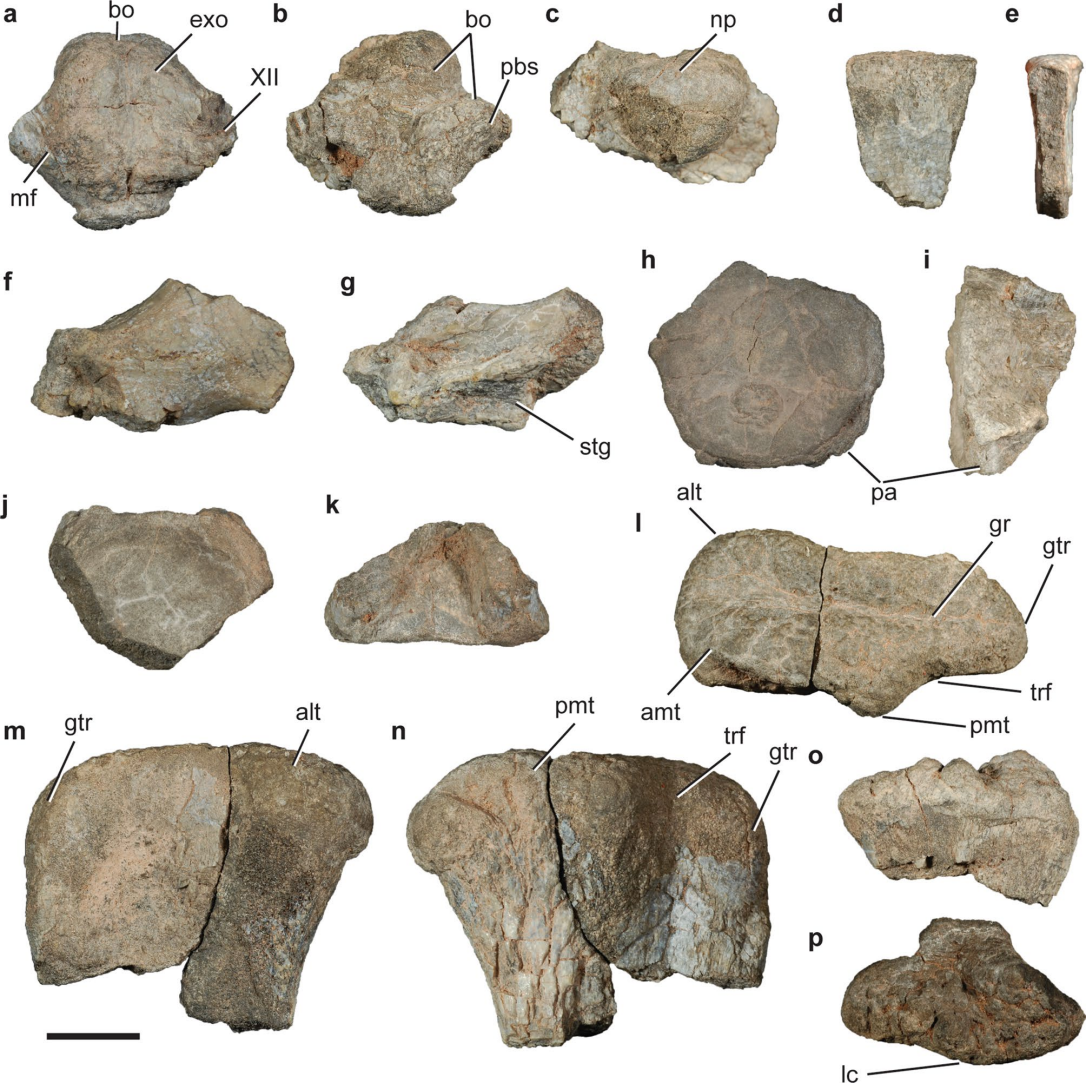
Paracrocodylomorpha indet. CRILAR-Pv 581. (**a–c**) Fragmentary braincase, (**d**, **e**) neural spine, (**f**, **g**) right paroccipital process, (**h**, **i**) fragmentary cervical vertebra, (**j**, **k**) fragmentary vertebra, (**l**–**n**) right femur, and (**o**, **p**) right tibia in (**a**, **k**) dorsal, (**b**, **g**) ventral, (**c**, **n**) posterior, (**d**, **i**, **o**) lateral, (**e**) cross-section, (**f**) posterodorsal, (**h**, **m**) anterior, (**j**) anterior/posterior, and (**l**, **p**) proximal views. Abbreviations: alt, anterolateral tuber; amt, anteromedial tuber; bo, basioccipital; exo, exoccipital; gr, groove; grt, greater trochanter; lc, lateral condyle; np, notochordal pit; pa, parapophysis; pbs, parabasisphenoid; pmt, posteromedial tuber; stg, stapedial groove; trf, intertrochanteric fossa. Scale bar: 1 cm.

CRILAR-Pv 581, articulated basioccipital, ventral end of exoccipitals and parabasisphenoid, left paraoccipital process, five fragmentary vertebral centra, two neural spine fragments, proximal end of right femur, and proximal end of right tibia (Fig. 10).

#### Description.

The occipital condyle is wider than high, slightly bilobed, and has a subcircular notochordal pit on the dorsal margin of its occipital surface (Fig. 10a–c). The latter condition is also present in rhynchosaurs (*Hyperodapedon* sp., GPIT/RE/09221), erpetosuchids (*Tarjadia ruthae*, CRILAR-Pv 478; *Archeopelta arborensis*, CPEZ 239a), and the loricatans *Saurosuchus galilei* (PVSJ 32) and *Batrachotomus kupferzellensis* (SMNS 80260), differing from proterochampsids (*Proterochampsa barrionuevoi*, PVSJ 77; *Chanaresuchus bonapartei*, PULR 07) and doswelliids (*Doswellia kaltenbachi*, USNM 214823; *Jaxtasuchus salomoni*, SMNS 91083), in which the notochordal scar consists of a deeper and larger pit or vertical furrow^27^. The basioccipital has an extremely short, almost absent occipital neck, like in erpetosuchids (*Ta. ruthae*, CRILAR-Pv 478; *Arc. arborensis*: CPEZ 239a) and doswelliids (*J. salomoni*, SMNS 91352; *Do. kaltenbachi*, USNM 214823). The basal tubera of the basioccipital are anteroposteriorly short and lateroventrally projected, resembling the blade-like tubera present in loricatans (*B. kupferzellensis*: SMNS 80260; *Postosuchus kirkpatricki*: TTUP 9002; *Sa. galilei*: PVSJ 32; *Dibothrosuchus elaphros*^67^; *Caiman yacare*: MLP 604), but not in poposauroids (*Shuvosaurus inexpectatus*: TTUP 9280, *Arizonasaurus babbitti*: MSM 4590, *Effigia okeeffeae*^68^) or aetosaurs (*Neoaetosauroides engaeus*: PVL 5698; *Stagonolepis robertsoni*: NHMUK PV R4784, *Desmatosuchus spurensis*: TTUP 9024). As in the shuvosaurids *Ef. okeeffeae*^68^ and *Sh. inexpectatus* (TTUP 9280), the ventral end of the exoccipitals do not contact at the midline, differing from most non-crocodylomorph pseudosuchians (e.g., *B. kupferzellensis*, *Ari. babbitti*, *St. robertsoni*, *N. engaeus*, *Ta. ruthae*, *Arc. arborensis*), in which the exoccipitals contact at the midline. The exoccipitals barely contribute to the occipital condyle and where it is broken off, we can recognize at least one exit for cranial nerve XII (Fig. 10b). The posteriormost region of the parabasisphenoid is preserved, contributing to the basal tubera. In its anterolateral contact with the right exoccipital, at the base of the braincase cavity, a small foramen can be recognized and putatively referred to part of the wall of the metotic foramen. This condition is seen in other pseudosuchians such as *Ari. babbitti*^69^, *Ef. okeeffeae*^68^, and *B. kupferzellensis*^70^. The base of the right paroccipital process of the opisthotic is partially preserved (Fig. 10f,g). It is subtriangular in cross-section at the base and oval towards its distal end, being anteroventrally to posterodorsally flattened. The paroccipital process has a well excavated stapedial groove on its posteroventral surface that opens into the brain cavity through the metotic foramen (Fig. 10g).

The vertebral remains of CRILAR-Pv 581 are very fragmentary (Fig. 10d, e,h–k), mainly represented by articular surfaces of centra, which have circular profiles and are markedly concave . The most complete ones are hourglass-shaped in ventral view, strongly constricted towards the body of the vertebra, and the parapophysis can be identified on the ventrolateral margin of one of them. Strongly constricted centra can be seen in cervical vertebrae of *Sillosuchus longicervix* (PVSJ 85, PVL 2267), *Sh. inexpectatus* (TTUP 09001), and *Ef. okeeffeae*^71^ among poposauroids and an unnamed early crocodylomorph from the Ischigualasto Formation in the Hoyada de Ischigualasto (PVSJ 846, 890^72^). Two fragmentary neural spines are preserved (Fig. 10d,e). They are laterally compressed and do not expand distally; the dorsal margin is rounded, straight in lateral view, and has transverse striations.

The proximal end of the right femur (Fig. 10l–n) is anterolaterally to posteromedially compressed resembling that of *Sh. inexpectatus* (TTUP 3870), *Ef. okeefeae*^68^, *Ari. babbitti*^73^, and *Poposaurus gracilis* (TTUP 11613). Its proximal surface is well preserved, showing a longitudinal straight groove (Fig. 10l) as seen in several pseudosuchians (e.g. *Pre. chiniquensis*, SNSB-BSPG AS XXV 10; *Pop. gracilis*, TMM 31100–408, UCMP 28359; *Sil. longicervix*, PVSJ 85; *Aetosauroides scagliai*, PVL 2073). As typical of pseudosuchians, the proximal head is rounded and not clearly differentiated from the shaft, unlike those of non-aphanosaurian avemetatarsalians, in which the head is separated from the shaft by a notch or a concave depression ventral to the femoral head^74^. A moderately developed greater trochanter can be recognized on the posterolateral region of the proximal end of the femur, granting it a quadrangular shape in posterior view (Fig. 10m,n). The posteromedial tuber^71^ is the largest of the proximal tubers and is subtriangular in proximal view, contrasting with the anteromedial tuber, which is smaller and more rounded, a condition similar to that of *Poposaurus gracilis* (TMM 31100–408, UCMP 28359). The anterolateral tuber is rounded and wide, occupying the medial third of the anterior margin of the femoral head. The intertrochanteric fossa is seen between the greater trochanter and the posteromedial tuber, it is shallow and level with the greater trochanter. The shaft is strongly anterolaterally to posteromedially compressed with the cortical bone collapsed where the bone is broken off.

The proximal end of the right tibia is partially preserved and the proximal surface is convex (Fig. 10o,p). The anterior margin of the tibia is rounded whereas the posterior one is sharper. The lateral condyle is well-developed and offsets anteriorly from the medial condyle as in several other archosauriforms (e.g., *Ch. bonapartei*, PVL 4575). The medial surface of the proximal end of the tibia is slightly concave anteriorly and convex posteriorly.

#### Comments.

CRILAR-Pv 599 can be referred to Paracrocodylomorpha, in particular the clade composed of *Saurosuchus galilei* + Crocodylomorpha, based on the following synapomorphy^81^: anteroposteriorly shortened (blade-like) basal tubera of the basioccipital. Within this group it resembles Crocodylomorpha in the absence of contact between the ventral margin of the exoccipitals. Nevertheless, this condition is also present in shuvosaurid poposauroids (*Ef. okkeeffeae* and *Sh. inexpectatus*)^71^. In this regard, there are other character states of CRILAR-Pv 599 that resembles poposauroids: vertebrae with very constricted (hourglass-shaped) centra; unexpanded distal end of neural spines; anterolateral to posteromedially compressed proximal end of femur; straight longitudinal furrow on the proximal surface of femur. By contrast, it differs from shuvosaurid poposauroids in the presence of a rounded posteromedial tuber, lower than the anteromedial one, on the proximal end of the femur. As a result, the morphology of CRILAR-Pv 599 does not completely match that of poposauroids but we refrain to unambiguously refer it to Crocodylomorpha because of its fragmentary condition and similarities with *Po. gracilis*. Another paracrocodylomorph is known from the Hoyada del Cerro Las Lajas, the crocodylomorph *Trialestes romeri* (PVL 3889)^75^, and although it has a congruent overlapping morphology with the vertebrae of CRILAR-Pv 599, there are no preserved diagnostic features to determine if this specimen belongs to the same species.

- Aetosauria Marsh, 1884^76^ sensu Parker, 2007^77^.
- *Aetosauroides* Casamiquela, 1960^78^.
- *Aetosauroides scagliai* Casamiquela, 1960^78^.

#### Material.

**Figure 11:**
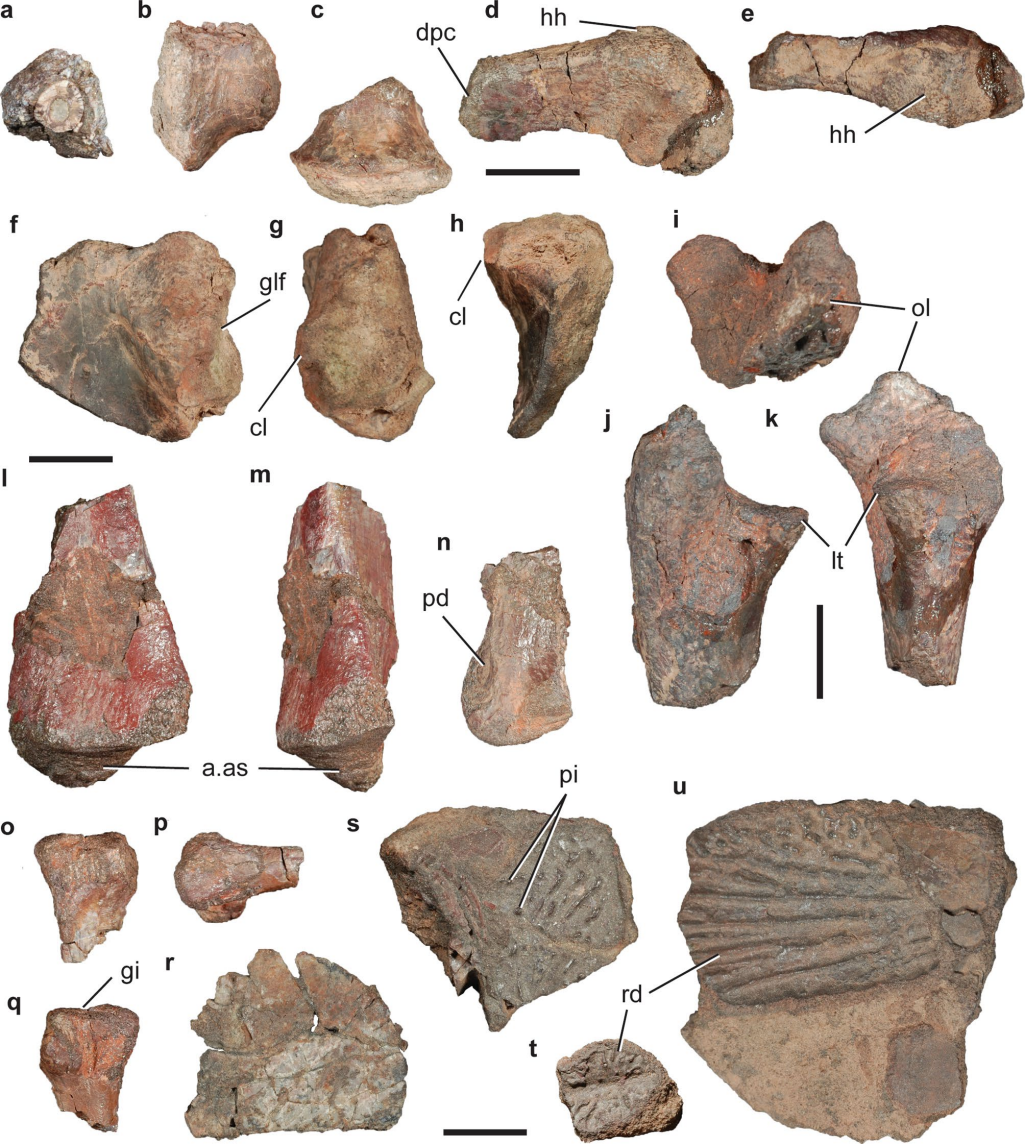
*Aetosauroides scagliai* CRILAR-Pv 580. (**a**) Tooth in cross section. Fragmentary vertebra in (**b**) lateral and (**c**) ventral views. Proximal end of right humerus in (**d**) anterior and (**e**) proximal views. Fragmentary right coracoid in (**f**) lateral, (**g**) posterior, and (**h**) ventral views. Proximal end of ulna in (**i**) proximal, (**j**) anterior, and (**k**) lateral views. Distal end of left tibia in (**l**) lateral and (**m**) anterior views. (**n**) Distal end of left fibula in lateral view. Distal end of metatarsal in (**o**) dorsal, (**p**) lateral, and (**q**) ventral views. (**r**) Incomplete paramedian osteoderm in external view. Moulds of external surface of (**s**) paramedian, (**t**) appendicular, and (**u**) ventral osteoderms. Abbreviations: a.as, articular surface for the astragalus; cl, coracoid lip; dp, deltopectoral crest; glf, glenoid fossa; hh, humerus head; lt, lateral tuber; np, notochordal pit; ol, olecranon process; pd, posterior depression; pi, pits; rd, ridges. Scale bars: 1 cm.

CRILAR-Pv 580, several fragmentary teeth, two fragmentary vertebral centra, numerous fragments and natural casts of paramedian, ventral, and appendicular osteoderms, fragmentary ribs, right fragmentary coracoid, proximal end of right humerus, proximal end of both ulnae, distal end of left tibia, and distal end of metatarsal (Fig. 11).

#### Description.

Several teeth are preserved as fragments and natural casts. Preserved tooth crowns lack their apices and are circular in cross-section (Fig. 11a), being clear that they were conical, resembling those of *A. scagliai* (PVL 2052, 2059) and *N. engaeus* (PVL 3528), and contrasting with the leaf-shaped teeth of *S. olenkae* (ZPAL AbIII/1995) and *De. spurensis* (TTUP 09420). Because of their poor preservation, it cannot be determined if they had wear facets or serrations.

Both recovered vertebrae are represented by poorly preserved partial centra, which are spool-shaped (Fig. 11b,c), as typical of aetosaurs. The articular surfaces are circular, but it cannot be determined whether they represent anterior or posterior facets. The several incomplete ribs are elliptic in cross-section and some have a sharp edge on the anterolateral or posterolateral margin.

The partial right coracoid is represented by the glenoid fossa (with a medially expanded coracoid lip) and the posteroventral portion of the bone (Fig. 11f–h). The glenoid fossa is teardrop-shaped, with the tapering portion towards the scapula. The subglenoid lip is damaged and it cannot be determined whether it had a postglenoid process or not. The proximal fragment of the right humerus preserves a globose head and the proximal part of the deltopectoral crest (Fig. 11d,e). The proximal end of the humerus is anteroposteriorly compressed, corresponding to a gracile element that resembles that of *A. scagliai* (PVL 2073) and contrasts with the thick humeral head of *N. engaeus* (PVL 3525). Both preserved ulnae have a clearly discernible tapering, but relatively short olecranon process at the proximal end (Fig. 11i–k). The olecranon process is shorter than the long axis of the proximal end of the ulna as in other suchians (e.g., *Riojasuchus tenuisceps*: PVL 3828; *A. scagliai*: PVL 2073; *Fasolasuchus tenax*: PVL 3850; *P. kirkpatricki*: TTUP 9000). Particularly, the olecranon of CRILAR-Pv 580 is lateromedially compressed and sharp, resembling that of *A. scagliai* (PVL 2073) and *Typothorax coccinarum* (NMMNH-P 56299), and contrasting with that of most aetosaurs, which are wider and more rounded (*St. olenkae*: ZPAL AbIII/1179; *Aetosaurus ferratus*: SMNS 5770 S16; *N. engaeus*: PVL 3525). A lateral tuber for the articulation of the radius is present on the proximal end of the ulna of CRILAR-Pv 580, as in other suchians^71^.

The distal end of the left tibia is elliptic in cross-section with the anterior margin slightly tapering. The distal articular surface would have contacted the astragalus and its posterolateral corner projects further ventrally (Fig. 11l,m). This asymmetric distal end with a ventral projection is also present in *A. scagliai* (PVL 2052), *De. spurensis* (UCMP 25877, 25886), *Ri. tenuisceps* (PVL 3827), *Pos. kirkpatricki* (TTUP 09002), *Pop. gracilis* (UCMP 25804), and *Revueltosaurus callenderi* (PEFO 34273, 34561), differing from the more symmetric distal end of the tibia of *B. kupferzellensis* (SMNS 52970, 54840), *Prestosuchus chiniquensis* (CPEZ 239b), *St. olenkae* (ZPAL AbIII/1178), *N. engaeus* (PVL 3525), and *Ty. coccinarum* (NMMNH-P 36075, 56299). Despite its fragmentary nature, the posterior half of the distal end of the left fibula can be recognized. It has a proximodistally elongated depression on the posterior surface, delimited laterally by a sharp edge (Fig. 11n). The distal articular surface is convex and circular in cross-section. A distal fragment of metatarsal is preserved and possesses a ginglymoid articulation (Fig. 11o–q). Two condyles occupy the ventral surface, with the lateral one more ventrally projected than the medial one, resembling the right metatarsal I of *A. scagliai* (PVL 2052). The cross-section of the shaft is elliptic, being dorsoventrally compressed.

Numerous osteoderms are preserved in association, mainly as natural moulds. Most represent paramedian osteoderms (wider than long), there are a few ventral elements (quadrangular), and two are appendicular osteoderms (rhomboidal) (Fig. 11r–u). Their external ornamentation pattern is composed of long ridges and deep grooves that radiate from the dorsal eminence. There are some pits near this dorsal eminence as well. The characteristic anterior bar without ornamentation can be identified in some casts of paramedian osteoderms. Their internal surface is flat and unornamented. The appendicular osteoderms present a longitudinal eminence that projects from the anterior to the posterior margin and lack an anterior bar.

#### Comments.

CRILAR-Pv 580 is here assigned to *A. scagliai* (by monotypy) based on the morphology of the teeth, the presence of a tapering olecranon on the ulna, a well-developed ventral projection on the distal end of tibia, and the general morphology of the osteoderms.

## Discussion

### Ischigualasto chronostratigraphic framework

Our measured succession of the Ischigualasto Formation at the Hoyada del Cerro Las Lajas is 1059 m-thick (92% exposed), compared to a maximum formation thickness of 691 m in the central IPP^79^. Our Bayesian age-stratigraphic model based on three new high-precision U–Pb CA-ID-TIMS tuff dates from the Hoyada del Cerro Las Lajas can be extended to the Ischigualasto Formation and its rich fossil record at IPP based on the following considerations: (a) the dated ‘Toba-2’ marker tuff bed, 107 mab at Hoyada del Cerro Las Lajas, is a direct correlative of the Herr Toba bentonite, ~ 20 m above base of the formation at IPP and, (b) the relatively sharp, but conformable contact of the Ischigualasto Formation with the overlying Los Colorados Formation is expected to be the same age at both location (see *stratigraphy* in Supplementary Information). Therefore, we place conservative age constraints of 230.2 ± 1.9 Ma and 221.4 ± 1.2 Ma, respectively, on the base and top of the Ischigualasto Formation throughout the Ischigualasto-Villa Unión Basin.

Previously reported ages from the Ischigualasto Formation at IPP were based on ^40^Ar/^39^Ar geochronology of various vintages, with tuffaceous feldspar Ar analyses spanning nearly two decades of research. The legacy ^40^Ar/^39^Ar plateau age (sanidine; incremental laser heating method) of the Herr Toba bentonite marker from the basal La Peña Member of the formation^8^ was 227.78 ± 0.30 Ma (1σ analytical error only). Subsequent revisions to the ^40^K decay constants and age of the fluence monitor standard used in ^40^Ar/^39^Ar geochronology necessitated recalculation of the latter date, resulting in revised ages of 229.2 Ma^80^, 231.4 ± 0.30 Ma^2^ and 230.8 ± 4.5 Ma^81^, the latter including fully propagated uncertainties (1σ) from the decay constants of ^40^K. This historical age and its subsequent revisions are superseded by our new U–Pb zircon age of 229.25 ± 0.30 Ma (fully propagated 2σ uncertainty) for the correlative of the Herr Toba bentonite at Cerro Las Lajas.

^40^Ar/^39^Ar geochronology from the upper Valle de la Luna Member of the Ischigualasto Formation at IPP has been controversial due to complex age spectra of the analysed feldspars (both K-feldspar and plagioclase) that have complicated objective pooling of the data for mean age calculation. Shipman^82^ reported in an unpublished thesis a weighted mean ^40^Ar/^39^Ar age of 217.1 ± 3.0 Ma (2σ) with a MSWD of 14.03 from a sample ~ 70 m below the top of the formation^79^; although the only K-feldpar analysed from this sample yielded an age of 221.9 ± 2.2 Ma. Another ^40^Ar/^39^Ar age of 225.9 ± 0.9 Ma (1σ) from a combination of plagioclase and K-feldspar analyses was reported^2^ from the same stratigraphic level. Finally, the magnetostratigraphic record of the Los Colorados Formation at IPP has been interpreted to yield an age of 227 Ma^83^ for the base of the formation (i.e., top of the Ischigualasto Formation). Superseding the previous ^40^Ar/^39^Ar geochronology, our Bayesian age-stratigraphic model based on U–Pb CA-ID-TIMS geochronology yields an age of 221.4 ± 1.2 Ma for the uppermost Ischigualasto Formation. This age is in conflict with the ^40^Ar/^39^Ar geochronology of Martínez et al.^2^ and indicates that the magnetostratigraphic age estimate of Kent et al.^83^ is inaccurate by at least 5 Myr. This also casts doubt about this age estimate of 214 Ma for the uppermost Los Colorados Formation and its fauna^83^, which could be younger based on our results.

Based on our new U–Pb geochronology, the 1059 m-thick succession of the Ischigualasto Formation at the Hoyada del Cerro Las Lajas was deposited in 8.8 (± 1.9) Myr, which translates into an average sediment accumulation rate of ~ 120 m/Myr. Compared to the most expanded part of the formation at IPP (691 m) with an average accumulation rate of ~ 74 m/Myr, the Cerro Las Lajas succession is ~ 1.6 times more expanded.

### Taphonomical model

The Ischigualasto Formation was deposited under a semi-arid palaeoclimate, and the presence of floodplains along with the development of palaeosoils at the mid-section, indicates periodic rainfall^84^. The attributes observed in the vertebrate remains (large concentration of pristine, articulated, and non-weathered fossils, autochthonous assemblages, general absence of scavenging, little or no reworking) at the base of the Ischigualasto Formation indicates a specific taphonomic model; i.e., short transport and exposure (= “census assemblage” according to the Johnson’s Model I), with rapid burial and low time-averaging, resulting in three-dimensionally arranged skeletal remains, with no sorting or orientation, and a polytypic taxonomic content^85^. Rapid burial, allied with various biological processes (e.g., faunistic turnovers, local extinctions), directly and positively influence fossil preservation. These biological and physical factors would haved a direct effect on bone input rates and time-averaging. Accordingly, the lower levels (first 300 m) of the Ischigualasto Formation have the highest concentration and the best preservartion of fossil vertebrates^3, 86^.

Based on the above model, the palaeoecosystem of the Ischigualasto Formation as recorded in the Hoyada del Cerro Las Lajas was initially characterized by a high biocenosic (life assemblages) load, represented by an abundant vertebrate association, similar to that seen in outcrops of the IPP, in San Juan Province^3^. Due to several death factors (e.g., torrential storms, increased volcanism, and biotic factors), a poor thanatocenosis phase (pre-burial death assemblage) occurred, and rapid burial factors quickly introduced the remains into the taphocenosis phase.

The middle and top levels of the Ischigualasto Formation in the Hoyada del Cerro Las Lajas have a meagre fossil record or are almost devoid of fossils, as also observed in the IPP^3^. The reasons behind this pattern are not clear. Pyroclastic deposits, such as bentonites, ignimbrites, and welded tuffs, indicate a high volcanic influence in the area and elsewhere^87–90^. These materials are much more abundant in the upper third of the studied succession, reaching significant thicknesses in certain intervals. These volcanogenic deposits suggest proximity to volcanic centers that may have resulted in more hostile palaeoenvironments, with a reduced biomass. Yet, this is a highly conjectural inference, given that the biocenosis and thanatocenosis phases are unknown. On the other hand, the poor fossil record of these upper beds could in part be the result of lower sedimentation rates that would directly affect the taphocenosis phase, with skeletal remains being more intensely weathered, reworked, or scavenged, leading to reduced fossil preservation. Although equally extensive fossil prospecting has been carried out in the upper levels of the Ischigualasto Formation in the Hoyada del Cerro Las Lajas, sampling biases cannot be completely ruled out as an explanation for the lack of fossils. Indeed, a combination of the above factors may explain the taphonomy of the Ischigualasto Formation and the scarcity of fossils from its upper levels.

### Faunal correlations

The fossil collection effort undertaken in the Ischigualasto Formation at the Hoyada del Cerro Las Lajas clearly did not produce a sampling as complete as that available for the IPP. Thirty-five specimens were identified to the genus level, compared to the nearly 1,000 specimens identified over the last 25 years in the IPP^3^. Yet, some significant faunal patterns have emerged at the Hoyada del Cerro Las Lajas that deserve further scrutiny. One of these is the stratigraphic separation between the sampled rhynchosaur genera, with *Hyperodapedon* occurring up to 260 mab and *Teyumbaita* occurring immediately above that. In contrast, the range of the traversodontid cynodont *Exaeretodon* spans nearly the entire fossil-bearing strata (120–400 mab), as is the case in the IPP^3^, whereas the archosauriforms *Aetosauro. scagliai* and *Pro*. *barrionuevoi* have single records together with *Hyperodapedon* and *Teyumbaita*, respectively. This is also respectively the case for the inferred provenances of *Pi. mertii* and *V. rusconii* plus *Tri. romeri* (see above).

A two-fold subdivision of the studied assemblages is, therefore, conceivable (Fig. 5), with an older fauna including *Hyperodapedon*, *Exaeretodon*, *A. scagliai*, and possibly *Pi. mertii*, occurring between 115 and 260 mab, succeeded by a younger fauna with *Teyumbaita*, *Exaeretodon*, *Pro*. *barrionuevoi*, and possibly *V. rusconii* and *Tri. romeri*, between 260 and 350 mab. Based on the dominant rhynchosaurs, these assemblages are herein referred to as *Hyperodapedon* and *Teyumbaita* biozones, respectively*.* The former was recorded above the ‘Toba-2’ tuff dated at 229.25 ± 0.10 Ma, with the 228.97 ± 0.22 Ma tuff positioned within the beds with *Hyperodapedon*. As for the *Teyumbaita* biozone, our age-depth model (see above) constrains it to between ca. 227.94 + 0.83/− 1.67 and 227.24 + 1.27/− 1.97 Ma. The stratigraphically highest fossils (*Exaeretodon* sp. and an indeterminate rhynchosaur), occurring at 400 mab, correspond to an interpolated age of 226.85 + 1.45/− 2.01 Ma based on the age model.

The fossil record of the Ischigualasto Formation in the IPP is clustered in the first 300 m of the section, i.e. the *Scaphonyx*-*Exaeretodon*-*Herrerasaurus* biozone^3^, but extends over the next ca. 300 m of the section, especially with the record of *Exaeretodon* sp. in the eponymous biozone. Yet, the most noticeable biostratigraphic pattern seen in IPP is the high abundance of the rhynchosaur “*Scaphonyx*” *sanjuanensis* in the lower 100 m of the section, with the taxon decreasing in abundance in the next 200 m, until it disappears at 300 m^3^. A key discussion involves the identification of the IPP rhynchosaurs. “*Scaphonyx*” is a *nomen dubium* that may refer to any hyperodapedontine rhynchosaur, and the main taxon identified in IPP is better referred to as *Hyperodapedon sanjuanensis*^29,40^. Indeed, this species represents the totality of the rhynchosaurs recorded in the Hoyada de Ischigualasto^3^.

The biostratigraphic patterns recognized in the Hoyada del Cerro Las Lajas have some resemblances to those of IPP. The cynodont *Exaeretodon* is the taxon with the broadest range in both areas. However, the significantly lower collection effort in the Hoyada del Cerro Las Lajas hampers a confident estimate of the *Exaeretodon* range. As such, the upper ca. 700 m of the Ischigualasto Formation at the Hoyada del Cerro Las Lajas may be devoid of fossils in part due to insufficient sampling, although a less abundant fossil record in the upper portions of the Ischigualasto Formation is also seen in IPP and fits the taphonomic model proposed above.

**Figure 12:**
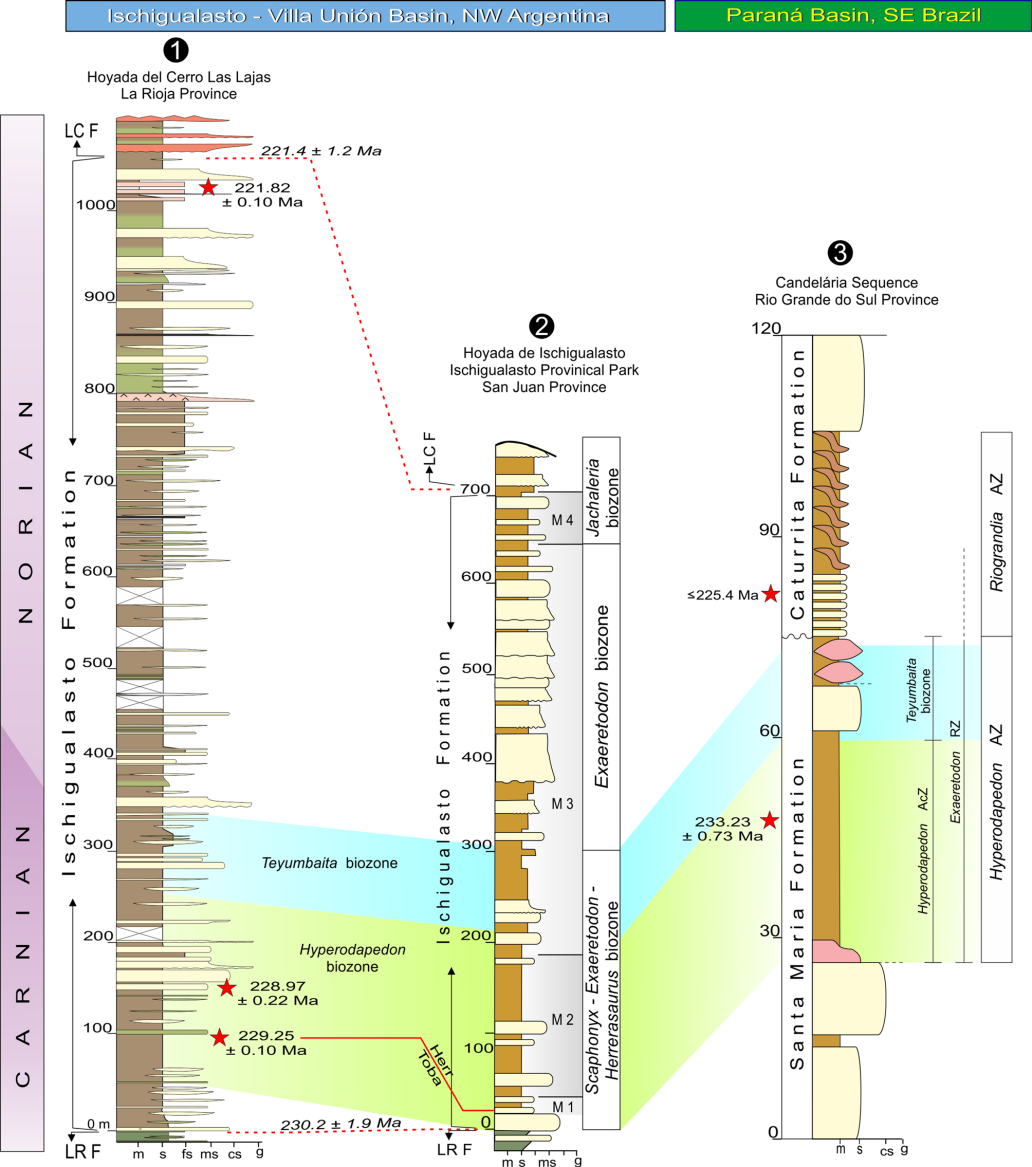
Lithostratigraphy, vertebrate biostratigraphy, and new U–Pb geochronology of the Ischigualasto Formation at (1) Hoyada del Cerro Las Lajas (this study) and (2) Hoyada de Ischigualasto^3^ in NW Argentina, and their correlation to the (3) Late Triassic of Rio Grande do Sul in southern Brazil^146^. Stars mark tuff samples dated by the U–Pb CA-ID-TIMS method. Santa Maria and Caturrita formation biostratigraphy and geochronology^93^. Numbers in italics over dashed lines signify ages interpolated from dated tuffs using a Bayesian age model. Rock legend as in Fig. 3. AZ = assemblage zone; LCF = Los Colorados Formation; LRF = Los Rastros Formation; M1–M4 = Ischigualasto Formation members. Figure generated by some authors (L.E.F., A.A.S.D.R., and J.R.) using Corel Draw X5 software and based on our own geological studies in the each region (western Argentina and southern Brazil).

We speculate that the replacement of *Hyperodapedon* by *Teyumbaita* at about 260 mab may not be simply a preservation artefact, in part based on the high abundance of fossils at this level. Based on our age model, the *Hyperodapedon* and *Teyumbaita* biozones in the Hoyada del Cerro Las Lajas may respectively correlate (Fig. 12) to the lower and upper parts of the *Scaphonyx*-*Exaeretodon*-*Herrerasaurus* biozone at IPP^3^. Another interesting biostratigraphic pattern found in the Hoyada del Cerro Las Lajas is the abundance of *Teyumbaita* around 260–300 mab. Although in much lower numbers because of a poorer sampling, this compares with the likely older *Hyperodapedon* proportional richness at the base of IPP sections. Preliminary reports mentioned the presence of *Te. sulcognathus* (= “*Scaphonyx*”* sulcognathus*) in the “upper” levels of the Ischigualasto Formation in the Hoyada de Ischigualasto^91,92^. Yet, these reports did not provide collection numbers for the putative *Teyumbaita* specimens, which could not be located in the collections or restudied here, neither there is precise information of how “upper” were these specimens collected in the section of IPP. In any case, these putative *Teyumbaita* records in IPP were reported as stratigraphically above those of “*Scaphonyx*” *sanjuanensis*, matching the biostratigraphic pattern described here for the Hoyada del Cerro Las Lajas. Only a broad alpha-taxonomy revision of the *Hyperodapedon*-clade^93^ specimens of IPP would shed light on the biostratigraphic distribution of the rhynchosaurs in that area^38,40^. It would be important to see if *Teyumbaita* occurs in that area and, if present, whether or not it is stratigraphically separated from *Hyperodapedon* as in the Hoyada del Cerro Las Lajas. This will be a test of the rhynchosaur turnover as a useful biostratigraphic marker.

Previously, the rhynchosaur genus *Teyumbaita* was only conclusively recognised in the Late Triassic beds of the Santa Maria Supersequence^94^ in southern Brazil (Fig. 12). Montefeltro et al.^41^ revised the three better-known records of the taxon, which was found above those of *Hyperodapedon* in all sites it occurs, matching the pattern seen in the Hoyada del Cerro Las Lajas. All the records of *Teyumbaita* in Brazil are isolated, with no other associated index-fossils in beds of the same site. Accordingly, such *Teyumbaita*-bearing beds cannot be directly correlated with putatively coeval strata from other Triassic sites in southern Brazil, such as those where *Exaeretodon* abound in the absence of confirmed records of *Hyperodapedon*^95,96^. In fact, one previous record of *Hyperodapedon* (based on specimen MCN 3509PV), along with *Exaeretodon* in the Janner site^96–98^, is yet to be confirmed and, until further scrutiny, MCN 3509PV is safely assigned only to the *Hyperodapedon*-clade^34^. In any case, the Argentinean record of *Teyumbaita* supports the correlation of the strata where *Exaeretodon* is more abundant than *Hyperodapedon* in that country (i.e. upper *Scaphonyx*-*Exaeretodon*-*Herrerasaurus* and *Exaeretodon* biozones)^3^ with the southern Brazilian beds where *Teyumbaita* and/or *Exaeretodon* occurs/abounds in the absence of *Hyperodapedon*^95^, i.e. *Exaeretodon* sub-assemblage zone^99^. The inferred ca. 228–227 Ma age for the *Teyumbaita*-rich beds in Argentina also matches the recently reported radioisotopic ages for the *Hyperodapedon* and *Riograndia* Assemblage Zones in Brazil^93^. These have been dated at ca. 233 and 226 Ma, respectively, and are consistently positioned below and above (Fig. 12) the *Teyumbaita* beds in the proposed stratigraphic schemes of the Santa Maria Supersequence^94^.

The Late Triassic tetrapod faunal compilation of South America^93^ revealed a gap in the tetrapod fossil record of western Pangaea near the Carnian-Norian boundary, bounded from below by the lower Ischigualasto Formation fauna, and from above by the faunas of the upper Ischigualasto Formation and *Riograndia* Assemblage Zone. The revised age model for the Ischigualasto Formation presented here essentially fills that purported gap, showing the continuity of a faunal structure recognized in strata such as those of the *Hyperodapedon* Assemblage-Zone in Brazil (dated at ca. 233 Ma)^93^ across that stage boundary, at least in palaeolatitudes close to 40°–50° South. For example, rhynchosaurs still abound and proterochampsids still occur in such strata, differing from younger beds that lack such taxa^93^.

Therefore, it seems that the major Late Triassic turnover seen in the terrestrial tetrapod biotas of western Gondwana post-dates the assemblages currently known for the Hoyada del Cerro Las Lajas (and most probably the Carnian-Norian boundary), which is followed by Norian non-fossiliferous deposits that evidence an increase in humidity (see above). This has a significant impact on the first occurrence of tetrapod groups such as saurischian dinosaurs and crocodylomorphs in the fossil record.

In Brazil, the ca. 226 Ma (early Norian) dated beds in which the *Riograndia* Assemblage Zone was recorded preserve sedimentary environments that drastically depart from those yielding the *Hyperodapedon* Assemblage Zone (including the *Teyumbaita*-bearings beds), the lower part of which was dated as ca. 233 Ma. This transition represents the replacement of an ephemeral anastomosed fluvial-lacustrine system (Alemoa Member of the Santa Maria Formation) by a perennial braided fluvial system (Caturrita Formation)^100^, which indicates a pluviosity increase, as also suggested by Th/U geochemical data^101^. As such, it seems that the Norian onset in southwestern Pangaea was marked by a humidity increase, as seen in the Caturrita Formation in Brazil and the upper levels of the Ischigualasto Formation in Argentina, after a more arid period that itself post-dated the Carnian Pluvial Event^102,103^. This coincides with a major biotic turnover^93^, when faunas with rhynchosaurs, proterochampsids, and herrerasaurid dinosaurs were replaced by faunas with the oldest plateosaurian sauropodomorphs^104^, the oldest (and only reported) South American phytosaurs^105^, together with some of the few post-Carnian dicynodont records^3,106^.

### *Pisanosaurus mertii* and dinosaur origins

*Pisanosaurus mertii* Casamiquela^4^ has been for a long time regarded as the oldest known ornithischian, but its dinosaur affinity was recently challenged by Agnolín and Rozadilla^107^, as well as in briefer accounts by Baron et al.^108^ and Baron^109^. A comprehensive historical account of the taxon relationships has been provided^8^ and there is no need to be duplicated here. Suffice to say, apart from broadly expressed scepticism^110–114^, the ornithischian affinity of *Pi. mertii* was only questioned on numerical phylogenetic grounds by the three papers mentioned above, and the matrices in Baron et al.^108^ and Baron^109^ are not independent from one another. In those hypotheses, the proposed alternative was to nest *Pi. mertii* among silesaurids, a dinosauromorph group usually positioned immediately outside the Dinosauria (but see Langer and Ferigolo^115^). Given the potential evolutionary importance of this Hoyada del Cerro Las Lajas taxon, here we review the anatomical evidence brought forward by Agnolín and Rozadilla^107^ in support of the silesaurid affinity of *Pi. mertii*, as well as features that may instead suggest its ornithischian affinity.

Agnolín and Rozadilla^107^ provided a compelling review of the anatomy of *Pi. mertii* and supported previous arguments claiming that the elements of its holotype belong to a single individual. Yet, a bone fragment that seems to represent a partial right femoral shaft was found among the *Pi. mertii* material^116^. It has an asymmetric fourth trochanter, as seen in non-neotheropod saurischian dinosaurs, in contrast to the pendant trochanter of ornithischians. This possible femur fragment has a preserved length of ca. 1.5 cm and a transverse width slightly below 1 cm, thus belonging to an individual considerably smaller than the holotype of *Pi. mertii*. As a result, we agree with Sereno^116^ that bones of a smaller reptile—possibly an early saurischian dinosaur—may be stored together with and probably were associated to the holotype of *Pi. mertii*. Nevertheless, we still adhere to previous claims that the bones historically associated to *Pi. mertii*—which do not include this probable femur—belong to a single individual based on the matching size of the bones and field data describing the degree of articulation of the specimen when it was collected^107^. Here, we do not aim to review the anatomy of *Pi. mertii*, but regard this as a much-needed future enterprise, especially if assisted by non-destructive, tomographic techniques. Instead, we focus on revising anatomical traits that might help resolving the contentious placement of the taxon as either an ornithischian or a silesaurid.

**Figure 13:**
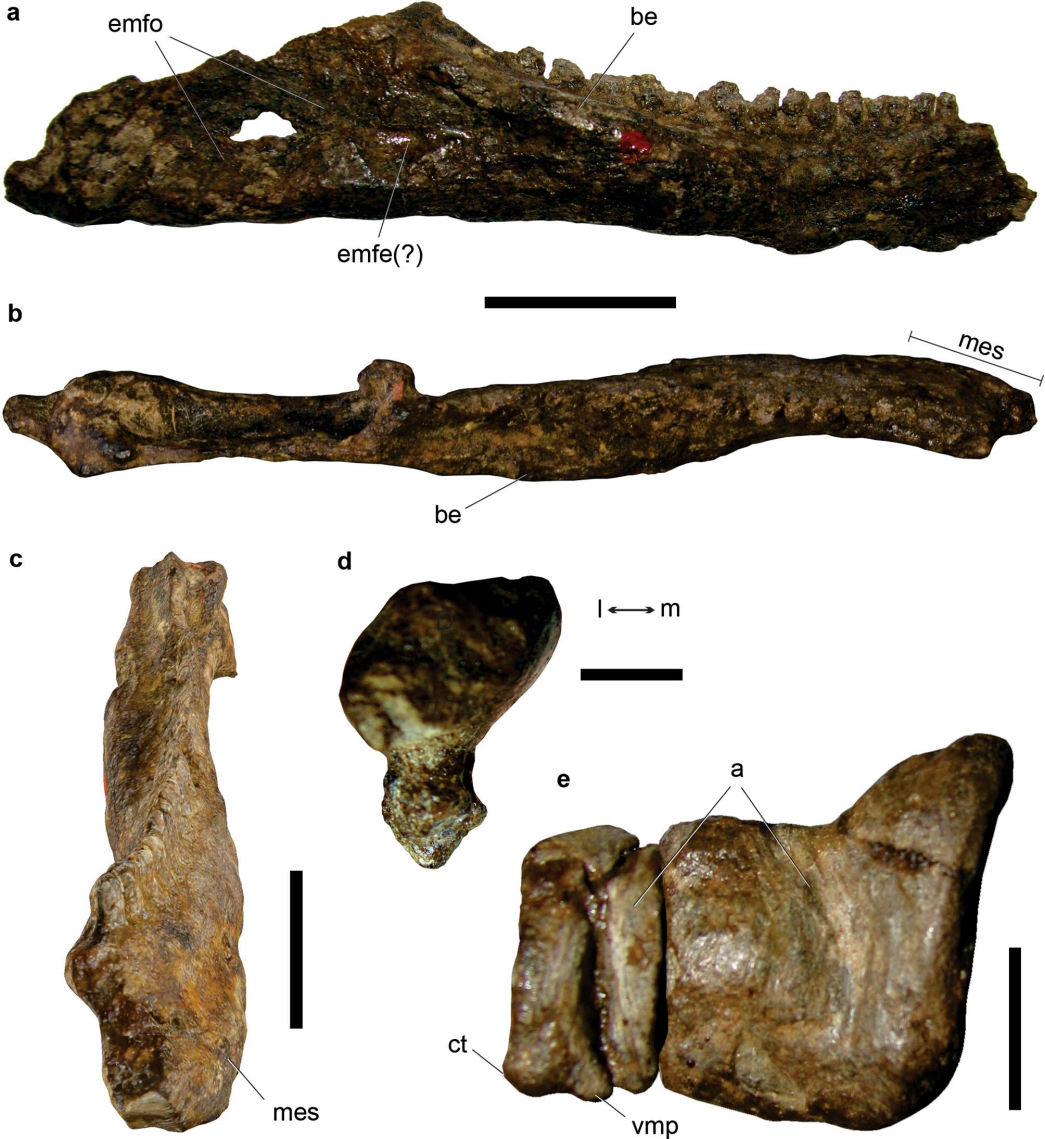
*Pisanosaurus mertii* (holotype, PVL 2577), key-anatomical features of jaw and tarsus. Right hemimandible in (**a**) lateral, (**b**) dorsal, and (**c**) anterodorsal views. (**d**) Posterior view of right maxillary teeth. (**e**) Right astragalus and calcaneum in distal view, note that the astragalus has been split in two parts during preparation. Abbreviations: a, astragalus; be, buccal emargination; ct, calcaneal tuber; emfe, external mandibular fenestra; emfo, external mandibular fossa; mes, medially expanded symphysis; vmp, ventromedial projection. Scale bars: (**a**) 2 cm, (**b**) 3 mm, and (**c**–**d**) 1 cm.

We agree with Agnolín and Rozadilla^107^ that the specimen preservation does not allow the positive identification of an external mandibular fenestra in *Pi. mertii*, but neither allows to confirm its absence. Yet, *Pi. mertii* has a depressed area on the lateral surface of the post-dentary portion of the hemimandible (Fig. 13a) that is recognized only in heterodontosaurids, i.e. “external mandibular fossa” of Sereno^116^, among early dinosauromorphs^117–119^. Its more deeply excavated anteroventral corner is in a position similar to that occupied by the external mandibular fenestra of ornithischians^116^. Indeed, a reduced fenestra could be an evidence of the ornithischian affinity of *Pi. mertii*, whereas its putative absence would be autapomorphic for *Pi. mertii*.

For about two centimetres posterior to the broken tip of the bone, the medial surface of the dentary of *Pi. mertii* is medially expanded at its ventral margin (Fig. 13b, c). A similar condition is seen in some ornithischians^116,119^, but not in silesaurids^115,120^. Also, the dentary of *Pi. mertii* possesses a gradual torsion towards its anterior end, where the cross-section of the bone gets dorsolateral to ventromedially oriented (Fig. 13c). This condition is present in at least some ornithischians (e.g. *Eocursor parvus*, SAM-PK-K8025), but absent in silesaurids and other early dinosauromorphs. The presence of a tall coronoid process on a relatively short dentary, producing a strongly concave dorsal margin of the bone in lateral-medial views, also resembles more the condition in heterodontosaurids^17^ than in silesaurids^121^, saurischians^93,117,122^, and even other ornithischians^119,123^; but a better quantification of these differences must be provided before they can be used to infer the affinities of *Pi. mertii*. Also, the apparent lack of replacement foramina in the *Pi. mertii* lower jaw may support its heterodontosaurid affinities within ornithischians^116^, as those are present in other early members of that group^123,124^, as well as in silesaurids^115,125^.

The dentary of *Pi. mertii* has a strong, bulbous ridge extending below the tooth row, especially at its posterior half, forming a “buccal emargination”^116^, between the ridge and the teeth (Fig. 13a,b). A similar structure also appears to extend above the maxillary tooth row, but this is harder to confirm given the incomplete preservation of that bone. Such a strong ridge and emargination is absent in silesaurids^115^ and dinosaurs in general, including early sauropodomorphs^93,122^. On the contrary, this is seen in several^116,119,126–128^, although not all, early ornithischians^123,124,129^. Accordingly, the presence of a buccal emargination in *Pi. mertii* better supports an ornithischian, rather than silesaurid affinity.

Inferring the mode of tooth attachment in *Pi. mertii* is exceedingly hard because of poor preservation (and conjectural) without the support of CT-Scan or histology techniques. Thus (*contra* Agnolín and Rozadilla^107^), until more detailed data is available, we consider that tooth attachment cannot be used to infer either affinity (i.e. silesaurid or ornithischian) for *Pi. mertii*. Nevertheless, repreparation of the base of one of the maxillary teeth revealed the absence of fusion to the surrounding bone. Most preserved tooth crowns of *Pi. mertii* are broader labiolingually than mesiodistally (Fig. 13b), or at least equally broad in those two axes^107^. This is a very unusual condition among early dinosauromorphs and is approached only by the highly modified molariform teeth of some heterodontosaurids^116^. We agree with Agnolín and Rozadilla^107^ that tooth crowns of *Pi. mertii* lack signs of carinae or denticles, but we disagree with those authors (p. 22)^107^ in that the lack of denticles support a silesaurid affinity for *Pi. mertii*, as these elements are clearly seen in most representatives of the group^115,120,130^. Unlike Agnolín and Rozadilla^107^, we found it difficult to define enamel thickness or the presence of longitudinal ridges in the *Pi. mertii* teeth. Yet, we concur with those authors that a blunt primary ridge, like that of ornithischians^116,123,124,127^ is seen in some teeth. This produces a morphology that most closely resembles that of the constricted, cup-shaped bases of *Heterodontosaurus tucki* and *Lycorhinus angustidens* molariform crowns^116,131^. Similarly, the maxillary tooth crowns of *Pi. mertii* are medially inclined (Fig. 13d), resembling the condition in *He. tucki* (SAM-PK-K1332), but contrasting with the vertical teeth of silesaurids. Finally, tooth crowns of *Pi. mertii* are only similarly short as those of some silesaurids if these are compared to the “blade-like” crowns of plesiomorphic putative members of the group such as *Lewisuchus admixtus*^132,133^. Accordingly, *contra* Agnolín and Rozadilla^107^, short tooth crowns cannot be used *prima facie* (i.e. in the absence of a phylogenetic framework) to infer a silesaurid affinity for *P. mertii*.

We agree with Bonaparte^134^ (*contra* Casamiquela^4^) that a pair of isolated vertebrae of *Pi. mertii* represent cervical elements. The centra are parallelogram-shaped in lateral view, a condition unrecognized in early dinosauromorph tail vertebrae. As such, they more closely resemble the short cervical elements of early ornithischians^116,135^ than the more elongated vertebrae of silesaurids^136^. The trunk vertebrae of *Pi. mertii* are generally compressed lateromedially, with tall neural arches (about as deep as the centra) and well-developed prezygadiapophyseal, postzygadiapophyseal, prezygaparapophyseal, and anterior and posterior centrodiapophyseal laminae (see fig. 6 in Agnolín and Rozadilla^107^). This condition is very similar to that of *Silesaurus opolensis*^136^, markedly departing from the morphology of early ornithischian trunk vertebrae, which are not as lateromedially compressed and have dorsoventrally short neural arches with poorly defined or no lamination^116,123, 135,137^. As for the impressions of the sacral vertebrae, we agree with most authors that there is evidence of at least four elements and that the sacral ribs are shared between two vertebrae. Silesaurids have either two or three sacral vertebrae^120,121^ and sacral ribs shared between two vertebrae are seen in both silesaurids^120,130^ and early ornithischians^132,138^ (*Scelidosaurus harrisonii*, NHMUK PV R1111). Hence (*contra* Agnolín and Rozadilla^107^), the latter trait cannot be employed *prima facie* to infer a silesaurid affinity for *Pi. mertii*.

Pelvic features are very hard to identify, including if the acetabulum was open or closed, even if partially. Yet, we concur with Sereno^124^ that none of the modifications seen in the opisthopubic pelvis of ornithischians can be recognized in *Pi. mertii*. On the contrary, the puboischial articulation is dorsoventrally extended and the ischial symphysis is not restricted to the distal end of the bone, suggesting a plesiomorphic propubic pattern. The popliteal fossa of the femur of *Pi. mertii* seems to be overprepared along its distal two centimeters, but we agree with Agnolín and Rozadilla^107^ that ridges surrounding the fossa can be traced along the five distal centimeters of the bone (see fig. 11 in Agnolín and Rozadilla^107^). The associated tibia is about 16 cm long, so we could infer a minimal femoral length of 15 cm, suggesting that such ridges extended over the distal third of the bone. This condition matches that of silesaurids, in which the popliteal fossa extends for more than one fourth of the femoral length^132^, but conditions similar to that of *Pi. mertii*, with rather subtle proximally extending ridges, are seen in various early dinosauromorphs, including ornithischians^127^ (*Scutelosaurus lawleri*, MNA V175; *Laquintasaura venezuelae*). Also (*contra* Agnolín and Rozadilla^107^), the cranial intermuscular line of early dinosaurs does not usually reach the distal third of the bone^139,140^, so that its absence in the preserved portion of femur of *Pi. mertii* is not evidence for its non-dinosaurian affinity.

As stated by Agnolín and Rozadilla^107^, the tibia of *Pi. mertii* is indeed devoid of a cnemial crest that expands proximally relative to the femoral facet. This configuration is very similar to that of *Sacisaurus agudoensis*^115^, departing from the typical dinosaur condition, including that of most ornithischians^116,127,138^ (*Sc. lawleri*, MNA V175). Therefore, the reduced cnemial crest of *Pi. mertii* seems to better fit a silesaurid affinity. Yet, this character has a more complex distribution, with proximally unexpanded crests seen in undescribed specimens of *La. venezuelae* and the holotype of *Lesothosaurus diagnosticus* (NHMUK PV RU B17), and a more projected crest is seen in *Asilisaurus kongwe*^120^. A fibular crest as that of most theropods and silesaurids is indeed seen in the tibia of *Pi. mertii* and lacking in most early ornithischians^127,135,138^ (*Sc. lawleri*, MNA V175), but we see no reason (*contra* Agnolín and Rozadilla^107^) to disregard its homology to the crest present in heterodontosaurids^141^. In addition, the posterior hemicondyles of the proximal end of the tibia are separated from one another by a deep and very transversely broad notch (see Fig. 6l in Irmis et al.^111^) that closely resembles the condition in several early ornithischians (e.g. *He. tucki*, SAM-PK-K1332; *Eo. parvus*, SAM-PK-K8025; *Sc. lawleri*, UCMP 130580). By contrast, the posterior hemicondyles of the tibia are separated by a distinct change in slope or a narrow groove in other early dinosauromorphs.

The articulation with the astragalus hampers a proper assessment of the distal outline of the tibia, but it is possible to infer that it is at least as broad anteroposteriorly as lateromedially, as occurs in most silesaurids^120,130^ and early dinosaurs, such as herrerasaurids and early sauropodomorphs^122,142^. Instead, neotheropods, ornithischians^142^, and *Sa. agudoensis*^115^, have a much more lateromedially expanded distal end of the bone. We agree with Agnolín and Rozadilla^107^ that the posterolateral margin of the tibia of *Pi. mertii* is not concave as in early ornithischians (*L. diagnosticus*, NHMUK PV RU B17; *Sc. lawleri*, MNA V1752), but we disagree that this feature supports its silesaurid affinity, as a similar plesiomorphic condition is also seen in most early dinosauromorphs, including dinosaurs^142^. As for the descending process of the tibia (= outer malleolus or posterolateral process) of *Pi. mertii*, it expands only slightly lateral to the anterolateral corner of the distal end of the bone (see fig. 12c in Agnolín and Rozadilla^107^), resembling the condition of *Asili. kongwe*^120^, early sauropodomorphs, and herrerasaurids^122,142^, but markedly differing from neotheropods, ornithischians^142^, and *Sa. agudoensis*^115^, which bear an extensive descending process. Also, the descending process is well developed in some specimens of *Si. opolensis* (ZPAL Ab III 413, 415), although not as much as in ornithischians, whereas other specimens of that silesaurid (ZPAL Ab III 403/1, 460/3) bear a short process as in *Pi. mertii*. Finally, as mentioned by Agnolín and Rozadilla^107^ the distal part of the fibula in *Pi. mertii* is not as slender as that of ornithischians^116,123,135^, retaining instead the transversely broader condition that is plesiomorphic among dinosauromorphs.

The anteroposterior breadth of the lateral margin of the astragalus of *Pi. mertii* is about three-fourths of its lateromedial width (Fig. 13e). Thus, that bone is proportionally less lateromedially expanded than in silesaurids^120,130^ and early saurischians^122,139,143,144^, but has similar proportions to that of ornithischians (*Sc. lawleri*, MNA V175)^141^. Similarly, the proximodistal height of the astragalar ascending process with respect to the height of the astragalar body (see fig. 12d,e in Agnolín and Rozadilla^8^) resembles more the condition in ornithischians (*Sc. lawleri*, MNA V175) than the shallower process of silesaurids^120,130^. However, the lateromedially narrow and medially sloping astragalar ascending process of *Pi. mertii*, as seen in anterior view (see fig.12c in Agnolín and Rozadilla^107^), resembles the condition in silesaurids more than that of early ornithischians.

The calcaneum of *Pi. mertii* (Fig. 13e) retains a well recognizable calcaneal tuber and an expanded posteromedial corner of the bone, forming the “ventromedial projection”^143^. This shape is a modification of the general plesiomorphic subtriangular calcaneum seen in most early dinosauriforms^120,139,143^, including silesaurids^120,130^. Accordingly, *contra* Agnolín and Rozadilla (p. 22)^107^, the calcaneal tuber does not support the non-dinosaurian affinity of *Pi. mertii*. Also, the calcaneum of *Pi. mertii* is lateromedially compressed, resembling the condition in the paratype of *Sc. lawleri* (MNA V1752) and heterodontosaurids^116,129,141^. The anterior and lateral margins of the calcaneum-fibula articulation reveal a convex calcaneal facet, with a slightly straighter posterior part, whereas the medial view reveals a concave calcaneum margin (see fig. 13a,b in Agnolín and Rozadilla^107^). As discussed by Agnolín and Rozadilla (p. 11, 19)^107^, but with the sides reversed, such a complex articulation is more typical of non-dinosaurian archosaurs, although lacking in lagerpetids and *Si. opolensis*^143,145^. On the other hand, the fibula-calcaneum articulation of *Pi. mertii* clearly differs from that of ornithischians, in which the main basin that occupies the posterior two-thirds of the proximal articulation of the calcaneum receives the outer malleolus of the tibia, whereas the fibula articulated only to the top of the raised anterior third of the bone^129,141^ (*Sc. lawleri*, MNA V1752).

Agnolín and Rozadilla^107^ mentioned that metatarsal IV of *Pi. mertii* resembles those of silesaurids and saurischians because it is compressed at the proximal end. Indeed, the available data for early ornithischians (*Le. diagnosticus*, NHMUK PV RU B17; *He. tucki*, SAM-PK-K1332) reveal a more robust proximal articulation of metatarsal IV. On the contrary, *contra* Agnolín and Rozadilla (p. 22)^107^, the ungual phalanx of the fourth pedal digit of *Pi. mertii*, and the only available for the taxon (see Fig. 15 in Agnolín and Rozadilla^107^), lacks the marked dorsoventral flattening present in silesaurids, contradicting a possible silesaurid affinity.

The features discussed above (Table 2) show that most character-states previously used to support a silesaurid affinity for *Pi. mertii* are likely plesiomorphic for Dinosauriformes, being also present in other dinosauromorphs and early dinosaurs. Some other characters are variable among silesaurids and ornithischians. Conversely, we found over ten character-states that are shared only by *Pi. mertii* and heterodontosaurids and/or other early ornithischians among early dinosauriforms. As a result, we consider that the ornithischian affinity of *Pi. mertii* rests on much stronger grounds than the silesaurid hypothesis. A quantitative analysis of the phylogenetic relationships of *Pi. mertii* goes beyond the scope of this paper and it will be conducted in the near future, integrating the new information provided here.

**Table 2:** Anatomical evidence supporting the alternative (Silesauridae vs Ornithischia) affinities of *Pisanosaurus mertii*.

Silesauridae	Ornithischia
Well-developed laminae in trunk vertebrae^†^	Reduced external mandibular fenestra*
Trunk vertebrae with tall neural arches^†^	External mandibular fossa (h)
Propubic pelvis^†^	Medially expanded mandibular symphysis*
Cnemial crest unexpanded proximally^∆^	Torsion of the anterior portion of dentary^∆^
Not lateromedially expanded distal tibia^†∆^	No dentary replacement foramina (h)*
Unexpanded outer malleolus^†∆^	Strongly concave dorsal margin of the dentary (h)
Unconstricted distal end of the fibula^†^	Buccal emargination^∆^
Lateromedially wide aap^†^	Labiolingualy broad tooth crowns (h)
Large fibular facet on the calcaneum^†^	Medially inclined maxillary tooth crowns (h)
Compressed proximal portion of metatarsal IV^†^	Tooth crowns with primary ridge
	Anteroposteriorly short cervical vertebrae
	More than three sacral vertebrae
	Broad and deep posterior notch on proximal tibia
	Lateromedially narrow astragalus
	Proximodistally deep aap^†^
	Lateromedially compressed calcaneum

*aap* = astragalar ascending process; *h* = seen only in heterodontosaurids among ornithischians; * = poorly preserved in *Pi. mertii*; *∆* = variable among silesaurids and/or early ornithischians; *†* = likely plesiomorphic among dinosauriforms.

As discussed by previous authors^107,109,111,116,124^, considered as an ornithischian, *Pi. mertii* fills the long ghost lineage between other members of the group (Early Jurassic)^109^ and the oldest known saurischians (ca. 233 Ma)^93^. Here, we constrain the age of *Pi. mertii* as ca. 229 Ma, showing that this species is latest Carnian. As a result, the long ghost lineage is transferred into the ornithischian clade, with the group absent in the fossil record for more than 30 My. *Pisanosaurus mertii* provides key clues about the early evolutionary history of Ornithischia and it is thus one of the most important components of the Hoyada del Cerro Las Lajas fauna. However, at the same time, it shows how deficient is our current knowledge of the first million years of evolution of this main dinosaur lineage.

## Conclusions

- The most complete succession of the Late Triassic Ischigualasto Formation is exposed in the Hoyada del Cerro Las Lajas, consisting of more than 1,000 m of fluvial-channel and flood overbank deposits with high volcanic input, which have produced historical tetrapod fossils.
- High precision ^206^Pb/^238^U geochronology based on three interbedded tuffs located at 107, 160, and 1,035 m above base produced weighted mean dates of 229.25 ± 0.10/0.16/0.30, 228.97 ± 0.22/0.23/0.33 Ma, and 221.82 ± 0.10/0.12/0.27 Ma, respectively. Bayesian age interpolations based on the new geochronology constrains the deposition of the Ischigualalsto Formation from 230.2 ± 1.9 Ma and 221.4 ± 1.2 Ma.
- Tetrapod fossils are concentrated in the lower third of the succession at the Hoyada del Cerro Las Lajas, where two faunal associations are identified: (a) a *Hyperodapedon* biozone, ranging from 115 to 260 mab, records *Hyperodapedon* (including *H*. *sanjuanensis*), *Exaeretodon*, and *Aetosauroides* and, (b) a *Teyumbaita* biozone, ranging from 260 to 350 mab, records *Teyumbaita*, *Proterochampsa*, and *Exaeretodon*. The entire fossiliferous interval, between 115 and 400 mab, corresponds to ca. 229.20 + 0.11/− 0.15–226.85 + 1.45/− 2.01 Ma based on the maximum age range (with errors) from our Bayesian age-stratigraphic model.
- Based on the petrographic comparisons of the sedimentary rock matrix of their holotypes, *Pi. mertii* and *V*. *rusconii* have been inferred to stratigraphically correspond to the *Hyperodapedon* and *Teyumbaita* biozones, respectively.
- The age-calibrated biostratigraphy of the Ischigualasto Formation correlates the *Hyperodapedon* and *Teyumbaita* biozones at the Hoyada del Cerro Las Lajas, respectively, to the lower and upper parts of the *Scaphonyx*-*Exaeretodon*-*Herrerasaurus* biozone in the Hoyada de Ischigualasto and to the upper *Hyperodapedon* Assemblage Zone of the Santa Maria Supersequence in southern Brazil. Our chronostratigraphic model constrains the *Hyperodapedon* biozone between 229.20 + 0.11/− 0.15 to 227.94 + 0.83/− 1.67 Ma, and the immediately overlying *Teyumbaita* biozone up to 227.24 + 1.27/− 1.97 Ma.
- The *Teyumbaita*-rich faunas of both Brazil and Argentina suggest that the typically Carnian rhynchosaur-dominated dryland faunal association persisted into the Norian, before it was eventually replaced by tetrapod assemblages that witnessed the humidity increase of southwestern Pangaean climate.
- The preferred ornithischian affinity of *Pi. mertii* based on a thorough review of its anatomical traits, together with its chronostratigraphic age of ca. 229 Ma, places the oldest documented ornithischian dinosaur in the latest Carnian. This fills the long-speculated ghost lineage between younger members of that clade and the oldest known saurischian dinosaurs at ca. 233 Ma.

## Methods

### Institutional abbreviations

**CPEZ,** Coleção de Paleontologia do Museu Paleontológico e Arqueológico Walter Ilha, São Pedro do Sul, Brazil. **CRILAR-Pv,** Paleontología de Vertebrados, Centro Regional de Investigaciones Científicas y Transferencia Tecnológica, Anillaco, Argentina; **GPIT/RE,** Institut für Geowissenschaften, Universität Tübingen, Tübingen, Germany. **ISIR**, Indian Statistical Institute, Reptiles, Kolkata, India. **MCN,** Museu de Ciências Naturais, Fundação Zoobotânica do Rio Grande do Sul, Porto Alegre, Brazil. **MACN-Pv,** Museo Argentino de Ciencias Naturales “Bernardino Rivadavia”, Colección Paleovertebrados, Buenos Aires, Argentina. **MSM,** Arizona Museum of Natural History, Mesa, Arizona, USA (formerly Mesa Southwest Museum). **NMMNH-P,** New Mexico Museum of Natural History, New Mexico, USA. **NHMUK PV,** Natural History Museum, London, UK. **PEFO,** Petrified Forest National Park, Arizona, USA. **PULR,** Paleontología, Museo de Ciencias Naturales, Universidad Nacional de La Rioja, La Rioja, Argentina. **PVL,** Paleontología de Vertebrados, Instituto Miguel Lillo, Tucumán, Argentina. **PVSJ,** División de Paleontología de Vertebrados del Museo de Ciencias Naturales y Universidad Nacional de San Juan, San Juan, Argentina. **SAM-PK,** Iziko South African Museum, Cape Town, South Africa. **SMNS,** Staatliches Museum für Naturkunde, Stuttgart, Germany. **SNSB-BSPG AS,** Staatliche Naturwissenschaftliche Sammlungen Bayerns, Bayerische Staatssammlung für Paläontologie und Geologie, Munich, Germany. **TMM,** Texas Memorial Museum, Austin, Texas, USA. **TTUP,** Texas Tech University Museum, Lubbock, Texas, USA. **UCMP,** University of California Museum of Paleontology, Berkeley, USA. **UFRGS-PV,** Laboratório de Paleovertebrados, Universidade Federal do Rio Grande do Sul, Porto Alegre, RS, Brazil. **USNM,** National Museum of Natural History (formerly United States National Museum), Smithsonian Institution, Washington D.C., USA. **ZPAL,** Institute of Paleobiology, Polish Academy of Sciences, Warsaw, Poland.

### Sample collection

Vertebrate fossil specimens and rock samples for petrography and U–Pb geochronology were collected on successive field trips from the Ischigualasto Formation at the Hoyada del Cerro Las Lajas.

### Repository

All the vertebrate remains considered in the manuscript, petrographic samples and thin-sections are repositoried at the palaeovertebrate collection (Pv) of the Centro Regional de Investigaciones Científicas y Transferencia Tecnológica de La Rioja (CRILAR), La Rioja Province, Argentina. Geochronologic samples and mineral separates are archived at the MIT Isotope Lab in Cambridge, Massachusetts, USA.

### Geologic mapping

Recorded geologic field information, including lithologic identifications, lithostratigraphic contacts, structural characteristics, and associated GPS coordinates were overlaid onto Google Earth imagery and compiled using CorelDRAW X5 graphics software.

### Stratigraphy

Stratigraphy was carried out in the field during the 2013, 2016, 2017 and 2019. A single, near-straight, W-E transect across the Hoyada del Cerro Las Lajas was made to incorporate lithologic, depositional facies, and structural characteristics, as well as fossil locations (GPS). The data were compiled using CorelDRAW X5 graphics software to construct a single stratigraphic column across nearly 1,000 m of the Ischigualasto Formation exposed at Las Lajas. Details of geological mapping, stratigraphic, and petrographic analyses are given in the Supplementary Information.

### Petrography

Bone and sedimentary rock thin-sections were prepared at the CRILAR Petrographic Laboratory using the protocol described by Fiorelli et al.^147^; specimens were washed with distilled water and cut using a Buehler PetroThin™ device, dried at 40 °C in an oven for 24 h, and subsequently attached with epoxy resin to glass slides of 28 × 48 × 1.8 mm dimensions. Thin-section analyses were made with a Leica DM2500P petrographic microscope. Images were captured with a Leica DFC295 digital camera attached to the microscope and connected to a computer for data processing, editing and measurements. The images and figures were designed and edited with CorelDRAW X5. Petrographic results are described in detail in the Supplementary Information.

### U–Pb geochronology

Several samples of tuff from the Ischigualasto Formation at Hoyada del Cerro Las Lajas were collected for U–Pb zircon geochronology by the CA-ID-TIMS method. Table S2 shows the extended U-Pb zircon geochronology results. Sample processing, isotopic analyses and data reduction was carried out at the Massachusetts Institute of Technology Isotope Laboratory. Details of analytical methods and procedures are fully explained in the Supplementary Information.

### Bayesian age-stratigraphic model

A Bayesian age-depth model has been employed to extrapolate statistically robust ages for the stratigraphic levels of interest (e.g., fossiliferous intervals) constrained by dated tuff beds. For this model, the Bchron software package^148,149^ written for R^150^ was used. Detailed information on age modelling and interpretation can be found in section iii of the Supplementary Information.

## Supplementary information

Supplementary file1

Supplementary file2

## Data Availability

All of the data analysed as part of this study are available in the Supplementary data files.

## References

[1] Bonaparte JF (1997). El Triásico de San Juan—La Rioja Argentina y sus Dinosaurios.

[2] Martínez RN, Sereno PC, Alcober OA, Colombi CE, Renne PR, Montañez IP, Currie BS (2011). A basal dinosaur from the dawn of the dinosaur era in southwestern Pangaea. Science.

[3] Martínez RN, Apaldetti C, Alcober OA, Colombi CE, Sereno PC, Fernández E, Malnis PS, Correa GA, Abelin D (2013). Vertebrate succession in the ischigualasto formation. J. Vertebr. Paleontol..

[4] Casamiquela RM (1967). Un nuevo dinosaurio ornitisquio Triásico (*Pisanosaurus mertii*; Ornithopoda) de la Formación Ischigualasto, Argentina. Ameghiniana.

[5] Bonaparte JF (1970). Annotated list of the South American triassic tetrapods. Counc. Sci. Ind. Res..

[6] Bonaparte JF (1973). Edades/réptil para el Triásico de Argentina y Brasil. Actas Congr. Geol. Argent..

[7] von Baczko MB, Desojo JB, Pol D (2014). Anatomy and phylogenetic position of *Venaticosuchus rusconii* Bonaparte, 1970 (Archosauria, Pseudosuchia), from the Ischigualasto formation (Late Triassic), La Rioja Argentina.. J. Vertebr. Paleontol..

[8] Rogers RR, Swisher CC, Sereno PC, Monetta AM, Forster CA, Martínez RN (1993). The Ischigualasto tetrapod assemblage (Late Triassic, Argentina) and 40Ar/39Ar dating of dinosaur origins. Science.

[9] Cohen KM, Finney SC, Gibbard PL, Fan JX (2013). The ICS international chronostratigraphic chart. Episodes.

[10] Ogg JG, Ogg G, Gradstein FM (2016). A Concise Geologic Time Scale.

[11] Owen R (1861). Palaeontology, or a Systematic Summary of Extinct Animals and Their Geological Relations.

[12] Kemp TS (1982). Mammal-Like Reptiles and the Origin of Mammals.

[13] von Huene F (1936). The constitution of the Thecodontia. Am. J. Sci..

[14] Kammerer CF, Flynn JJ, Ranivoharimanana L, Wyss AR (2008). New material of *Menadon besairiei* (Cynodontia, Traversodontidae) from the Triassic of Madagascar. J. Vertebr. Paleontol..

[15] Cabrera A (1943). El primer hallazgo de terápsidos en la Argentina. Notas del Mus. de La Plata.

[16] Bonaparte JF (1962). Descripción del cráneo y mandíbula de *Exaeretodon frenguellii* Cabrera, y su comparación con Diademodontidae, Tritylodontidae y los cinodontes sudamericanos. Publ. Mus. Munic. Cienc. Nat. Tradic. Mar del Plata.

[17] Abdala F, Barberena MC, Dornelles J (2002). A new species of the traversodontid cynodont *Exaeretodon* from the Santa Maria formation (Middle/Late Triassic) of southern Brazil. J. Vertebr. Paleontol..

[18] Crompton AW (1972). Postcanine occlusion in cynodonts and tritylodontids. Bull. Br. Mus. (Nat. Hist.) Geol..

[19] Liparini A, Oliveira TV, Pretto FA, Soares MB, Schultz CL (2013). The lower jaw and dentition of the traversodontid *Exaeretodon riograndensis* Abdala, Barberena & Dornelles, from the Brazilian Triassic (Santa Maria 2 Sequence, *Hyperodapedon* Assemblage Zone). Alcheringa.

[20] Melo TP, Ribeiro AM, Martinelli AG, Soares MB (2019). Earliest evidence of molariform hypsodonty in a Triassic stem-mammal. Nat. Commun..

[21] Liu J (2007). The taxonomy of the traversodontid cynodonts *Exaeretodon* and *Ischignathus*. Rev. Br. Paleontol..

[22] Liu J, Abdala F, Kammerer CF, Angielczyk KD, Fröbisch J (2014). Phylogeny and taxonomy of the Traversodontidae. Early Evolutionary History of the Synapsida.

[23] Chatterjee S (1982). A new cynodont reptile from the Triassic of India. J. Paleontol..

[24] Gaetano, L. C., Martínez, R. N. & Abdala, N. F. New insights on the cranial anatomy of *Exaeretodon*: intraspecific variation and taxonomic implications. *Reunión de Comunicaciones de la Asociación Paleontológica Argentina*, Puerto Madryn, Libro de Resúmenes, p. 62 (2018).

[25] Pavanatto AEB, Pretto FA, Kerber L, Müller RT, Da-Rosa AAS, Dias-da-Silva S (2018). A new Upper Triassic cynodont-bearing fossiliferous site from southern Brazil, with taphonomic remarks and description of a new traversodontid taxon. J. S. Am. Earth Sci..

[26] Osborn HF (1903). The reptilian subclasses Diapsida and Synapsida and the early history of the Diaptosauria. Mem. Am. Mus. Nat. Hist..

[27] Ezcurra MD (2016). The phylogenetic relationships of basal archosauromorphs, with an emphasis on the systematics of proterosuchian archosauriforms. PeerJ.

[28] Lydekker R (1885). Reptilia and amphibia of the maleri and denwa groups. Palaeontol. Indica.

[29] Langer MC, Schultz CL (2000). A new species of the late Triassic rhynchosaur *Hyperodapedon* from the Santa Maria Formation of south Brazil. Palaeontology.

[30] Huxley TH (1859). Postscript: Murchinson, R. I. On the sandstones of Morayshire (Elgin & c.) containing reptilian remains, and their relations to the Old Red Sandstone of that Country. Q. J. Geol. Soc. Lond..

[31] Sill WD (1970). *Schaphonyx sanjuanensis*, nuevo rincosaurio (Reptilia) de la Formación Ischigualasto, Triásico de San Juan Argentina. Ameghiniana.

[32] Chatterjee S (1974). A rhynchosaur from the Upper Triassic Maleri Formation of India. Philos. Trans. R. Soc. B.

[33] Mukherjee D, Ray S (2014). A new *Hyperodapedon* (Archosauromorpha, Rhynchosauria) from the upper Triassic of India: implications for rhynchosaur phylogeny. Palaeontology.

[34] Langer MC, Da Rosa AAS, Montefeltro FC (2017). *Supradapedon* revisited: geological explorations in the Triassic of southern Tanzania. PeerJ.

[35] Langer MC, Boniface M, Cuny G, Barbieri L (2000). The phylogenetic position of *Isalorhynchus genovefae*, a Late Triassic rhynchosaur from Madagascar. Ann. Paléontol..

[36] Raath MA, Oesterlen PM, Kitching JW (1992). First record of Triassic Rhynchosauria (Reptilia: Diapsida) from the lower Zambezi Valley Zimbabwe. Paleontol. Afr..

[37] Sues H-D, Olsen PE (2015). Stratigraphic and temporal context and faunal diversity of Permian-Jurassic continental tetrapod assemblages from the Fundy rift basin, eastern Canada. Atl. Geol..

[38] Gentil AR, Ezcurra MD (2018). Reconstruction of the masticatory apparatus of the holotype of the rhynchosaur *Hyperodapedon sanjuanensis* (Sill, 1970) from the Late Triassic of Argentina: implications for the diagnosis of the species. Ameghiniana.

[39] Benton MJ (1983). The Triassic reptile hyperodapedon from Elgin: functional morphology and relationships. Philos. Trans. R. Soc. Ser. B.

[40] Gentil AR, Ezcurra MD (2019). A new rhynchosaur maxillary tooth plate morphotype expands the disparity of the group in the Ischigualasto formation (Late Triassic) of Northwestern Argentina. Hist. Biol..

[41] Montefeltro FC, Langer MC, Schultz CL (2010). Cranial anatomy of a new genus of hyperodapedontine rhynchosaur (Diapsida, Archosauromorpha) from the upper Triassic of southern Brazil. Earth Environ. Sci. Trans. R. Soc. Edinb..

[42] Lucas SG, Heckert AB, Hotton N (2002). The rhynchosaur *Hyperodapedon* from the upper Triassic of Wyoming and its global biochronological significance. Bull. N. M. Mus. Nat. Hist. Sci..

[43] Benton MJ (1990). The species of *Rhynchosaurus*, a rhynchosaur (Reptilia, Diapsida) from the Middle Triassic of England. Philos. Trans. R. Soc. Ser. B.

[44] Langer MC, Montefeltro FC, Hone DWE, Whatley R, Schultz CL (2010). On *Fodonyx spenceri* and a new rhynchosaur from the Middle Triassic of Devon. J. Vertebr. Paleontol..

[45] Whatley, R. *Phylogenetic Relationship of Isalorhynchus Genovefae, the Rhynchosaur (Reptilia, Archosauromorpha) from Madagascar*. Ph.D. thesis, University of California, Santa Barbara (2005).

[46] Montefeltro FC, Bittencourt JS, Langer MC, Schultz CL (2013). Postcranial anatomy of the hyperodapedontine rhynchosaur *Teyumbaita sulcognathus* (Azevedo and Schultz, 1987) from the Late Triassic of southern Brazil. J. Vertebr. Paleontol..

[47] von Huene F (1938). *Stenaulorhynchus*, ein Rhynchosauride der ostafrikanischen Obertrias. Nova Acta Leopold..

[48] Carroll RL (1976). *Noteosuchus*—the oldest known rhynchosaur. Ann. S. Afr. Mus..

[49] Buffetaut E (1983). Isalorhynchus genovefae, n. g. n. sp. (Reptilia, Rhyncocephalia), um nouveau Rhyncosaure du Trias de Madagascar. Neues Jahrb. Geol. Paläontol. Monatshefe.

[50] Ezcurra MD, Montefeltro FC, Butler RJ (2016). The early evolution of rhynchosaurs. Front. Ecol. Evol..

[51] Schultz CL, Langer MC, Montefeltro FC (2016). A new rhynchosaur from south Brazil (Santa Maria Formation) and rhynchosaur diversity patterns across the Middle-Late Triassic boundary. Paläontolog. Z..

[52] Chatterjee S (1969). Rhynchosaurs in time and space. Proc. Geol. Soc. Lond..

[53] Gauthier JA, Kluge AG, Rowe T (1988). Amniote phylogeny and the importance of fossils. Cladistics.

[54] Sill WD (1967). *Proterochampsa barrionuevoi* and the early evolution of the Crocodilia. Bull. Mus. Comp. Zool..

[55] Trotteyn, M. J. *Revisión Osteológica, Análisis Filogenético y Paleoecología de Proterochampsidae (Reptilia-Arcosauriformes). *Ph.D. thesis*.* Universidad Nacional de Cuyo, Probiol, Mendoza (2011).

[56] Reig OA (1959). Primeros datos descriptivos sobre nuevos reptiles arcosaurios del Triásico de Ischigualasto (San Juan, Argentina). Rev. Asoc. Geol. Argent..

[57] Trotteyn MJ, Ezcurra MD (2014). Osteology of Pseudochampsa ischigualastensis gen. et. comb. nov. (Archosauriformes: Proterochampsidae) from the early Late Triassic Ischigualasto Formation of northwestern Argentina. PLoS ONE.

[58] Dilkes D, Arcucci A (2012). *Proterochampsa barrionuevoi* (Archosauriformes: Proterochampsia) from the Late Triassic (Carnian) of Argentina and a phylogenetic analysis of Proterochampsia. Palaeontology.

[59] Ezcurra MD, von Baczko MB, Trotteyn MJ, Desojo JB (2019). New proterochampsid specimens expand the morphological diversity of the rhadinosuchines of the Chañares Formation (Lower Carnian, Northwestern Argentina). Ameghiniana.

[60] Trotteyn MJ (2011). Material postcraneano de *Proterochampsa barrionuevoi* Reig 1959 (Diapsida: Archosauriformes) del Triásico Superior del centro-oeste de Argentina. Ameghiniana.

[61] Barberena MC (1982). Uma nova espécie de *Proterochampsa* (*P. nodosa*, sp. nov.) do Triássico do Brasil. An. Acad. Br. Ciênc..

[62] Cope ED (1869). Synopsis of the extinct Batrachia and Reptilia of North America. Trans. Am. Philos. Soc..

[63] Gauthier JA, Padian K, Hecht MK, Ostrom JH, Viohl G, Wellnhofer P (1985). Phylogenetic, functional, and aerodynamic analyses of the origin of birds and their flight. The Beginning of Birds.

[64] Zittel, K. A. *Handbuch der Paläontologie. Abteilung 1: Paläozoologie Band III Vertebrata (Pisces, Amphibia, Reptilia, Aves)* (R. Oldenbourg, Munich and Liepzig, 1887–1890).

[65] Krebs B (1974). Die archosaurier. Naturwissenschaften.

[66] Parrish JM (1993). Phylogeny of the Crocodylotarsi, with reference to archosaurian and crurotarsan monophyly. J. Vertebr. Paleontol..

[67] Wu XC, Chatterjee S (1993). *Dibothrosuchus elaphros*, a crocodylomorph from the Lower Jurassic of China and the phylogeny of the Sphenosuchia. J. Vertebr. Paleontol..

[68] Nesbitt SJ (2007). The anatomy of *Effigia okeeffeae* (Archosauria, Suchia), theropod-like convergence, and the distribution of related taxa. Bull. Am. Mus. Nat. Hist..

[69] Gower DJ, Nesbitt SJ (2006). The braincase of *Arizonasaurus babbitti*—further evidence for the non-monophyly of ‘rauisuchian’ archosaurs. J. Vertebr. Paleontol..

[70] Gower DJ (2002). Braincase evolution in suchian archosaurs (Reptilia: Diapsida): evidence from the rauisuchian *Batrachotomus kupferzellensis*. Zool. J. Linn. Soc..

[71] Nesbitt SJ (2011). The early evolution of archosaurs: relationships and the origin of major clades. Bull. Am. Mus. Nat. Hist..

[72] Ezcurra, M. D., Desojo, J. B. and Novas, F. E. A new medium-sized basal crocodylomorph with a lightly built axial skeleton from the Late Triassic Ischigualasto Formation, San Juan, Argentina. *Resúmenes del IV Congreso Latinoamericano de Paleontología de Verterbados*, Vol. 234 (2011).

[73] Nesbitt SJ (2005). Osteology of the Middle Triassic pseudosuchian archosaur *Arizonasaurus babbitti*. Hist. Biol..

[74] Nesbitt SJ, Butler RJ, Ezcurra MD, Barrett PM, Stocker MR, Angielczyk KD, Smith RMH, Sidor CA, Niedźwiedzki G, Sennikov AG, Charig AJ (2017). The earliest bird-line archosaurs and the assembly of the dinosaur body plan. Nature.

[75] Lecuona A, Ezcurra MD, Irmis RB (2016). Revision of the early crocodylomorph Trialestes romeri (Archosauria, Suchia) from the lower Upper Triassic Ischigualasto Formation of Argentina: one of the oldest-known crocodylomorphs. Pap. Palaeontol..

[76] Marsh OC (1884). The classification and affinities of dinosaurian reptiles. Nature.

[77] Parker WG (2007). Reassessment of the aetosaur ‘Desmatosuchus’ chamaensis with a reanalysis of the phylogeny of the Aetosauria (Archosauria: Pseudosuchia). J. Syst. Palaeontol..

[78] Casamiquela RM (1960). Notica preliminar sobre dos nuevos estagonolepoideos Argentinos. Ameghiniana.

[79] Currie BS, Colombi CE, Tabor NJ, Shipman TC, Montañez IP (2009). Stratigraphy and architecture of the Upper Triassic Ischigualasto Formation, Ischigualasto Provincial Park, San Juan, Argentina. J. S. Am. Earth Sci..

[80] Furin S, Preto N, Rigo M, Roghi G, Gianolla P, Crowley JL, Bowring SA (2006). High-precision U-Pb zircon age from the Triassic of Italy: Implications for the Triassic time scale and the Carnian origin of calcareous nannoplankton and dinosaurs. Geology.

[81] Ramezani J, Hoke GD, Fastovsky DE, Bowring SA, Therrien F, Dworkin SI, Atchley SC, Nordt LC (2011). High-precision U-Pb zircon geochronology of the Late Triassic Chinle Formation, Petrified Forest National Park (Arizona, USA): temporal constraints on the early evolution of dinosaurs. GSA Bull..

[82] Shipman, T. *Links between sediment accumulation rates and the development of alluvial architecture: Triassic Ischigualasto Formation, northwestern Argentina*. Ph.D. thesis, University of Arizona (2004).

[83] Kent DV, Malnis PS, Colombi CE, Alcober OA, Martínez RN (2014). Age constraints on the dispersal of dinosaurs in the Late Triassic from magnetochronology of the Los Colorados formation (Argentina). Proc. Natl. Acad. Sci..

[84] Tabor, N. J., Montañez, I. P., Kelso, K. A., Currie, B. S., Shipman, T. A. & Colombi, C. E. A Late Triassic soil catena: landscape and climate controls on paleosol morphology and chemistry across the Carnian-age Ischigualasto-Villa Union basin, northwestern Argentina. In: Alonso-Zarza, A.A. and Tanner, L.H. (eds.) *Paleoenvironmental Record and Applications of Calcretes and Palustrine Carbonates*. GSA Special Paper, Vol. 416, 17–42 (2006).

[85] Martin RE (1999). Taphonomy: A Process Approach.

[86] Colombi CE, Rogers RR, Alcober OA (2013). Vertebrate taphonomy of the Ischigualasto Formation. J. Vertebr. Paleontol..

[87] del Rey Á, Deckart K, Planavsky N, Arriagada C, Martínez F (2019). Tectonic evolution of the southwestern margin of Pangea and its global implications: evidence from the mid Permian-Triassic magmatism along the Chilean–Argentine border. Gondwana Res..

[88] Machuca BC, López MG, Morata D, Fuentes MG (2019). Geochemical constraints on the petrogenesis of Triassic alkaline basalts of Sierra de Valle Fertil, Western Sierras Pampeanas, Argentina: implications for their origin, evolution and tectonic setting. J. S. Am. Earth Sci..

[89] Monti M, Franzese JR (2019). Triassic continental oblique rifting controlled by Paleozoic structural grain: the Puesto Viejo Basin, western Argentina. J. S. Am. Earth Sci..

[90] Suárez RJ, Ghiglione MC, Calderón M, Sue C, Martinod J, Guillaume B, Rojo D (2019). The metamorphic rocks of the Nunatak Viedma in the Southern Patagonian Andes: provenance sources and implications for the early Mesozoic Patagonia–Antarctic Peninsula connection. J. S. Am. Earth Sci..

[91] Contreras VH (1997). El registro de rincosaurios en la Formación Ischigualasto (Argentina) y la evolución de los rincosaurios sudamericanos.

[92] Contreras, V.H. Rhynchosaurs from Ischigualasto Formation (Upper Triassic, Late Carnian), San Juan, Argentina. In *VII International Symposium of Mesozoic Terrestrial Ecosystems (Buenos Aires) Abstracts*, 18−19 (1999).

[93] Langer MC, Ramezani J, Da Rosa ÁAS (2018). U-Pb age constraints on dinosaur rise from south Brazil. Gondwana Res..

[94] Horn BLD, Melo T, Schultz CL, Philipp RP, Kloss HP, Goldberg K (2014). A new third-order sequence stratigraphic framework applied to the Triassic of the Paraná Basin, Rio Grande do Sul, Brazil, based on structural, stratigraphic and paleontological data. J. S. Am. Earth Sci..

[95] de Oliveira TV, Schultz CL (2007). La predominancia de *Exaeretodon* Cabrera 1943 en una sección Triásica de Brasil y su probable correlación con el mismo evento en la porción mediana superior de la Formación Ischigualasto (Triásico de Argentina). Ameghiniana.

[96] Langer MC, Ribeiro AM, Schultz CL, Ferigolo J (2007). The continental tetrapod-bearing Triassic of South Brazil. In Lucas, S.G. & Spielmann, J.A. (eds.). Glob. Triassic Bull N. M. Mus. Nat. Hist. Sci..

[97] Pretto FA, Schultz CL, Langer MC (2015). New dinosaur remains from the Late Triassic of southern Brazil (Candelária Sequence, *Hyperodapedon* Assemblage Zone). Alcheringa.

[98] Pretto FA, Langer MC, Schultz CL (2019). A new dinosaur (Saurischia: Sauropodomorpha) from the Late Triassic of Brazil provides insights on the evolution of sauropodomorph body plan. Zool. J. Linn. Soc..

[99] Müller RT, Garcia MS (2019). Rise of an empire: analysing the high diversity of the earliest sauropodomorph dinosaurs through distinct hypotheses. Hist. Biol..

[100] Holz M, Scherer CMS (2000). Sedimentological and paleontological evidence of paleoclimatic change during the South Brazilian Triassic: the register of a global trend towards a humid paleoclimate. Z. Geol. Paläontol..

[101] Pierini C, Mizusaki AMP, Scherer CMS, Alves DB (2002). Integrated stratigraphic and geochemical study of the Santa Maria and Caturrita formations (Triassic of the Paraná Basin), southern Brazil. J. S. Am. Earth Sci..

[102] Simms MJ, Ruffell AH (1989). Synchroneity of climatic change and extinctions in the Late Triassic. Geology.

[103] Roghi G, Gianolla P, Minarelli L, Pilati C, Preto N (2010). Palynological correlation of Carnian humid pulses throughout western Tethys. Palaeogeogr. Palaeoclimatol. Palaeoecol..

[104] Müller RT (2019). Craniomandibular osteology of *Macrocollum itaquii* (Dinosauria: Sauropodomorpha) from the Late Triassic of southern Brazil. J. Syst. Palaeontol..

[105] Kischlat EE, Lucas SG (2003). A phytosaur from the Upper Triassic of Brazil. J. Vertebr. Paleontol..

[106] Bittencourt JS, da Rosa ÁAS, Schultz CL, Langer MC (2012). Dinosaur remains from the ‘Botucaraí Hill’ (Caturrita Formation), Late Triassic of south Brazil, and their stratigraphic context. Hist. Biol..

[107] Agnolín FL, Rozadilla S (2017). Phylogenetic reassessment of Pisanosaurus mertii Casamiquela, 1967, a basal dinosauriform from the Late Triassic of Argentina. J. Syst. Paleontol..

[108] Baron MG, Norman DB, Barrett PM (2017). Baron et al. reply. Nature.

[109] Baron MG (2019). *Pisanosaurus mertii* and the Triassic ornithischian crisis: could phylogeny offer a solution?. Hist. Biol..

[110] Thulborn T (2006). On the tracks of the earliest dinosaurs: implications for the hypothesis of dinosaurian monophyly. Alcheringa.

[111] Irmis RB, Parker WG, Nesbitt SJ, Liu J (2007). Early ornithischian dinosaurs: the Triassic record. Hist. Biol..

[112] Novas FE (2009). The Age of Dinosaurs in South America.

[113] Olsen PE, Kent DV, Whiteside JH (2010). Implications of the Newark Supergroup-based astrochronology and geomagnetic polarity time scale (Newark-APTS) for the tempo and mode of the early diversification of the Dinosauria. Earth Environ. Sci. Trans. R. Soc. Edinb..

[114] Padian K (2012). The problem of dinosaur origins: integrating three approaches to the rise of Dinosauria. Earth Environ. Sci. Trans. R. Soc. Edinb..

[115] Langer MC, Ferigolo J (2013). The Late Triassic dinosauromorph *Sacisaurus agudoensis* (Caturrita Formation; Rio Grande do Sul, Brazil): anatomy and affinities. Geol. Soc. Lond. Spec. Publ..

[116] Sereno PC (2012). Taxonomy, morphology, masticatory function and phylogeny of heterodontosaurid dinosaurs. ZooKeys.

[117] Sereno PC, Novas FE (1994). The skull and neck of the basal theropod *Herrerasaurus ischigualastensis*. J. Vertebr. Paleontol..

[118] Martínez RN, Alcober OA (2009). A basal sauropodomorph (Dinosauria: Saurischia) from the Ischigualasto Formation (Triassic, Carnian) and the early evolution of Sauropodomorpha. PLoS ONE.

[119] Porro LB, Witmer LM, Barrett PM (2015). Digital preparation and osteology of the skull of *Lesothosaurus diagnosticus* (Ornithischia: Dinosauria). PeerJ.

[120] Nesbitt SJ, Langer MC, Ezcurra MD (2019). The anatomy of *Asilisaurus kongwe*, a Dinosauriform from the Lifua Member of the Manda Beds (~ Middle Triassic) of Africa. Anat. Rec..

[121] Dzik J, Sulej T (2007). A review of the early Late Triassic Krasiejów biota from Silesia Poland. Palaeontol. Pol..

[122] Sereno PC, Martínez RN, Alcober OA (2013). Osteology of *Eoraptor lunensis* (Dinosauria, Sauropodomorpha). J. Vertebr. Paleontol..

[123] Colbert EH (1981). A primitive ornithischian dinosaur from the Kayenta Formation of Arizona. Mus. North. Arizona Bull..

[124] Sereno PC (1991). *Lesothosaurus,* “fabrosaurids”, and the early evolution of Ornithischia. J. Vertebr. Paleontol..

[125] Kammerer CF, Nesbitt SJ, Shubin NH (2012). The first silesaurid dinosauriform from the Late Triassic of Morocco. Acta Palaeontol. Pol..

[126] Haubold H (1990). Ein neuer Dinosaurier (ornithischia, Thyreophora) aus dem unetren Jura des Nördlichen Mitteleuropa. Rev. Paleobiol..

[127] Butler RJ (2010). The anatomy of the basal ornithischian dinosaur *Eocursor parvus* from the lower Elliot Formation (Late Triassic) of South Africa. Zool. J. Linn. Soc..

[128] Norman DB (2019). *Scelidosaurus harrisonii* from the Early Jurassic of Dorset, England: cranial anatomy. Zool. J. Linn. Soc..

[129] Butler RJ, Porro LB, Galton PM, Chiappe LM (2012). Anatomy and cranial functional morphology of the small-bodied dinosaur *Fruitadens haagarorum* from the Upper Jurassic of the USA. PLoS ONE.

[130] Dzik J (2003). A beaked herbivorous archosaur with dinosaur affinities from the early Late Triassic of Poland. J. Vertebr. Paleontol..

[131] Norman DB, Sues H-D, Witmer LM, Coria RA, Weishampel DB, Dodson P, Osmólska H (2004). Basal ornithopoda. The Dinosauria.

[132] Nesbitt SJ, Sidor CA, Irmis RB, Angielczyk KD, Smith RM, Tsuji LA (2010). Ecologically distinct dinosaurian sister group shows early diversification of Ornithodira. Nature.

[133] Ezcurra ME, Nesbitt SJ, Fiorelli LE, Desojo JB (2020). New specimen sheds light on the anatomy and taxonomy of the Early Late Triassic dinosauriforms from the Chañares Formation NW Argentina. Anat. Rec..

[134] Bonaparte JF (1976). *Pisanosaurus mertii* Casamiquela and the origin of the Ornithischia. J. Paleontol..

[135] Baron MG, Norman DB, Barrett PM (2016). Postcranial anatomy of *Lesothosaurus diagnosticus* (Dinosauria: Ornithischia) from the Lower Jurassic of southern Africa: implications for basal ornithischian taxonomy and systematics. Zool. J. Linn. Soc..

[136] Piechowski R, Dzik J (2010). The axial skeleton of *Silesaurus opolensis*. J. Vertebr. Paleontol..

[137] Barrett PM, Butler RJ, Mundil R, Scheyer TM, Irmis RB, Sánchez-Villagra MR (2014). A palaeoequatorial ornithischian and new constraints on early dinosaur diversification. Proc. R. Soc. B Biol. Sci..

[138] Butler RJ (2005). The ‘fabrosaurid’ ornithischian dinosaurs of the upper Elliot Formation (Lower Jurassic) of South Africa and Lesotho. Zool. J. Linn. Soc..

[139] Langer MC (2003). The pelvic and hind limb anatomy of the stem-sauropodomorph *Saturnalia tupiniquim* (Late Triassic, Brazil). PaleoBios.

[140] Novas FE (1994). New information on the systematics and postcranial skeleton of *Herrerasaurus ischigualastensis* (Theropoda: Herrerasauridae) from the Ischigualasto Formation (Upper Triassic) of Argentina. J. Vertebr. Paleontol..

[141] Galton PM (2014). Notes on the postcranial anatomy of the heterodontosaurid dinosaur *Heterodontosaurus tucki*, a basal ornithischian from the Lower Jurassic of South Africa. Rev. Paléobiol..

[142] Langer MC, Benton MJ (2006). Early dinosaurs: a phylogenetic study. J. Syst. Paleontol..

[143] Novas FE (1989). The tibia and tarsus in Herrerasauridae (Dinosauria, incertae sedis) and the origin and evolution of the dinosaurian tarsus. J. Paleontol..

[144] Ezcurra MD, Brusatte SL (2011). Taxonomic and phylogenetic reassessment of the early neotheropod dinosaur *Camposaurus arizonensis* from the Late Triassic of North America. Palaeontology.

[145] Nesbitt SJ, Irmis RB, Parker WG, Smith ND, Turner AH, Rowe T (2009). Hindlimb osteology and distribution of basal dinosauromorphs from the Late Triassic of North America. J. Vertebr. Paleontol..

[146] Zerfass H, Lavina EL, Schultz CL, Garcia AGV, Faccini UF, Chemale F (2003). Sequence stratigraphy of continental Triassic strata of southernmost Brazil: a contribution to Southwestern Gondwana palaeogeography and palaeoclimate. Sed. Geol..

[147] Fiorelli LE, Ezcurra MD, Hechenleitner EM, Argañaraz E, Taborda JR, Trotteyn MJ, von Baczko MB, Desojo JB (2013). The oldest known communal latrines provide evidence of gregarism in Triassic megaherbivores. Sci. Rep..

[148] Haslett J, Parnell A (2008). A simple monotone process with application to radiocarbon-dated depth chronologies. J. R. Stat. Soc. Ser. C Appl. Stat..

[149] Parnell AC, Haslett J, Allen JRM, Buck CE, Huntley B (2008). A flexible approach to assessing synchroneity of past events using Bayesian reconstructions of sedimentation history. Quatern. Sci. Rev..

[150] R Core Team. *R: A Language and Environment for Statistical Computing*. R Foundation for Statistical Computing, Vienna. https://www.r-project.org/index.html. Accessed 27 February 2020 (2019).

