# Supplementary material for: The Late Triassic Ischigualasto Formation at Cerro Las Lajas (La Rioja, Argentina): fossil tetrapods, high-resolution chronostratigraphy, and faunal correlations
Source: Sci Rep. 2020 Jul 29;10:12782. doi: 10.1038/s41598-020-67854-1 (PMC7391656; doi:10.1038/s41598-020-67854-1)
Supplement: Supplementary file 1 — Supplementary file1 [file 41598_2020_67854_MOESM1_ESM.pdf]

SUPPLEMENTARY INFORMATION

The Late Triassic Ischigualasto Formation at Cerro Las Lajas (La Rioja, Argentina): fossil tetrapods, high-resolution chronostratigraphy, and faunal correlations

Julia B. Desojo*, Lucas E. Fiorelli, Martín D. Ezcurra, Agustín G. Martinelli, Jahandar Ramezani, Átila A. S. Da Rosa, M. Belén von Baczko, M. Jimena Trotteyn, Felipe C. Montefeltro, Miguel Ezpeleta & Max C. Langer

*To whom correspondence should be addressed. E-mail: julideso@fcnym.unlp.edu.ar

Contents:

i) Historical background and motivation

ii) Stratigraphy

ii.i) Lower section

ii.ii) Middle section

ii.iii) Upper section

iii) Rock petrography

iv) U-Pb geochronology

v) Table S1: List of collected specimens

vi) References

i) Historical background and motivation

The Hoyada del Cerro Las Lajas is traditionally known as the place where the holotype and only known specimen of *Pi. mertii* (PVL 2577), the putatively oldest-known ornithischian dinosaur (but see Agnolín & Rozadilla[1]), was found[2,3] (Fig. S1). Also, this locality provided the holotype of the ornithosuchid *V. rusconii*[4,5] and a specimen referred to the crocodylomorph *Trialestes romeri*[4,6-8]; the latter previously known from the Hoyada de Ischigualasto in the IPP, San Juan Province.

Casamiquela[2] published the holotype of *Pi. mertii* as the first material coming from a locality he referred to as “Agua de Las Catas”, located near kilometre 461 of the National Route 40. Later, Bonaparte[3] (p. 809) indicated its provenance as “Agua de Las Catas” or “Hoyada del Cerro Las Lajas” (Fig. S1), Lavalle Department, La Rioja Province, from the middle section of the Ischigualasto Formation. Mistakenly quoting Bonaparte[3], Agnolín & Rozadilla[1] stated that the specimen came from the uppermost levels of the Ischigualasto Formation. Martínez et al.[9] mentioned that *Pi. mertii* came from the area of Cerro Bola, but these authors possibly considered the Hoyada del Cerro Bola and the Hoyada del Cerro Las Lajas as the same set of outcrops. Note that the fig.1 in Colombi et al.[10] did not map the Cerro Las Lajas area, thus the Ischigualasto Formation has its northernmost figured outcrops at the Hoyada del Cerro Bola, which is ca. 12 kilometres to the south of the Hoyada del Cerro Las Lajas.

Bonaparte[4] (p. 670) stated that the holotype of *V. rusconii* (PVL 2578) was discovered in the middle third of the Ischigualasto Formation, in the vicinity of Cerro Las Lajas, and that it “has been found in levels with cynodonts and triassolestids”. Later, Bonaparte[11] (p. 367) supported this statement mentioning that both *Pi. mertii* and *V. rusconii* came from the “middle third of the Formation”. Martínez et al.[9] erroneously cited *V. rusconii* as coming from the IPP in San Juan Province.

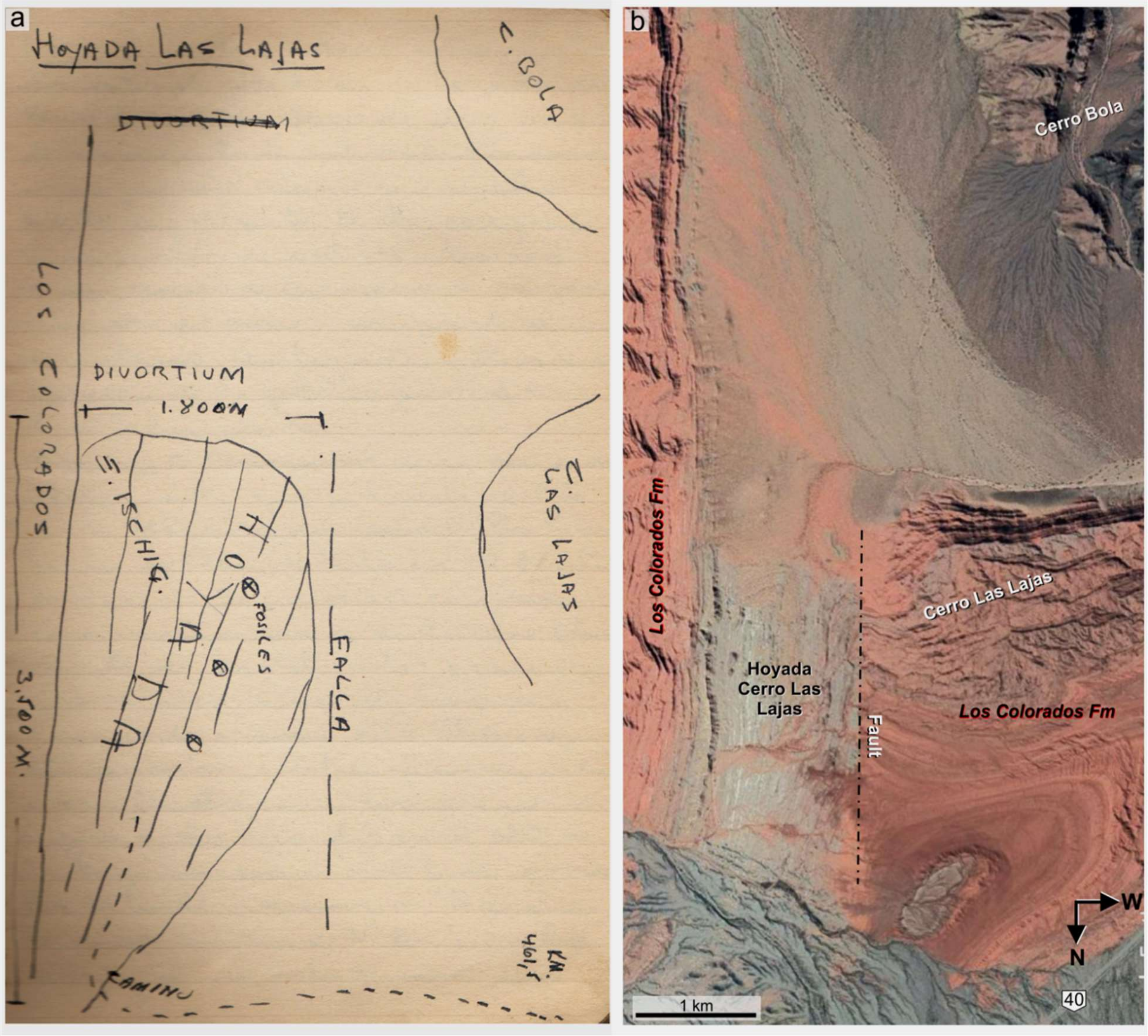

Fig. S1. Hoyada del Cerro Las Lajas study area. (a) Sketch map of the Hoyada del Cerro Las Lajas area from the field notes of José F. Bonaparte (May-July, 1962). **(b)** Satellite image of the area (from Google Maps). C. = hill (cerro); camino = pathway; divortium = watershed; E. Ischig = Ischigualasto strata; falla = fault; fosiles = fossils; hoyada = depression.

The referred specimen of *Tri. romeri* (PVL 3889) from the Hoyada del Cerro Las Lajas was first mentioned by Bonaparte[4,6,11-13] and subsequently studied by Lecuona et al.[8]. Bonaparte[4] mentioned *Tri. romeri* (at that time known as "*Triassolestes*" *romeri*) as coming from the "middle and lower thirds of the Ischigualasto Formation, San Juan and La Rioja

Provinces" and it was later reported that the corresponding specimen came from the vicinities of Cerro Las Lajas[12].

We had access to the field notes of Dr. José F. Bonaparte in which he reports his first findings at the Hoyada del Cerro Las Lajas. During May-July, 1962, José F. Bonaparte, Rafael Herbst, Galileo J. Scaglia, and Martín Vince carried out an expedition to the Palaeozoic Paganzo Series and the Triassic rocks of western Argentina. Twenty-two kilometres outside Villa Unión, in the direction of Jachal (along Route 40), they explored rocks of the Ischigualasto Formation at the Hoyada del Cerro Las Lajas.

Bonaparte wrote:

"*Encontramos una serie de restos fósiles, entre ellos principalmente restos de Rincosaurios y Cinodontes. De los primeros diversos restos de "picos", un fragmento de húmero y un fragmento de cráneo. De los segundos, el molde de la cavidad nasal y fronto-maxilar de un cráneo, un cráneo y mandíbula muy meteorizados que no extraímos (hay foto) asociado a una ulna. La parte inferior de lo aflorante de estos estratos posee un nivel con bochas, similares, pero en menor número que en Ischigualasto. El aspecto general de los mismos es algo distinto de lo que se vé en Ischigualasto."* (May 21, 1962).

"Por la mañana recorremos la parte inferior de la serie de la hoyada, localizando algunos fósiles, entre ellos unos restos pocos significativos." (May 22, 1962).

"*Por la mañana extraemos dos restos de fósiles en la hoyada del Cerro de Las Lajas. Uno de ellos comprende un conjunto de vértebras articuladas a un pequeño cráneo? que muestra dientes muy chiquitos. El otro comprende unos moldes de vértebras articuladas a un cráneo de arcosaurio. La parte inferior del cráneo comprende el molde interno. Ambos restos y otros más proceden de la parte (1/3) inferior de los E. de Ischigualasto*" (May 24, 1962).

[Translation: “We found a series of fossil remains, among them mainly remains of rhyncosaurs and cynodonts. Of the former ones various remains of ‘beaks’, a fragment of humerus, and a fragment of skull. Of the latter, the mould of the nasal cavity and the fronto-maxillary part of a skull, a highly weathered skull and jaw that we did not excavate (there is a picture) associated to an ulna. The lower part of the outcrop of these strata has a level with bowls, similar, but in smaller numbers than in Ischigualasto. Their general appearance is somewhat different from what is seen in Ischigualasto.” (May 21, 1962).

"In the morning we went through the lower part of the series of the *Hoyada*, finding some fossils, among them some insignificant remains.” (May 22, 1962).

“In the morning we extract two fossil remains in the *Hoyada* of *Cerro de Las Lajas*. One of them comprises a set of vertebrae articulated to a small skull?, which shows very small teeth. The other comprises moulds of vertebrae articulated to an archosaur skull. The lower part of the skull includes the internal mould. Both remains and others come from the lower part (1/3) of the Ischigualasto strata (May 24, 1962)”].

A few months ago, Bonaparte gave to one of the authors (A.G.M.) some fieldwork pictures in which he and his crew were collecting fossils at the Hoyada del Cerro Las Lajas in 1962 (Fig. S2). We were able to compare the landscape of that 1962 picture (Fig. S2a) with the current area we had been working (Fig. S2b) and confirm that this area was broadly the same where Bonaparte and crew collected the first fossil remains from Hoyada del Cerro Las Lajas. In particular, the fossil excavated by Bonaparte in the picture is located within the lower section of the Ischigualasto Formation, about 265 m above the base (see Stratigraphy section). Because we cannot determine which fossil material was being excavated in the 1962 picture, it does not help elucidating the exact stratigraphic position of historical taxa (e.g., *Pi. mertii*).

The comparison of Bonaparte’s field notes with published accounts by Reig[7] and Bonaparte[3,4,6,11-13] raises some critical issues. The two fossils mentioned in the note from

May 24th correspond to the holotypes of *Pi. mertii* and *V. rusconii*, respectively. The published sources state that they were collected in the middle section of the Ischigualasto Formation,

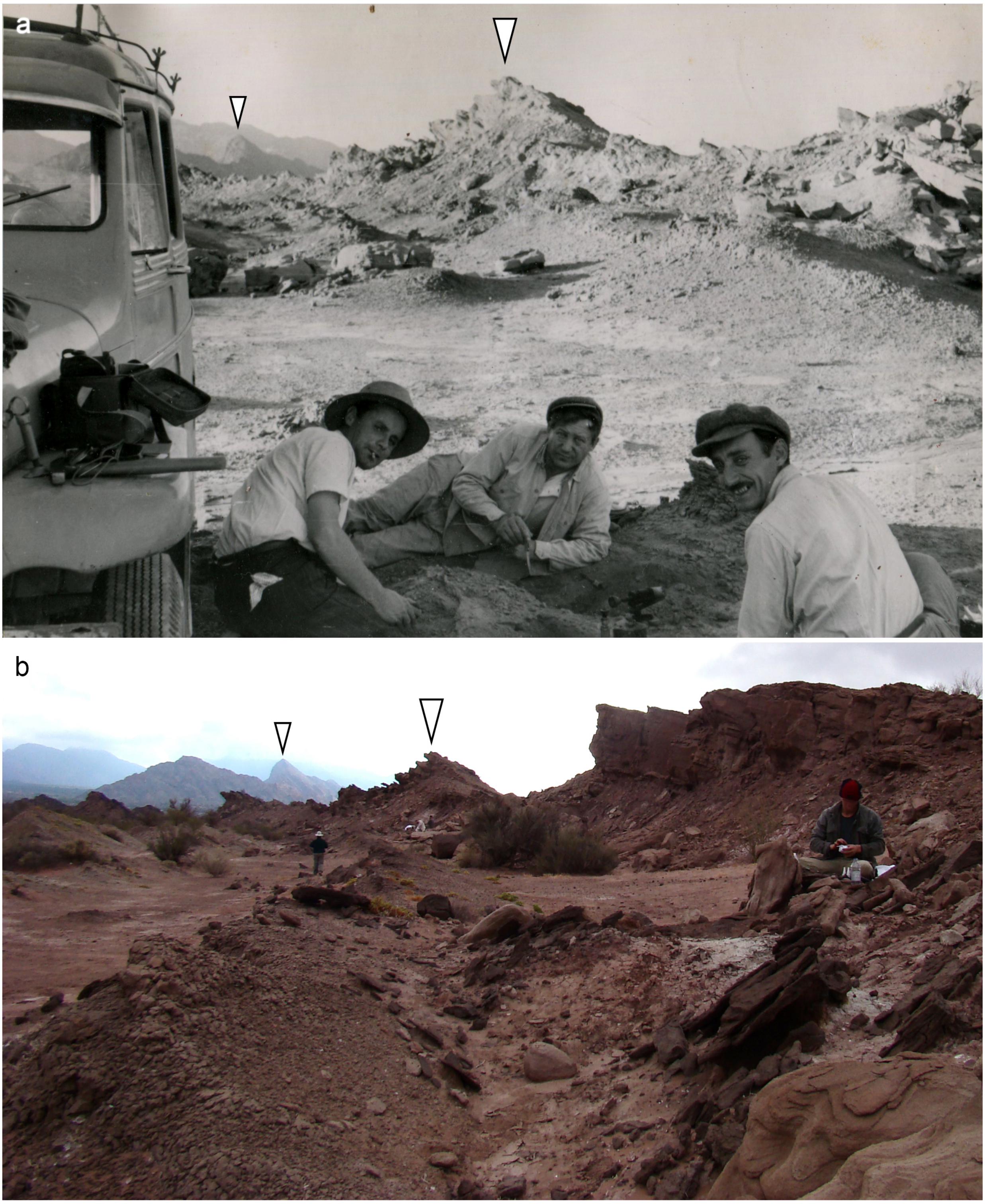

Fig. S2. Overview of the Ischigualasto Formation (~270 mab) at the Hoyada del Cerro Las Lajas. (a) (from left to right) José F. Bonaparte, Galileo J. Scaglia and Martin Vince during the excavation of fossil remains at the Hoyada de Cerro Las Lajas in the autumn of 1962. **(b)** The same place in 2016, during one of our fieldworks (taken by L.E.F.). The arrows point to the same peaks in the landscape.

but the field notes indicate the lower third of the unit as their provenances, and this is corroborated by the 1962 field work picture (Fig. S2). Bonaparte's sketch map of the Hoyada del Cerro Las Lajas (Fig. S1a) indicates the fossiliferous levels, but it is not detailed enough to further help in the discussion. In addition, Bonaparte's field notes (see also Reig[7]) indicate that they collected rhynchosaur and cynodont material in the Hoyada del Cerro Las Lajas, but these were never described. In the collections of Instituto Miguel Lillo (San Miguel de Tucumán, Argentina), where the fossil collected by Bonaparte at the time were deposited, rhynchosaurs and cynodonts from the Ischigualasto Formation are either mentioned as coming from the San Juan Province or no geographic provenance is provided. It is possible, therefore, that some of the material actually came from the La Rioja province, but this information was omitted from the collection records, which at that time focussed on their stratigraphic rather than geographical origins.

Subsequently, the Cerro Las Lajas area was not systematically explored, but it was revisited by José F. Bonaparte and his crew in the eighties, and also by other teams from the MACN, the Universidad Nacional de San Juan (Argentina), and the University of Chicago (USA). Particularly, Sereno[14] mentioned that an "Argentine-American" team revisited the "Agua de Las Catas" site in 1991, which "was not particularly fossiliferous and did not yield additional dinosaurian remains". Indeed, only the fossils collected during the initial fieldworks by José F. Bonaparte were employed in further studies of the Hoyada del Cerro Las Lajas tetrapods.

At the end of May 2013, our five-member team conducted a five-day exploratory field trip to the area, where the fossiliferous beds in question were identified, yielding fossils such as rhynchosaurs and the cynodont genus *Exaeretodon*. The promising results provided impetus for more comprehensive research projects, funded by the South American

agencies ANPCyT (Argentina) and FAPESP (Brazil), to fully explore the Cerro Las Lajas area. Three extended field excursions were conducted during April-May of 2016, 2017, and 2019, each with over ten participants, including palaeontologists and geologists of diverse expertise. These resulted in the collection of over 100 new fossil specimens within a high-resolution framework of measured stratigraphic sections, as well as in identification and sampling of volcanic beds for radioisotopic age determination. The results provided a more accurate chronostratigraphic context for the invaluable fossils collected in the sixties, as well as for the entire Ischigualasto Formation fauna, allowing for their more precise correlation to coeval assemblages.

ii) Stratigraphy

Vast exposures together with an overall lack of structural complexity allowed stratigraphic measurements to be made along a single, near-straight, W-E transect across the Hoyada del Cerro Las Lajas (Fig. 1 and Fig. S1b). Stratigraphic thicknesses were measured using a reel tape measure perpendicular to the bed strikes and corrected for measured bed dips. GPS coordinates were recorded for outcrops, lithologic features, strike-dip recordings, fossil localities and sampled tuff beds in the area. A single stratigraphic column was constructed (Fig. 2) for 1059 meters of the Ischigualasto Formation exposed at Las Lajas incorporating the exact positions of all stratigraphic horizons of interest. The formation is subdivided into three sections based on lithologic characteristics and depositional facies, as follows.

ii.i) Lower section. In the Hoyada del Cerro Las Lajas (Figs. 2-3 and Fig. S3), the studied section starts with a few meters of light green sandstones (5G 6/6, Munsell scale) and interlayered dark green mudstones (5G 5/6) of the deltaic to lacustrine deposits of the Los Rastros Formation (Fig. S3a). Sandstones form centimetre-scale, tabular or locally

channelized beds, with unclear stratification. Their apparent subvertical dips follow the major fault at the base of the succession.

The lower section of the Ischigualasto Formation (Figs. 2-3 and Fig. S3a-b) is 310 meters-thick and is characterized by an alternation of laminated to massive siltstones (moderate brown 5YR 4/4) (Fig. S3b), ledge-forming conglomerates and coarse sandstones (light- brown 10YR 8/6) with planar and trough cross-bedding, and less abundant medium- to fine-grained sandstones with trough cross-bedding (light green 5G 6/6). Sandstones occur as mm- to cm-scale, tabular or channelized beds, whereas normal-graded conglomerates form amalgamated channel bodies with 0.2 to 5 m thickness and more than 100 m of lateral extension. Tuffaceous deposits occur (Fig. S3a-b) as cm-scale, greyish-green, clay-rich (bentonitic) beds interlayered with siltstones and mudstones.

Extraformational clasts smaller than 8 cm are found exclusively at the base of the channels (lag). The basal Ischigualasto strata also contain intraclasts of the underlying Los Rastros Formation. Fine-grained lithologies are often massive, with salt crystals and/or gypsum layers as fracture infilling. Other post-depositional modifications include pedogenetic root traces and phreatic hydromorphism, marked by discoloration (mottling) with lateral persistence. This facies association is interpreted as a meandering fluvial depositional setting where the proportion of channels to floodplains is in the range of 1:2 to 1:5. The shallowing-upward, basal succession of the Ischigualasto Formation suggest a relative decrease in accommodation space, resulting from a prograding normal regression[15]. Colombi *et al.*[16] interpreted such amalgamated channel belts as a temporal adjustment of the fluvial system to the equilibrium profile (aggradation under low accommodation) after an incision surface in the centre of the basin, which suggests a fall in the lacustrine level. This interval can be interpreted as a delayed outcome of the preceding Carnian Pluvial

Event[17-21], which supposedly took place during the deposition of the Los Rastros Formation.

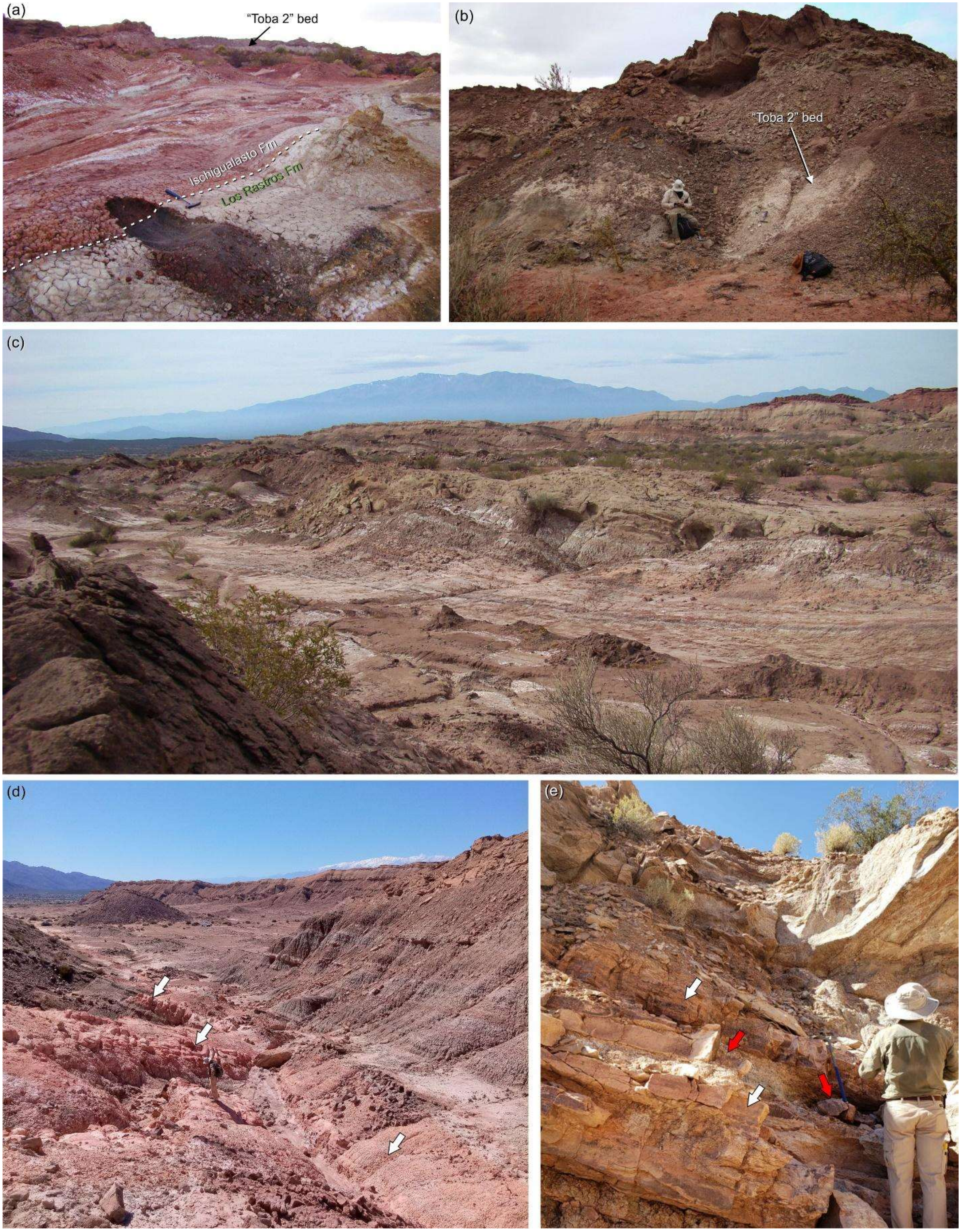

Fig. S3. Ischigualasto Formation in the Hoyada del Cerro Las Lajas locality. (a) Boundary between the olive Los Rastros and red-brown Ischigualasto formations (dotted line); arrow indicates the Toba-2 for U-Pb analysis. **(b)** Close-up to the "Toba-2" level at ca. 107 mab. **(c)** Panoramic of the typical levels of *Hyperodapedon* biozone ca. 200-250 mab, with the Sierra de Famatina (top. elevation: 6,250 masl). **(d)** Upper section of the Ischigualasto Formation in the Hoyada del Cerro Las Lajas showing an 8 m-thick, pinkish-buff, ignimbrite bed at ca. 800 m above base –arrows–. **(e)** Multiple interbedded bentonite levels –arrows– near 40 m below the top of the Ischigualasto Formation; red arrow indicates ignimbrite Sample LL041219-6.

At 107 m above base, a prominent tuff bed up to 2 m in thickness occurs at the base of a ca. 1.5 m-thick sandstone ledge (Fig. S3b). This bed is a white, matrix-supported, crystal-rich tuff, which is distinct from all other tuffaceous (bentonitic) interbeds of the lower Ischigualasto Formation and is herein labelled "Toba-2" (Fig. S3a-b). The documented fossil record of the Ischigualasto Formation at Las Lajas begins immediately above the "Toba-2" level (Fig. S3c). Based on its distinct lithology and stratigraphic position, we consider Toba-2 as a direct correlative of the Herr Toba tuff[9,22] exposed in the lower Ischigualasto Formation at IPP.

ii.ii) Middle section. The middle Ischigualasto Formation at Las Lajas (310–740 m above base) marks a transition from amalgamated to isolated/sinuous channels, along with preservation of thick floodplain deposits (Fig. S3c). Channel bodies are less than 1m in thickness and have more than 100 m of lateral extension. These meandering fluvial deposits shows a channel to floodplain proportion of <1:10, suggesting higher accommodation and a regional rise in the base level. The middle section also marks the first occurrence of pinkish-white, welded tuff (ignimbrite) beds, with thicknesses of less than 1m in this part of the succession. Increased volcanic activity would produce episodic increases in aggradation with the vertical growth of the alluvial plains[16], resulting in fine-grained floodplain deposits, paleosols and hydromorphic discoloration. The change from calcic to argillic paleosols suggests elevated humidity during this interval, similar to that recognized in the area of the IPP[23,24].

ii.iii) Upper section. The upper interval of the Ischigualasto Formation (740-1070 m above base) is dominated by successive amalgamated channels, similar to those described in the lower section, which are interbedded with fine-grained floodplain deposits. The individual channels are typically less than 1m-thick, while the amalgamated channels bodies exceed more than 5 m in thickness. The proportion of channels to floodplains gradually increases from 1:5 to 10:1, along with an upward decrease in channel incision. This upward-thickening trend of channels is accompanied by an increase in average clast size (15 cm maximum) that represents a rise in the flow energy of the fluvial system. The changes in grain-size, thickness, shape and frequency of channels suggest transition from a meandering to a braided fluvial system, which is the likely result of decrease in accommodation space.

The upper section marks a significant increase in the volcanic component of the Ischigualasto Formation (Fig. S3d-e). While bentonite and tuffaceous sandstone interlayers continue to occur throughout the formation, there is a notable increase in the abundance and thickness of welded tuffs (ignimbrites) in the upper section (Fig. S3e), culminating in a ca. 8 m-thick, pinkish-buff, ledge-forming ignimbrite flow at 800 m above base (Fig. S3d). The latter suggests a volcanic source proximal to the basin. Multiple interbedded bentonites (recessed) and welded tuffs (ledge-forming) occur near the top of the Ischigualasto Formation, ca. 40 m below the Los Colorados Formation contact (Figs. 2-3). The uppermost 30 m of the upper section, as well as the overlying Los Colorados Formation are essentially free of volcanic beds.

iii) Rock petrography

Despite the great significance of the Las Lajas fossils recovered during the 1960's expeditions, their exact positions with respect to our new chronostratigraphic framework

remain ambiguous due to inadequate field information. In order to infer the stratigraphic provenance of these fossils, we carried out petrographic analyses of the rock matrix in which the holotypes of *Pi. mertii* and *V. rusconii* (Fig. S4) were preserved and compared them to those of selected samples collected during our field works.

The holotype of *Pi. mertii* (PVL 2577) is preserved in a coarse siltstone to very fine sandstone consisting of near-equal proportions of clasts and matrix (Fig. S4a). Its clast mineralogy reflects a quartz-arenite, including 95% quartz, 2% lithic clasts, < 5% volcanic glass and ~1% plagioclase. The clasts measure between 0.05 mm to 0.3 mm, with an average of ~0.15mm. They are very angular to subrounded and poorly sorted. The matrix consists of an opaque cement with a high iron oxide content and no detectable carbonate. The holotype of *V. rusconii* (PVL 2578) is preserved in a matrix-supported, siltstone to very fine-grained sandstone with only ~30% clasts (Fig. S4d). It has the clast mineralogy of a greywacke, with 95% quartz, 10% lithic fragments, ~5% volcanic glass, and ~1% feldspar (plagioclase) and minor micas. The clasts measure between 0.001 mm to 0.10 mm, averaging ~0.03mm. They are subangular to rounded, and moderately sorted (0.5 phi). As in the *Pi. mertii* sample, the matrix is a very opaque cement, but despite considerable iron oxide content, it is mostly formed by an amorphous carbonate cement.

In comparison, the petrographic characteristics of the bone bed of an indeterminate tetrapod fossil fragment (field number JT 02-2016) from about 120 m above base (a short distance above the "Toba 2" tuff) reveals a quartz-arenite with dark iron oxide matrix (Fig. S4b), very similar to the matrix of *Pi. mertii*. Similarly, the bone bed of the rhynchosaur fossil (CRILAR-Pv 658) from 270 m above base (main fossil zone; Fig. S4e) has the characteristics of a greywacke with a very opaque matrix and "pseudoclasts" composed of bone fragments, which is lithologically very similar to the matrix of *V. rusconii*. A third sample taken from a rhynchosaur bone bed (CRILAR-Pv 654) and from the same level as

JT 02-2016 is a fine greywacke (~50% clasts, 0.1 to 0.5 mm in size) composed of a variety of clast sizes and shapes (subangular, poorly sorted) and abundant volcanic glass. The opaque matrix is a mixture of iron oxide, siliceous and carbonate cement.

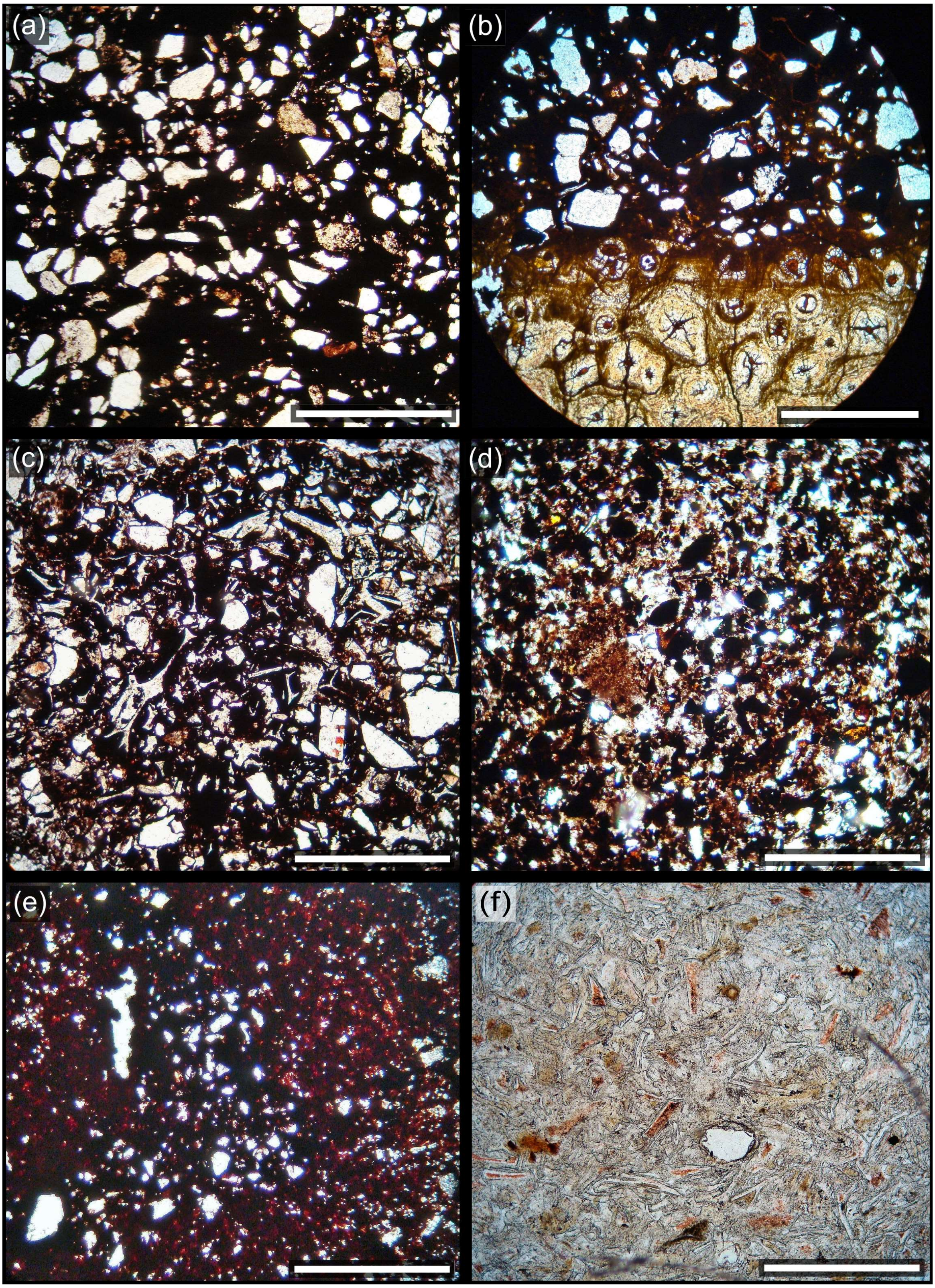

Fig. S4. Thin-section photomicrographs (plane-polarized light) of sedimentary rock samples from the Ischigualasto Formation at the Hoyada del Cerro Las Lajas. (a) Rock matrix of the Pisanosaurus fossil (PVL 2577). **(b)** Rock matrix of an Hyperodapedon sp. fossil (CRILAR-Pv 656), showing the bone structure in the lower part, from ca. 100-130 mab. **(c)** Tuffaceous siltstone (field number BVB 09-2016) with abundant volcanic glass shards from 120 mab. **(d)** Rock matrix of the Venaticosuchus fossil (PVL 2578). **(e)** Rock sample from 270 mab. **(f)** Thin-section of the welded tuff from ca. 1,020 mab (the same for sample LL041219-2). Scale bars: 0.5 mm.

The comparison of samples collected in this study to the matrix rocks from the historical findings suggests that *Pi. mertii* was collected from brownish mudstones that occur throughout the section, but are most common near its base. Because *Pi. mertii* was preserved within a concretion with iron oxide matrix, we speculate that it came from a level between 110 and 180 m above base, characterized by "dark-reddish" concretions and high concentration of iron oxides. This level is below the main fossil zone at 240–300 m above base, but generally matches records of *Hyperodapedon*, *Aetosauroides*, and *Exaeretodon*. In contrast, the matrix of *V. rusconii* matches thin-bedded siltstones typical of upper levels of the main fossil zone, hence occurring conceivably together with *Teyumbaita*, *Proterochampsa*, and *Exaeretodon*. If we follow Bonaparte (p. 367)[11] that the referred specimen of *Tri. romeri* was found at the same levels as *V. rusconii*, we can add this crocodylomorph to the faunal list of the lower third of the Ischigualasto Formation at Las Lajas (see also Historical Background).

iv) U-Pb geochronology

Samples of tuff collected from the Ischigualasto Formation at Hoyada del Cerro Las Lajas range from ledge-forming welded tuffs (ignimbrites) to geomorphologically recessed bentonites. Twenty one single zircons from 3 tuff samples were analyzed by the U-Pb CA-ID-TIMS method at the Massachusetts Institute of Technology Isotope Laboratory, following the general procedures described in Ramezani *et al.*[25]. The welded tuffs were processed by standard crushing and pulverizing techniques, whereas the bentonites were liquefied in water for 48 hours and disaggregated in a blender, followed by removal of clay components using an ultrasonic dismembrator device[26]. Heavy mineral concentrations were obtained using a Frantz® magnetic separator and high-density liquids. Zircons were hand selected under a binocular microscope and pretreated by a chemical abrasion technique

modified after Mattinson[27], which involved thermal annealing in a furnace at 900°C for 60 hours, followed by partial dissolution in 29M HF at 210°C in high-pressure vessels for 12 hours. The chemically abraded grains were fluxed in several hundred microliters of dilute HNO_3 and 6M HCl, successively on a hot plate and in an ultrasonic bath (1 hour each), and rinsed with several volumes of Millipore water in between to remove the leachates.

All zircon grains were spiked with the EARTHTIME ET535 mixed ^{205}Pb-^{233}U-^{235}U isotopic tracer[28,29] prior to complete dissolution in 29M HF at 210°C for 48 hours and subsequent Pb and U purification via an HCl-based anion-exchange column chemistry[30]. Pb and U were loaded together onto single outgassed Re filaments along with a silica-gel emitter solution and their isotopic ratios were measured on an Isotopx X62 multi-collector thermal ionization mass spectrometer equipped with a Daly photomultiplier ion-counting system at MIT. Pb isotopes were measured as mono-atomic ions in a peak-hopping mode on the ion-counter and were corrected for a mass-dependent isotope fractionation of 0.18% ± 0.05% per atomic mass unit (2σ). U isotopes were measured as dioxide ions in a static mode using three Faraday collectors, while subjected to a within-run mass fractionation correction using the $^{233}U/^{235}U$ ratio of the tracer and a predicted sample $^{238}U/^{235}U$ ratio of 137.818 ± 0.045[31], as well as an oxide correction based on an independently determined $^{18}O/^{16}O$ ratio of 0.00205 ± 0.00005.

Complete Pb and U isotopic data are given in Supplementary Materials Table S1 and the age results are illustrated on the age plots of Figure 4. Data reduction, calculation of dates and propagation of uncertainties used the Tripoli and ET_Redux applications and algorithms[32,33]. The individual $^{206}Pb/^{238}U$ dates were corrected for initial ^{230}Th disequilibrium based on a magma Th/U ratio of 2.8 ± 1.0 (2σ). The sample age is calculated based on the weighted mean $^{206}Pb/^{238}U$ date of the youngest statistically coherent population of analyses from each sample that overlap within 2σ analytical uncertainty, after

excluding older analyses interpreted as detrital or xenocrystic. A set of high-precision tuff dates that are mutually resolvable outside internal uncertainties and obey superposition serve as reliable approximations for the respective sediment deposition ages. Calculated sample dates along with their 95% confidence level uncertainties are reported in Table S1 and follow the notation ±*X*/*Y*/*Z* Ma, where *X* is the internal (analytical) uncertainty in the absence of all external errors, *Y* incorporates the U-Pb tracer calibration error, and *Z* includes the latter as well as the U decay constant errors of Jaffey *et al.*[34]. *Z* must be taken into account when dates from different chronometers (e.g., U-Pb of this study versus $^{40}Ar/^{39}Ar$ of Rogers *et al.*[22]) are compared.

The ages of stratigraphic levels of interest (e.g., fossiliferous intervals) were interpolated by constructing a Bayesian age-depth model using the Bchron software package[35,36], written for R (R Core Team 2019)[37], and by utilizing the weighted mean dates of all 3 dated ash beds and their stratigraphic positions. The underlying Markov chain Monte Carlo algorithm of Bchron accounts for possible variations in sediment accumulation rate (or unconformities) between the dated stratigraphic levels and thus produces more objective age uncertainties than the conventional linear extrapolation or spline-fit calculation methods. The extrapolated stratigraphic ages are reported with 95% confidence interval in the text.

Sample Toba-2 – This sample is from the 'Toba-2 tuff', a ~2 m-thick, cliff-forming tuff that occurs 107 m above base of the section (Figs. 2-3 and Fig. S3a-b). Six single zircons analyzed from this sample define a coherent cluster without any outliers and yield a weighted mean $^{206}Pb/^{238}U$ date of 229.25 ± 0.10/0.16/0.30 Ma with a MSWD of 1.2 (Fig. 4; Table S1). This date directly constrains the eruption age of the tuff in the lower section of the Ischigualasto Formation.

Sample LL041219-2 – This key bentonitic sample occurs 160 m above base of the section (Fig. 3) and below the main fossil zone of the Ischigualasto Formation (lower section). Eight single zircons analyzed from this sample range in $^{206}Pb/^{238}U$ date from 279.01 ± 0.33 Ma to 228.72 ± 0.31 Ma, with the youngest 4 analyses defining a coherent cluster with a weighted mean date of 228.97 ± 0.22/0.23/0.33 Ma (MSWD = 1.9). The latter serves as a maximum depositional age for the tuffaceous sediment.

Sample LL041219-6 – This welded tuff (ignimbrite) occurs 1,035 m above base of the section (upper section) and 35 meters below the first fluvial sandstone of the overlying Los Colorados Formation (Figs. 2-3 and Fig. S3e). Seven analyzed zircons from this sample produced $^{206}Pb/^{238}U$ dates from 226.77 ± 0.55 Ma to 221.77 ± 0.40 Ma, while the youngest 4 analyses overlap well with uncertainty and produce a weighted mean date of 221.82 ± 0.10/0.12/0.27 Ma (MSWD = 0.63). This date provides a reliable (maximum) depositional age for the upper Ischigualasto Formation at Las Lajas.

v) Table S1. *List of collected specimens*

Collection number	Higher level taxonomic assignment	Lower level taxonomic assignment	GPS coordinates
CRILAR-Pv 579	Proterochampsidae	*Proterochampsa barrinonuevoi*	29º28'49.8"S - 68º20'54.6"W
CRILAR-Pv 580	Aetosauria	*Aetosauroides scagliai*	29º28'18.2"S - 68º20'56.7"W
CRILAR-Pv 581	Pseudosuchia	Paracrocodylomorpha indet.	29º28'14.3"S - 68º20'52.7"W
CRILAR-Pv 582	Rhynchosauria	*Hyperodapedon* sp.	no data
CRILAR-Pv 583	Rhynchosauria	*Hyperodapedon sanjuanensis*	29º28'33.2"S - 68º20'54.8"W
CRILAR-Pv 584	Rhynchosauria	*Hyperodapedon sanjuanensis*	29º28'46.2"S - 68º20'58.5"W
CRILAR-Pv 585	Rhynchosauria	*Hyperodapedon* sp. nov.	29º28'13.0"S - 68º20'54.9"W
CRILAR-Pv 586	Rhynchosauria	*Teyumbaita* sp. nov.	29º28'25.9"S - 68º20'54.2"W
CRILAR-Pv 587	Rhynchosauria	*Teyumbaita* sp. nov.	29º28'50.3"S - 68º20'56.8"W
CRILAR-Pv 588	Rhynchosauria	*Teyumbaita* sp. nov.	29º28'50.1"S - 68º20'54.2"W
CRILAR-Pv 589	Rhynchosauria	*Teyumbaita* sp.	29º28'50.1"S - 68º20'54.2"W
CRILAR-Pv 590	Rhynchosauria	*Teyumbaita* sp.	29º28'50.1"S - 68º20'54.2"W
CRILAR-Pv 591	Rhynchosauria	*Teyumbaita* sp.	29º28'50.1"S - 68º20'54.2"W
CRILAR-Pv 592	Rhynchosauria	*Teyumbaita* sp.	29º28'26.4"S - 68º20'54.4"W
CRILAR-Pv 593	Rhynchosauria	*Teyumbaita* sp.	29º28'50.1"S - 68º20'54.2"W
CRILAR-Pv 594	Rhynchosauria	*Teyumbaita* sp.	29º28'25.5"S - 68º20'54.1"W
CRILAR-Pv 595	Rhynchosauria	*Teyumbaita* sp. nov.	29º28'45.3"S - 68º20'53.8"W
CRILAR-Pv 596	Rhynchosauria	*Teyumbaita* sp.	29º28'56.7"S - 68º20'57.2"W
CRILAR-Pv 597	Rhynchosauria	*Teyumbaita* sp.	29º28'47.6"S - 68º20'52.9"W
CRILAR-Pv 598	Rhynchosauria	*Teyumbaita* sp.	29º28'48.3"S - 68º20'54.5"W
CRILAR-Pv 600	Rhynchosauria	Rhynchosauridae indet.	29º28'50.1"S - 68º20'55.1"W
CRILAR-Pv 640	Cynodontia	*Exaeretodon* sp.	29º28'26.2"S - 68º20'54.9"W
CRILAR-Pv 641	Rhynchosauria	Rhynchosauridae indet.	29º28'28.4"S - 68º20'54.3"W
CRILAR-Pv 642	Rhynchosauria	*Teyumbaita* sp. nov.	29º28'47.5"S - 68º20'51.9"W
CRILAR-Pv	Rhynchosauria	*Teyumbaita* sp. nov.	29º28'21.6"S -

643			68º20'54.3"W
CRILAR-Pv 644	Cynodontia	*Exaeretodon* sp.	29º28'47.3"S - 68º20'49.3"W
CRILAR-Pv 645	Rhynchosauria	*Teyumbaita* sp. nov.	29º28'45.3"S - 68º20'53.8"W
CRILAR-Pv 646	Rhynchosauria	*Hyperodapedon sanjuanensis*	29º28'40.0"S - 68º20'57.9"W
CRILAR-Pv 647	Cynodontia	*Exaeretodon* sp.	29º28'25.9"S - 68º20'54.5"W
CRILAR-Pv 649	Cynodontia	*Exaeretodon* sp.	29º28'47.5"S - 68º20'51.9"W
CRILAR-Pv 650	Rhynchosauria	*Hyperodapedon sanjuanensis*	29º28'35.2"S - 68º20'54.1"W
CRILAR-Pv 651	Rhynchosauria	*Teyumbaita* sp. nov.	29º28'24.6"S - 68º20'53.9"W
CRILAR-Pv 652	Rhynchosauria	indet.	29º28'22.2"S - 68º20'53.7"W
CRILAR-Pv 653	Rhynchosauria	indet.	29º28'46.3"S - 68º20'54.2"W
CRILAR-Pv 654	Rhynchosauria	indet.	29º28'12.4"S - 68º20'54.4"W
CRILAR-Pv 655	Rhynchosauria	indet.	29º28'18.8"S - 68º20'56.4"W
CRILAR-Pv 656	Rhynchosauria	indet.	no data
CRILAR-Pv 657	Rhynchosauria	indet.	no data
CRILAR-Pv 658	Rhynchosauria	indet.	29º28'23.7"S - 68º20'54.6"W
CRILAR-Pv 659	Rhynchosauria	indet.	29º28'35.1"S - 68º20'54.5"W
CRILAR-Pv 660	Rhynchosauria	indet.	29º28'37.7"S - 68º20'54.3"W
CRILAR-Pv 661	Rhynchosauria	indet.	29º28'18.2"S - 68º20'53.5"W
CRILAR-Pv 662	Rhynchosauria	indet.	29º28'39.4"S - 68º20'48.9"W
CRILAR-Pv 663	Cynodontia	*Exaeretodon* sp.	29º28'51.2"S - 68º20'57.2"W
CRILAR-Pv 664	Rhynchosauria	indet.	29º28'23.8"S - 68º20'54.0"W
CRILAR-Pv 665	Rhynchosauria	indet.	no data
CRILAR-Pv 666	Rhynchosauria	indet.	no data
CRILAR-Pv 667	Rhynchosauria	indet.	29º28'48.0"S - 68º20'55.1"W
CRILAR-Pv 668	Rhynchosauria	indet.	29º28'26.2"S - 68º20'54.1"W
CRILAR-Pv 670	Rhynchosauria	indet.	29º28'22.5"S - 68º20'54.3"W
CRILAR-Pv 671	Rhynchosauria	indet.	29º28'28.8"S - 68º20'54.9"W

CRILAR-Pv 672	Cynodontia	*Exaeretodon* sp.	29º28'14.6"S - 68º20'54.9"W
CRILAR-Pv 673	Rhynchosauria	indet.	29º28'51.5"S - 68º20'52.4"W
CRILAR-Pv 674	Rhynchosauria	indet.	29º28'25.4"S - 68º20'54.8"W
CRILAR-Pv 675	Rhynchosauria	indet.	29º28'31.9"S - 68º20'54.7"W
CRILAR-Pv 676	Rhynchosauria	indet.	29º28'15.3"S - 68º20'52.7"W
CRILAR-Pv 677	Rhynchosauria	indet.	29º28'14.6"S - 68º20'52.3"W
CRILAR-Pv 678	Cynodontia	indet.	29º28'14.6"S - 68º20'52.3"W
CRILAR-Pv 680	Rhynchosauria	indet.	29º28'50.6"S - 68º20'54.3"W
CRILAR-Pv 681	Rhynchosauria	indet.	29º28'55.5"S - 68º20'57.2"W
CRILAR-Pv 683	Rhynchosauria	indet.	29º28'46.9"S - 68º20'55.5"W
CRILAR-Pv 684	Rhynchosauria	indet.	29º28'14.7"S - 68º20'55.8"W
CRILAR-Pv 685	Rhynchosauria	indet.	no data
CRILAR-Pv 686	Rhynchosauria	indet.	29º28'17.2"S - 68º20'53.3"W
CRILAR-Pv 687	Rhynchosauria	indet.	29º28'30.9"S - 68º20'56.1"W
CRILAR-Pv 688	Archosauromorpha	indet.	29º28'24.6"S - 68º20'53.9"W
CRILAR-Pv 689	Rhynchosauria	indet.	29º28'09.5"S - 68º20'52.8"W
CRILAR-Pv 690	Rhynchosauria	indet.	29º28'20.2"S - 68º20'54.1"W
CRILAR-Pv 691	Rhynchosauria	indet.	29º28'45.0"S - 68°20'53.7"W

vi) References

1. Agnolín, F. L. & Rozadilla, S. Phylogenetic reassessment of *Pisanosaurus mertii* Casamiquela, 1967, a basal dinosauriform from the Late Triassic of Argentina. *Journal of Systematic Palaeontology* **16**, 853–879 (2017).

2. Casamiquela, R. M. Un nuevo dinosaurio ornitisquio Triásico (*Pisanosaurus mertii*; Ornithopoda) de la Formación Ischigualasto, Argentina. *Ameghiniana* **5**, 47–64 (1967).

3. Bonaparte, J. F. *Pisanosaurus mertii* Casamiquela and the origin of the Ornithischia. *Journal of Paleontology* **50**, 808–820 (1976).

4. Bonaparte, J. F. Annotated list of the South American Triassic tetrapods. *Council of Scientific and Industrial Research* **2**, 665–682 (1970).

5. Baczko, M. B. von. Rediscovered cranial material of *Venaticosuchus rusconii* enables the first jaw biomechanics in Ornithosuchidae (Archosauria: Pseudosuchia). *Ameghiniana* **55**, 365–380 (2018).

6. Bonaparte, J. F. El Mesozoico de América del Sur y sus tetrápodos. Ministerio de Cultura y Educación, Fundación Miguel Lillo, San Miguel de Tucumán, 596 pp. (1978).

7. Reig, O. A. La presencia de dinosaurios saurisquios en los "Estratos de Ischigualasto" (Mesotriásico superior) de las provincias de San Juan y La Rioja (Argentina). *Ameghiniana* **3**, 3–20 (1963).

8. Lecuona, A., Ezcurra, M. D. & Irmis, R. B. Revision of the early crocodylomorph *Trialestes romeri* (Archosauria, Suchia) from the lower Upper Triassic Ischigualasto Formation of Argentina: one of the oldest-known crocodylomorphs. *Papers in Palaeontology* **2**, 585–622 (2016).

9. Martínez, R. N., Apaldetti, C., Alcober, O. A., Colombi, C. E., Sereno, P. C., Fernández, E., Malnis, P. S., Correa, G. A. & Abelin, D. Vertebrate succession in the Ischigualasto Formation. *Journal of Vertebrate Paleontology* **32**: sup1, 10–30 (2013).

10. Colombi, C. E., Rogers, R. R. & Alcober, O. A. Vertebrate taphonomy of the Ischigualasto Formation. *Journal of Vertebrate Paleontology* **32**: sup1, 31–50 (2013).

11. Bonaparte, J. F. Faunal replacement in the Triassic of South America. *Journal of Vertebrate Paleontology* **2**, 362–371 (1982).

12. Bonaparte, J. F. El Triásico de San Juan—La Rioja Argentina y sus Dinosaurios. Museo Argentino de Ciencias Naturales. Buenos Aires, 196 pp. (1997).

13. Bonaparte, J. F. Classification of the Thecodontia. *Geobios* **15**, 99–112 (1982).

14. Sereno, P. C. Taxonomy, morphology, masticatory function and phylogeny of heterodontosaurid dinosaurs. ZooKeys **226**, 1–225 (2012).

15. Martins-Neto, M. A. & Catuneanu, O. Rift sequence stratigraphy. *Marine and Petroleum Geology* **27**, 247–253 (2010).

16. Colombi, C. E., Limarino, C. O. & Alcober, O. A. Allogenic controls on the fluvial architecture and fossil preservation of the Upper Triassic Ischigualasto Formation, NW Argentina. *Sedimentary Geology* **362**, 1–16 (2017).

17. Simms, M. J. & Ruffell, A. H. Synchroneity of climatic change and extinctions in the Late Triassic. *Geology* **17**, 265–268 (1898).

18. Roghi, G., Gianolla, P., Minarelli, L., Pilati, C. & Preto, N. Palynological correlation of Carnian humid pulses throughout western Tethys. *Palaeogeography, Palaeoclimatology, Palaeoecology* **290**, 89–106 (2010).

19. Ezcurra, M. D. Comments on the taxonomic diversity and paleobiogeography of the earliest known dinosaur assemblages (late Carnian–earliest Norian). *Historia Natural, tercera serie* **2**, 49–71 (2012).

20. Benton, M. J.; Bernardi, M. & Kinsella, C. The Carnian Pluvial Episode and the origin of dinosaurs. *Journal of the Geological Society* **175**, 1019–1026 (2018).

21. Bernardi, M., Gianolla, P., Petti, F. M., Mietto, P. & Benton, M. J. Dinosaur diversification linked with the Carnian Pluvial Episode. *Nature Communications* **9**, 1499 (2018).

22. Rogers, R. R., Swisher, C. C., III, Sereno, P. C., Monetta, A. M., Forster, C. A. & Martinez, R. N. The Ischigualasto Tetrapod Assemblage (Late Triassic, Argentina) and $^{40}Ar/^{39}Ar$ Dating of Dinosaur Origins. *Science* **260**, 794–797 (1993).

23. Tabor, N. J., Montañez, I. P., Kelso, K. A., Currie, B. S., Shipman, T. A., & Colombi, C. E. A Late Triassic soil catena: landscape and climate controls on paleosol morphology and chemistry across the Carnian-age Ischigualasto-Villa Unión basin, northwestern Argentina. In: Alonso-Zarza, A. A. & Tanner, L. H. (eds.), *Paleoenvironmental record and applications of calcretes and palustrine carbonates*. Geological Society of America Special Paper **416** (2006).

24. Martínez, R. N., Sereno, P. C., Alcober, O. A., Colombi, C. E., Renne, P. R., Montañez, I. P. & Currie, B. S. A basal dinosaur from the dawn of the dinosaur era in southwestern Pangaea. *Science* **331**, 201–210 (2011).

25. Ramezani, J., Hoke, G. D., Fastovsky, D. E., Bowring, S. A., Therrien, F., Dworkin, S. I., Atchley, S. C. & Nordt, L. C. High-precision U-Pb zircon geochronology of the Late Triassic Chinle Formation, Petrified Forest National Park (Arizona, USA): Temporal constraints on the early evolution of dinosaurs. *Geological Society of America Bulletin* **123**, 2142–2159 (2011).

26. Hoke, G. D., Schmitz, M. D. & Bowring, S. A. An ultrasonic method for isolating nonclay components from clay-rich material. *Geochemistry, Geophysics, Geosystems* **15**, 492–498 (2014).

27. Mattinson, J. M. Zircon U/Pb chemical abrasion (CA-TIMS) method; combined annealing and multi-step partial dissolution analysis for improved precision and accuracy of zircon ages. *Chemical Geology* **220**, 47–66 (2005).

28. Condon, D. J., Schoene, B., McLean, N. M., Bowring, S. A. & Parrish, R. R. Metrology and traceability of U-Pb isotope dilution geochronology (EARTHTIME Tracer Calibration Part I). *Geochimica et Cosmochimica Acta* **164**, 464–480 (2015).

29. McLean, N. M., Condon, D. J., Schoene, B. & Bowring, S. A. Evaluating uncertainties in the calibration of isotopic reference materials and multi-element isotopic tracers (EARTHTIME Tracer Calibration Part II). *Geochimica et Cosmochimica Acta* **164**, 481–501 (2015).

30. Krogh, T. E. Low-Contamination Method for Hydrothermal Decomposition of Zircon and Extraction of U and Pb for Isotopic Age Determinations. *Geochimica et Cosmochimica Acta* **37**, 485–494 (1973).

31. Hiess, J., Condon, D. J., McLean, N. & Noble, S. R. $^{238}U/^{235}U$ Systematics in Terrestrial Uranium-Bearing Minerals. *Science* **335**, 1610–1614 (2012).

32. Bowring, J. F., McLean, N. M. & Bowring, S. A. Engineering cyber infrastructure for U-Pb geochronology: Tripoli and U-Pb_Redux. *Geochemistry Geophysics Geosystems* **12** (2011).

33. McLean, N. M., Bowring, J. F. and Bowring, S. A. An algorithm for U-Pb isotope dilution data reduction and uncertainty propagation. *Geochemistry Geophysics Geosystems* **12** (2011).

34. Jaffey, A. H., Flynn, K. F., Glendenin, L. E., Bentley, W. C. & Essling, A. M. Precision Measurement of Half-Lives and Specific Activities of ^{235}U and ^{238}U. *Physical Review C* **4**, 1889–1906 (1971).

35. Haslett, J. & Parnell, A. A simple monotone process with application to radiocarbon-dated depth chronologies. *Journal of the Royal Statistical Society Series C-Applied Statistics* **57**, 399–418 (2008).

36. Parnell, A. C., Haslett, J., Allen, J. R. M., Buck, C. E. & Huntley, B. A flexible approach to assessing synchroneity of past events using Bayesian reconstructions of sedimentation history. *Quaternary Science Reviews* **27**, 1872–1885 (2008).

37. R Core Team. *R: A language and environment for statistical computing*. R Foundation for Statistical Computing, Vienna. http://www.r-project.org/index.html (2019).
