# Supplementary material for: The Late Triassic Ischigualasto Formation at Cerro Las Lajas (La Rioja, Argentina): fossil tetrapods, high-resolution chronostratigraphy, and faunal correlations
Source: Sci Rep. 2020 Jul 29;10:12782. doi: 10.1038/s41598-020-67854-1 (PMC7391656; doi:10.1038/s41598-020-67854-1)
Supplement: Supplementary file 2 — Supplementary file2 [file 41598_2020_67854_MOESM2_ESM.pdf]

Table S2. U-Pb isotopic data for analyzed zircons from tuff samples of the Ischigualasto Formation at Hoyada del Cerro Las Lajas.

	Composition					Ratios							Age (Ma)				
Sample Fractions†	Pb_c (pg)	$Pb^{*‡}$/Pb_c	U‡ (pg)	Th/U	$^{206}Pb^{§}$/^{204}Pb	$^{208}Pb^{\#}$/^{206}Pb	$^{206}Pb^{††}$/^{238}U	err (2σ%)	$^{207}Pb^{††}$/^{235}U	err (2σ%)	$^{207}Pb^{††}$/^{206}Pb	err (2σ%)	^{206}Pb/^{238}U	err (2σ)	^{207}Pb/^{235}U	^{207}Pb/^{206}Pb	corr. coef.
**Toba-2 (107 meters above base)**																	
**z6**	0.2	23.5	92	1.5	1108.9	0.494	0.036237	(.11)	0.25600	(1.11)	0.05126	(1.08)	**229.46**	**0.24**	231.4	252	0.34
**z5**	0.2	18.5	74	1.6	870.3	0.506	0.036212	(.13)	0.25669	(1.40)	0.05143	(1.36)	**229.31**	**0.30**	232.0	259	0.34
**z4**	0.2	41.5	170	1.7	1875.2	0.554	0.036206	(.08)	0.25384	(.68)	0.05087	(.66)	**229.27**	**0.18**	229.7	234	0.29
**z1**	0.3	13.5	82	1.1	704.3	0.366	0.036181	(.16)	0.25312	(1.78)	0.05076	(1.73)	**229.12**	**0.37**	229.1	229	0.35
**z2**	0.2	21.3	111	1.0	1130.2	0.321	0.036181	(.11)	0.25296	(1.13)	0.05073	(1.09)	**229.12**	**0.24**	229.0	228	0.33
**z7**	0.3	25.2	148	1.7	1146.8	0.549	0.036177	(.14)	0.25312	(1.11)	0.05077	(1.07)	**229.09**	**0.32**	229.1	229	0.33
**LL041219-2 (160 meters above base)**																	
z8	0.6	19.5	218	0.8	1068.9	0.282	0.044232	(.12)	0.31888	(1.12)	0.05231	(1.09)	279.01	0.33	281.0	298	0.31
z3	0.4	9.6	92	0.6	565.9	0.208	0.037897	(.22)	0.26887	(2.24)	0.05148	(2.17)	239.78	0.51	241.8	261	0.34
z1	0.3	17.5	106	2.4	707.7	0.773	0.036365	(.17)	0.25640	(1.77)	0.05116	(1.73)	230.26	0.39	231.8	247	0.29
z2	0.3	11.5	77	1.0	622.7	0.319	0.036300	(.18)	0.25629	(1.98)	0.05123	(1.93)	229.86	0.41	231.7	250	0.32
**z4**	0.4	12.6	80	2.5	509.4	0.791	0.036217	(.24)	0.25617	(2.40)	0.05132	(2.33)	**229.34**	**0.54**	231.6	254	0.30
**z9**	0.4	8.0	72	0.7	464.9	0.237	0.036196	(.26)	0.25556	(2.86)	0.05123	(2.77)	**229.21**	**0.59**	231.1	250	0.36
**z5**	0.3	16.9	94	2.4	688.5	0.758	0.036179	(.20)	0.25593	(1.86)	0.05133	(1.80)	**229.11**	**0.46**	231.4	255	0.34
**z6**	0.7	20.3	255	2.1	853.6	0.692	0.036117	(.14)	0.25313	(1.48)	0.05085	(1.44)	**228.72**	**0.31**	229.1	233	0.33
**LL041219-6 (1035 meters above base)**																	
z5	0.4	9.1	88	1.1	478.0	0.366	0.035803	(.25)	0.25363	(2.60)	0.05140	(2.53)	226.77	0.55	229.5	258	0.32
z3	0.3	3.7	30	0.6	232.8	0.194	0.035492	(.51)	0.25497	(5.56)	0.05213	(5.41)	224.8	1.1	231	290	0.35

z1	0.3	21.1	172	0.7	1208.5	0.224	0.035128	(.12)	0.24520	(1.03)	0.05065	(1.00)	222.56	0.27	222.7	224	0.30
**z2**	0.3	25.6	209	0.6	1485.3	0.204	0.035032	(.11)	0.24444	(.96)	0.05063	(.93)	**221.96**	**0.24**	222.0	223	0.29
**z7**	0.5	7.2	91	0.9	402.6	0.292	0.035026	(.28)	0.25192	(3.04)	0.05219	(2.96)	**221.93**	**0.61**	228.1	293	0.33
**z4**	0.3	69.9	591	0.6	4054.5	0.194	0.035004	(.06)	0.24456	(.33)	0.05070	(.31)	**221.79**	**0.12**	222.15	226.0	0.31
**z8**	0.4	10.7	103	0.5	647.8	0.168	0.035000	(.19)	0.24774	(1.92)	0.05136	(1.87)	**221.77**	**0.40**	224.7	256	0.33

Notes: Corr. coef. = correlation coefficient. Age calculations are based on the decay constants of Jaffey et al. (1971).

† All analyses are single zircon grains and pre-treated by the thermal annealing and acid leaching (CA-TIMS) technique. Data used in age calculations are in bold.

‡ Pb_c is total common Pb in analysis. Pb* is radiogenic Pb concentration. Total sample U is in picograms.

§ Measured ratio corrected for spike and fractionation only.

# Radiogenic Pb ratio.

†† Corrected for fractionation, spike, blank, and initial Th/U disequilibrium in magma (Th/U_{magma} = 2.8). Mass fractionation correction of 0.18%/amu ± 0.04%/amu (atomic mass unit) was applied to single-collector Daly analyses. All common Pb is assumed to be blank. Total procedural blank was less than 0.1pg for U. Blank isotopic composition: $^{206}Pb/^{204}Pb$ = 18.15 ± 0.47, $^{207}Pb/^{204}Pb$ =15.30 ± 0.30, $^{208}Pb/^{204}Pb$ = 37.11 ± 0.87.
